# Double trouble: trypanosomatids with two hosts have lower infection prevalence than single host trypanosomatids

**DOI:** 10.1093/emph/eoad014

**Published:** 2023-05-16

**Authors:** Hawra Al-Ghafli, Seth M Barribeau

**Affiliations:** Department of Evolution, Ecology and Behaviour, Institute of Infection, Veterinary, and Ecological Sciences, University of Liverpool, Liverpool, UK; Department of Evolution, Ecology and Behaviour, Institute of Infection, Veterinary, and Ecological Sciences, University of Liverpool, Liverpool, UK; Ozette Technologies, Seattle, WA, USA

**Keywords:** monoxenous, dixenous, trypanosomatids, infection prevalence, life cycle, meta-analysis, natural language processing, life history, evolution, adaptation

## Abstract

Trypanosomatids are a diverse family of protozoan parasites, some of which cause devastating human and livestock diseases. There are two distinct infection life cycles in trypanosomatids; some species complete their entire life cycle in a single host (monoxenous) while others infect two hosts (dixenous). Dixenous trypanosomatids are mostly vectored by insects, and the human trypanosomatid diseases are caused mainly by vectored parasites. While infection prevalence has been described for subsets of hosts and trypanosomatids, little is known about whether monoxenous and dixenous trypanosomatids differ in infection prevalence. Here, we use meta-analyses to synthesise all published evidence of trypanosomatid infection prevalence for the last two decades, encompassing 931 unique host-trypansomatid systems. In examining 584 studies that describe infection prevalence, we find, strikingly, that monoxenous species are two-fold more prevalent than dixenous species across all hosts. We also find that dixenous trypanosomatids have significantly lower infection prevalence in insects than their non-insect hosts. To our knowledge, these results reveal for the first time, a fundamental difference in infection prevalence according to host specificity where vectored species might have lower infection prevalence as a result of a potential ‘jack of all trades, master of none’ style trade-off between the vector and subsequent hosts.

## INTRODUCTION

Trypanosomatids are Kinetoplastid protozoan parasites that infect a diverse range of hosts including vertebrates, invertebrates and plants. Among trypanosomatids, there are two distinct life-histories, with some infecting only a single host (monoxenous), and others that require two different hosts to complete their development (dixenous).The dixenous lifestyle is thought to be evolutionarily derived and has evolved independently multiple times among the Kinetoplastids [1–3]. Dixenous trypanosomatids, which are currently classified into five different genera (i.e. *Leishmania, Trypanosoma, Endotrypanum, Porcisia* and *Phytomonas*) mostly include an insect vector that then transmits infection to a vertebrate or plant host. The vast majority of epidemiological and empirical evidence for all trypanosomatids comes from the two medically relevant genera (*Leishmania* and *Trypanosoma*). At least 20 species of *Leishmania*, cause cutaneous, mucocutaneous and visceral leishmaniasis; *Trypanosoma cruzi* causes Chagas disease and *Trypanosoma brucei* is the etiological agent of African sleeping sickness. These diseases cause more than 830,000 infections every year [4] and their control is challenging. These parasites cause significant disease burden. Estimates of incidence and mortality vary for these diseases, but some studies predict that more than 1 million people contract leishmaniasis annually, including up to 700,000 visceral cases (with a fatality rate of 95%), and a further 1 billion people are at risk of infection [5, 6].

Monoxenous trypanosomatids not only have a simpler life-cycle and narrower host specificity, usually in a single invertebrate host, but they are also more common [7], more ancient (but see) [8], and more diverse than dixenous trypanosomatids [9]. There are 17 currently described monoxenous trypanosomatid genera: *Angomonas, Blastocrithidia, Blechomonas, Crithidia, Herpetomonas, Jaenimonas, Kentomonas, Lafontella, Leptomonas, Lotmaria, Novymonas, Paratrypanosoma, Rhynchoidomonas, Sergeia, Strigomonas, Wallacemonas* and *Zelonia* in contrast to the five dixenous genera. Despite the overwhelming diversity of monoxenous trypanosomatids we know comparatively little about their natural history and prevalence, due to their perceived lack of medical relevance—although this has been called into question recently by opportunistic vertebrate infections by some monoxenous trypanosomatids [10–12].

Interestingly, monoxenous and dixenous trypanosomatids also appear to differ in their infection prevalence. Compared to monoxenous trypanosomatids, the prevalence of dixenous trypanosomatids in insect hosts is much lower. For instance, fewer than 3% of Phlebotomus sand flies carry *Leishmania* [13] and as few as 0.1% of adult tsetse flies have transmissible *Trypanosoma* cells in their saliva [14]. In contrast, trypanosomatids with only a single insect host can reach extremely high prevalence. In the bumblebee *Bombus terrestris* up to 35% of workers can be infected by its trypanosomatid parasite, *Crithidia bombi* [15]. These data hint at something crucial: that perhaps the major limiting factor for trypanosomatid infection transmission is the vector, not the vertebrate host. Low infection prevalence among insect vectors seems relatively consistent across other non-trypanosomatid dixenous pathogens and parasites such as etiological agents of malaria (0.4–2.1%) [16], Lyme disease (0.7–2.6%) [17], tick-borne encephalitis (0.23–0.28%) [18] and West Nile virus (WNV) (below 0.1%) [19]. This low prevalence in vectors can, in the case of malaria, be partially explained by the low prevalence of transmissible parasite in the peripheral blood (only 2–3% are capable of transmitting to insects by differentiating to sexual gametocytes) [20]. The epidemiological features of vectored trypanosomatids, like other vectored diseases, are subject to fundamentally different evolutionary pressures than monoxenous parasites, that may explain these differences in infection prevalence. While this low prevalence has been described in diverse insect vectored diseases, there has been no systematic attempt to compare the infection prevalence of dixenous parasites and their monoxenous relatives, nor would this approach be feasible in every system as many lack closely related monoxenous taxa.

Trade-offs imposed by life-history strategies are central to evolutionary biology as a hypothetical organism that maximizes all aspects of fitness simultaneously would immediately dominate the population eradicating diversity (the Darwinian demon) [20]. The lack of such demons points to the universality of trade-offs among fitness traits as improvements in one fitness relevant trait negatively impacts another fitness relevant traits [20]. In the case of parasites, fitness is affected by differences in growth stages, host specificity, transmission modes and virulence [21, 22]. Each of these features of parasite biology can affect epidemiology and each may have evolutionary context dependent optima [21, 22]. For instance, allopatric adaptation of *Podosphaera plantaginisit*, the obligate fungal pathogen, alters the shape of the trade-off between pathogens life-history traits. Whereby, unlike infections on sympatric host populations, allopatric adaptation results in negative correlation between pathogens’ infectivity and transmission-related traits (i.e. time to sporulation and time to germination) and are associated with significantly lower infection prevalence [23]. Further, mathematical modelling and empirical evidence suggest that the shape of life-history traits of a pathogen (i.e. transmission-virulence trade-off) is likely linked with spatial epidemiology; in which, high local infections/transmissions are likely to select for lower virulence [24–27]. In contrast, moderate levels of local infections/transmissions may select for higher virulence [24–27].

Parasitic worms also vary in the complexity in their life cycle, some of which use a single-host and others require multiple hosts. This variation in life history may affect other parasite traits, including transmission and prevalence [28, 29] but has not been tested in single versus multi-host worms. In addition, the fitness of *Schistosoma mansoni* in their intermediate host (infection intensity and transmission potential) is negatively correlated with their fitness in their second host, suggesting a trade-off between adaptation to obligate hosts [22]. This inverse relationship in parasite fitness of *Schistosoma mansoni* between obligate hosts could be due to genetically based trade-off (i.e. genetic-based constraints due to fluctuating selection over the course of the life cycle) [22]. Here, we examine whether complexity of parasite life cycle predicts infection prevalence. This is crucial to our understanding of parasite biology, epidemiology and how to further customize control strategies based on different parasite life-history traits.

Trypanosomatids provide an excellent system to address fundamental questions about the potential consequences of having a complex life history. Here, we take advantage of the relatively close phylogenetic relationships between monoxenous and dixenous trypanosomatids and systematically test whether monoxenous and dixenous trypanosomatids indeed have different infection prevalence, and whether this is consistent among hosts.

## METHODOLOGY

### Literature search

We searched Scopus and all available databases in Web of Science, to identify all published studies that describe trypanosomatid prevalence in non-human hosts (identifying 1511 references in Scopus and 5414 from Web of Science). Full search terms can be found in Supplementary Table 1. During the literature search, we did not apply any year or publication type restrictions (search stopped January 2020). To exhaustively find studies of neglected trypanosomatids, we further checked the references in relevant papers and added candidate citations to the screening list of studies. All duplicate references were removed.

### Relevant screening stages and design

**Figure 1.**
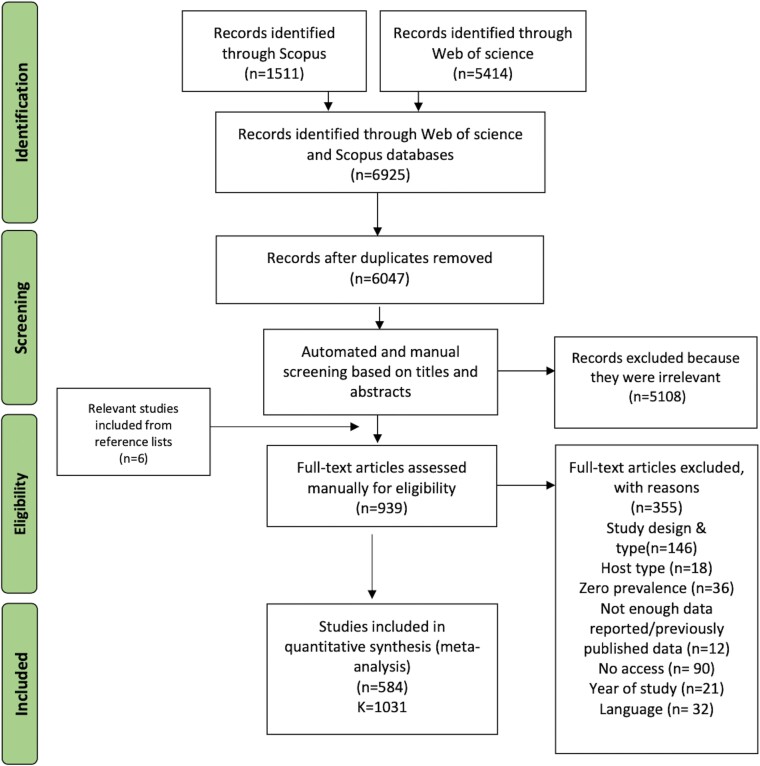
Selection and screening stages. The overall protocol utilised for literature search and screening, where (*n*) represents the number of studies included/excluded at each stage and (*K*) represents the number of data points extracted from included studies. All steps of the systematic review were in concordance with PRISMA guidelines.

References were initially screened based on titles and abstracts manually by a single reviewer (HA) using Rayyan website as outlined in (Supplementary Appendix). We further employed an automated second review protocol using Machine Learning Algorithm (MLA) and Natural Language Processing (NLP) during the initial screening phase, as has previously been suggested for large datasets (and detailed in (Supplementary Appendix)). This was done to identify potentially missed studies by human reviewers before full-text screening. All studies that received a manual or an automated ‘include’ decision by human reviewer or ML were subsequently subjected to further eligibility assessment by full-text review by two different reviewers (Fig. 1). Inclusion and exclusion criteria, and detailed protocol of study selection and data extraction can be found in (Supplementary Appendix).

### Meta-analysis

We extracted the infection prevalence, diagnostic method, country of study, species of parasite and host and year of publication from all included studies. As host species was not always provided, we simplified hosts to broad host categories (i.e. pigs, dogs, cats, camels, cattle, sheep, fish, raccoons, buffaloes, rodents, true bugs, flies, bees, birds, bats, fleas) when comparing across host groups. The proportion of infection values were first transformed with escalc() function and (measure = ‘PFT’) to stabilise the variance by Freeman–Tukey double arcsine transformation. Subsequently, meta-regressions were conducted on a transformed scale using ‘metafor’ package. Pooled estimates (weighted proportions) were then back-transformed for each analysis and multiplied by 100 to get the pooled prevalence. The pooled prevalence can be interpreted as weighted summary of infection prevalence for that group. Publication bias was assessed using a funnel plot with trim and fill method and Egger’s test with sample size as a predictor. We employed a random effect multilevel model (rma.mv) with a nested random design to account for the heterogeneity among studies (e.g. study protocols and/or different host/parasite taxa within and between studies). We assigned ‘study ID’ as an outer random factor, and both host type (19 levels) and parasite taxa (50 levels) were considered inner-random factors. Each model was fitted with restricted maximum-likelihood (‘REML’). This was done for the general model with no moderator and meta-regressions with the following moderators: diagnostic method (three levels: ‘molecular’, ‘microscopic and culture based’, ‘serological’), life-history traits of the parasite (three levels: dixenous, monoxenous, mixed trypanosomatids), host groups (insects and non-insects), parasite taxa (50 levels), host taxa (19 levels). We also ran similar meta-regression models to assess pooled prevalence on subgroups of interest (as detailed in Supplementary Table 2).

Between-study variation was assessed using the Higgins I2 statistic using (rma or ma.uni), which usually indicates substantial heterogeneity if exceeding 50%. The QM statistic, an omnibus test of all model coefficients except for the intercept, was calculated to conclude if a moderator significantly influences the mean effect size. All meta-regression models were run with and without the model intercept to statistically compare effect size of factors and generate pooled estimate for each factor respectively. We used a stringent Benjamini–Hochberg method to account for elevated Type 1 error from multiple testings (Supplementary Table 3). Forest plots were presented using orchaRd package with tanh transformation as traditional forest plot will fail to present the large number of studies used here. Data sources for each analysis are outlined in Supplementary Appendix.

We used several distinct meta-regressions (presented in Supplementary Table 2) to address particular questions. In most models, we ran the meta-regression both without, and with an intercept allowing us to compare levels of the moderator using t-tests. Each meta-regression asks a specific question of these data. In short, model A examined variation in infection prevalence among parasite taxa, model B asks whether parasites vary in infection prevalence depending on whether they infect a single host or two hosts and model C compares infection prevalence in insects to non-insect hosts. The remaining models further delve into differences within host or parasite taxa (Supplementary Table 2). Models F1 and F2 examine variation in diagnostic methods in insect (F1) or non-insect (F2) hosts; F3-F5 compare dixenous to monoxenous trypanosomatids in all insects, flies or true bugs, respectively; F6-F9 compare prevalence of insect hosts to non-insect hosts in different levels of dixenous trypanosomatids (all dixenous trypanosomatids F6, *Leishmania* spp. F7, *Trypanosoma* spp. excluding *T. cruzi* F8, *T. cruzi* only F9) and F10–F12 examine wild vs managed bees combined, bumblebees and honeybees, respectively. All models included study ID as an outer random factor while the inner random factors varied (A-F5: host type and parasite type; F6-9: parasite taxa; F10-F12: bee taxa and parasite taxa). All models (except A) included an intercept, which allows us to statistically compare between inner factors of the moderator with *t*-test. We did not include an intercept in meta-regression A as we were less concerned about how much each level differed, but rather the overall effect of a moderator on infection prevalence.

Further details about the literature searches, meta-regressions and code to replicate this work can be found in the Supplementary Methods, and in our Github repository: https://github.com/Hawra480/Meta.

## RESULTS

**Figure 2.**
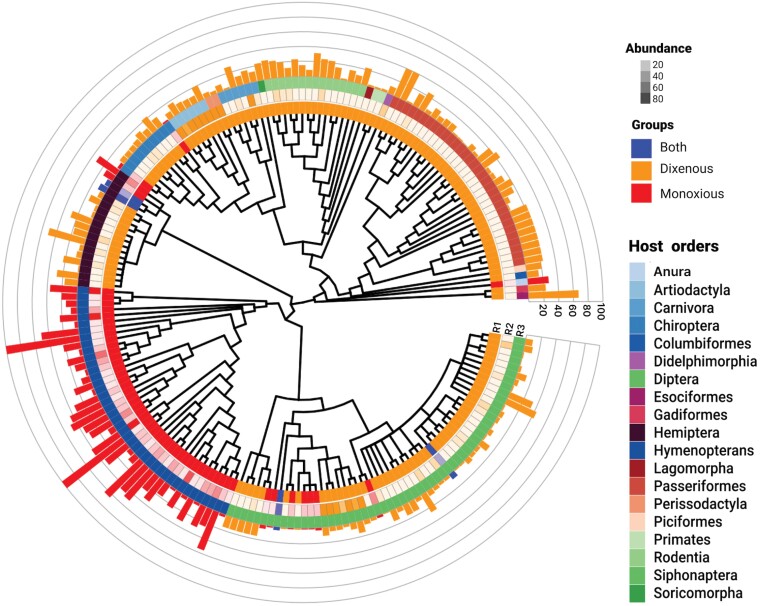
A visual summary of all included host-trypanosomatid systems. A phylogenetic representation of all host taxa examined in this study (centre). The first (inner) ring (R1) illustrates the parasites’ life history (dixenous, monoxenous or both), the second ring (R2) highlights the abundance of prevalence data for each taxon, and the third ring (R3) is host order. Prevalence of infection (%) is shown in the outer scaled ring, which is coloured according to parasite life history.

From our initial input of 6046 unique citations, we retained 584 studies after full-text screening. In general, these studies demonstrate a wide geographic distribution of trypanosomatids (Supplementary Appendix) among a diverse range of host-taxa (Fig. 2). For summary purposes and to capitalize on this large data-set, we show a summary of infection prevalence among different parasite-host systems in (Supplementary Fig. 2), subsequent analysis of pooled estimate of various host-groupings in (Supplementary Fig. 7), infection prevalence stratified by parasite species in (Table 1) or by host type in (Supplementary Fig. 4). Some host groups (e.g. fish, raccoons and buffaloes) have a very high pooled prevalence although they are comparatively rarely studied (Supplementary Fig. 4). In spite of the low number of studies, their total sample size indicates comparable robustness of analysis to that of the otherwise heavily examined host groups for trypanosomatid infections (Supplementary Fig. 7).

**Table 1.** Summary of all included studies based on parasite groupings

Parasite grouping	Parasite life-cycle classification	Host order	Infected hosts	Geographic distribution	Meta-analysis studies	Kmeta	Sig	Pooled estimate (ci·il, ci·ul)	SE	Pooled prevalence (%)
‘Jaculum’ spp.	Monoxenous	Hemiptera	Insects {true bugs}	South America	1	4	**	0.583 (0.214, 0.952)	0·188	30.0
*Agomonas* spp.	Monoxenous	Diptera	Insects {flies}	South and Central America, Africa	1	3		NA	NA	NA
*Blastocrithidia* spp.	Monoxenous	Hemiptera, Chiroptera	Insects {true bugs}, Non-insects {bats}	South and North America	3	5	*	0.278 (0.062, 0.450)	0·110	6.99
*Crithidia bombi*	Monoxenous	Hymenoptera	Insects {bees}	South and North America, Europe	18	80	***	0.560 (0.463, 0.676)	0·0543	28.8
*Crithidia expoeki*	Monoxenous	Hymenoptera	Insects {bees}	North America	2	10		0.155 (−0.118, 0.427)	0·139	1.79
*Crithidia mellificae*, *Lotmaria passim*	Monoxenous	Hymenoptera	Insects {bees}	Europe, South and North Amarica, Asia and Africa	12	18	***	0.545 (0.415, 0.671)	0·054	26.6
*Crithidia mexicana*	Monoxenous	Hymenoptera	Insects {bees}	North America	1	8	·	0.350 (−0.025, 0.724)	0·191	11.2
*Crithidia* spp.	Monoxenous	Hymenoptera, Diptera	Insects {bees, flies}	North America, Europe, Asia	3	8	**	0.313 (0.079, 0.547)	0·120	8.95
Fish trypanosomes	Dixenous	Acipenseriformes	Non-insects {fish}	Europe, Australia, South America, Africa.	8	10	***	0.8618 (0.6157, 1.1080)	0.126	57.6
*Herpetomonas* spp.	Monoxenous	Diptera	Insects {flies}	South America	1	1		NA	NA	NA
*Leishmania amazonensis*	Dixenous	Diptera	Insects {flies}	South America	1	1		NA	NA	NA
*Leishmania braziliensis*	Dixenous	Diptera, Carnivora	Insects {flies}, Non-insects {dogs}	South and North America and Asia	12	12	***	0.348 (0.225, 0.471)	0·063	11.1
*Leishmania donovani*	Dixenous	Diptera, Carnivora and others	Insects {flies}, Non-insects{dogs, and multiple other hosts}	Asia and Africa	5	5	**	0.315 (0.111, 0.518)	0·104	9.04
*Leishmania guyanensis*	Dixenous	Diptera, Carnivora	Insects {flies}, Non-insects {dogs}	South America	2	3	*	0.287 (0.011, 0.564)	0·141	7.47
*Leishmania infantum*	Dixenous	Diptera, Carnivora, Chiroptera and others	Insects {flies}, Non-insects {dogs, cats, rodents, equids, bats, oppossums, other animals}	South and North America, Europe, Africa, Asia	123	130	***	0.383 (0.343, 0.424)	0·021	13.5
*Leishmania major*	Dixenous	Diptera, Carnivora, Rodentia and others	Insects {flies}, Non-insects {dogs, cats, rodents, birds, other animals}	South America, Asia, Africa	14	21	***	0.345 (0.231, 0.460)	0·058	11.0
*Leishmania mexicana*	Dixenous	Carnivora, Chiroptera, Rodentia	Non-insects {rodents, bats, dogs}	South and North America	4	4	***	0.434 (0.235, 0.634)	0·102	17.24
*Leishmania* spp.	Dixenous	Diptera, Carnivora, Rodentia and others	insects {flies}, Non-insects {dogs, cats, rodents, other animals}	South America, Africa, Europe	97	123	***	0.406 (0.360, 0.450)	0.0231	15.1
*Leishmania tropica*	Dixenous	Diptera, Carnivora	insects {flies}, Non-insects {dogs, cats}	Asia and Africa	5	5	*	0.244 (−0.056, 0.432)	0·096	5.28
*Leishmania* *turanica*	Dixenous	Rodentia	Non-insects {rodents}	Asia and Africa	1	1		NA	NA	NA
*Leptomonas* spp.	Monoxenous	Siphonaptera, Hemiptera	Insects {true bugs, fleas}	South America	2	5	·	0.256 (−0.035, 0.55)	0·148	5.86
monoxenous spp.	Monoxenous	Hymenoptera, Hemiptera	Insects {bees, true bugs}	Asia	2	2	***	0.601 (0.30, 0.91)	0·155	31.8
*Phytomonas* spp.	Dixenous	Hemiptera	Insects {true bugs}	South America, Asia	3	6	*	0.277 (0.055, 0.50)	0.015	6.90
*Trypanosoma avium*	Dixenous	Siphonaptera, Passeriformes	Insects {fleas}, Non-insects {birds}	Australia, Europe, North America	4	4	**	0.321 (−0.102, 0.54)	0·112	9.44
*Trypanosoma brucei*	Dixenous	Diptera, Carnivora, Artiodactyla and others	Insects {flies}, Non-insects {dogs, cattle, sheep, goats, equids and others}	Africa and Asia	40	58	***	0.230 (0.167, 0.291)	0·031	4.60
*Trypanosoma chelodinae*	Dixenous	NA	Non-insects {animals}	Australia	1	1	NA	NA	NA	NA
*Trypanosoma congolense*	Dixenous	Diptera, Carnivora, Artiodactyla and others	Insects {flies}, Non-insects {dogs, cattle, sheep, goats, equids and others}	Africa	57	97	***	0.287 (0.235, 0.339)	0·029	7.44
*Trypanosoma copemani*	Dixenous	NA	Non-insects {animals}	Australia	1	1	NA	NA	NA	NA
*Trypanosoma cruzi*	Dixenous	Hemiptera, Chiroptera, Carnivora	Insects {true bugs}, Non-insects {dogs, cats, bats}	South, Central and n North America	82	94	***	0.466(0.418, 0.51)	0·0245	19.80
*Trypanosoma culicavium*	Dixenous	Diptera	Insects {flies}	Australia	1	2		NA	NA	NA
*Trypanosoma dionisii*	Dixenous	Chiroptera	Non-insects {bats}	South and North America	3	5	***	0.48 (0.264, 0.70)	0·110	21.0
*Trypanosoma equiperdum*	-	Perissodactyla	Non-insects {equids}	Asia	1	1		NA	NA	NA
*Trypanosoma evansi*	-	Artiodactyla, Perissodactyla and others	Non-insects {equids, camels, dogs, cattle, goats, sheep, buffaloes and animals}	South America, Africa, Asia and Europe	63	77	***	0.371 (0.317, 0.426)	0·028	12.67
*Trypanosoma everetti*	Dixenous	Passeriformes	Non-insects {birds}	Africa	1	1		NA	NA	NA
*Trypanosoma evotomys*	Dixenous	Rodentia	Non-insects {rodents}	Europe	1	1	*	NA	NA	NA
*Trypanosoma giganteum*	Dixenous	NA	Non-insects {animals}	Europe	1	1		NA	NA	NA
*Trypanosoma gilletti*	Dixenous	NA	Non-insects {animals}	Australia	1	1		NA	NA	NA
*Trypanosoma godfreyi*	Dixenous	Diptera, Artiodactyla	Insects {flies}, Non-insects {pigs}	Africa	4	7	*	0.212 (0.04, 0.385)	0·09	3.86
*Trypanosoma lewisi*	Dixenous	Rodentia	Non-insects {rodents}	Asia and Africa	2	2	**	0.511 (0.230, 0.792)	0·143	23.6
*Trypanosoma noyesi*	Dixenous	NA	Non-insects {animals}	Australia	1	1		NA	NA	NA
*Trypanosoma rangeli*	Dixenous	Hemiptera, Chiroptera	Insects {true bugs}, Non-insects {bats}	North and South America	3	3		0.163 (−0.059, 0.385)	0·122	2.05
*Trypanosoma simiae*	Dixenous	Siphonaptera, Artiodactyla, Perissodactyla	Insects {fleas}, Non-insects {cattle, equids, pigs}	Africa	9	18	**	0.281 (0.164, 0.399)	0·065	7.16
*Trypanosoma* spp.	Dixenous	Diptera, Artiodactyla, Passeriformes, Rodentia, Hemiptera, Chiroptera, Acipenseriformesand others	Insects {true bugs, flies}, Non-insects {cattle, rodents, bats, pigs, birds, fish, equids, and others}	South and North America, Europe, Africa, Asia and Australia	63	69	***	0.377 (0.322, 0.432)	0·03	13.1
*Trypanosoma theileri*	Dixenous	Artiodactyla	Non-insects {cattle, animals}	South America, Africa	5	7	***	0.355 (0.195, 0.515)	0·082	11.6
*Trypanosoma tungarae*	Dixenous	Anura	Non-insects {frogs}	South America	1	1	NA	NA	NA	NA
*Trypanosoma vegrandis*	Dixenous	Chiroptera and others	Non-insects {bats, animals}	Australia	3	3	*	0.627 (0.355, 0.899)	0·139	34.18
*Trypanosoma vivax*	Dixenous	Diptera, Artiodactyla, Perissodactyla and others	Insects {flies}, Non-insects {cattle, sheep, goats, camels, dogs, other animals}	Africa, South America	63	82	***	0.307 (0.257, 0.357)	0·026	8.61
*Trypanosomatidae* spp.	Mixed	Diptera, Hemiptera, Siphonaptera, Chiroptera and others	Insects {true bugs, fleas, flies}, Non-insects {reptiles, bats, rodents}	Europe, South America, Asia and Africa	14	18	***	0.468 (0.356, 0.584)	0·20	20.0
*Trypanozoon*	Dixenous	Diptera, Artiodactyla and others	Insects {flies}, Non-insects {animals, pigs, camels, dogs, cattle}	Africa	6	9	***	0.356 (0.212, 0.50)	0·074	11.7
*Endotrypanum* spp.	Dixenous	Diptera	Insects {flies}	South America	2	2		0.212 (−0.0756, 0.499)	0.1466	3.83

Parasite groups of the included studies. Parasite groups ending with ‘spp’. were unable to be identified to the species level. Systematic and quantitative summary of the main features are outlined for each group.

*P < 0.05, **P < 0.01, ***P < 0.001.

**Figure 3.**
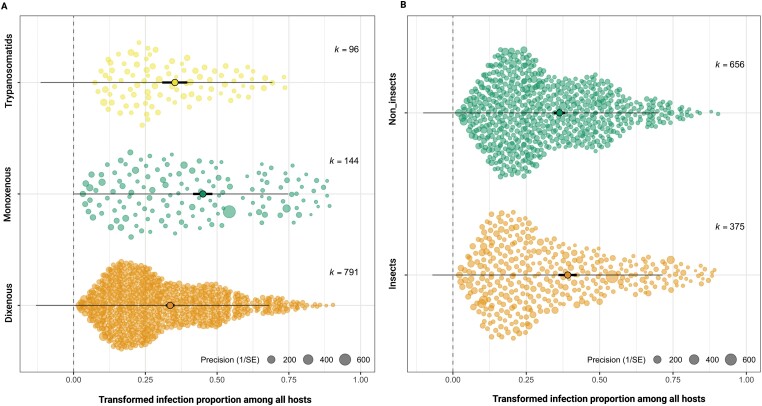
Orchard plots of infection prevalence among trypanosomatids that vary according to the number of hosts they infect (A) and whether those hosts are insects or non-insects (B). The X-axis shows effect sizes for each study (circles). Random jitter was added on the Y-axis to aid visibility. Freeman–Tukey double arcsine transformed pooled effect size (proportion of infection) is shown in the middle accompanied with bold lines representing the 95% CI. (A) Pooled estimate of infection prevalence of dixenous (0.381, 95% CI:0.360, 0.40), monoxenous (0.4867, 95% CI:0.413, 0.560), and mixed trypanosomatids (0.391, 95% CI:0.340, 0.442) with significant difference between dixenous and monoxenous (*P* = 0.0089, Benjamini–Hochberg adjusted cut-off *P* = 0.016). These pooled estimates back-transform to 13.3% (dixenous), 21.5% (monoxenous) and 14.0% (mixed trypanosomatids). (B) There is no overall significant difference between the pooled estimate of infection prevalence of insect (0.413, 95% CI: 0.377, 0.450) and non-insect (0.380, 95% CI:0.359, 0.402) hosts (*P* > 0.016; Benjamini–Hochberg adjusted cut-off). These pooled estimates correspond to 15.6% and 13.3% infection prevalence.

**Figure 4.**
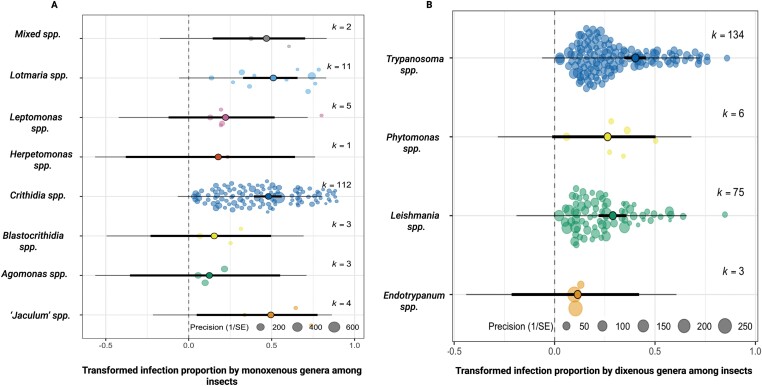
Orchard plots of monoxenous (A) and dixenous (B) parasites prevalence in insect hosts. The X-axis shows effect sizes for each study (circles). Random jitter was added on the Y-axis to aid visibility. Freeman–Tukey double arcsine transformed pooled effect size (proportion of infection) is shown in the middle accompanied with bold lines representing the 95% CI. (A) Pooled estimate of infection prevalence of monoxenous genera, representing a pooled estimate of 0.525 (CI: −0.084, 0.965), 0.599 (CI: 0.484, 0.713), 0.240 (CI: −0.167, 0.647), 0.215 (CI: −0.392, 0.821), 0.519 (CI:0.417, 0.621), 0.162 (−0.246, 0.570), 0.125 (CI: −0.455, 0.705) and 0.576 (CI: 0.0199, 1.132) for the monoxenous species, *Lotmaria, Leptomonas, Herpetomonas, Crithidia, Blastocrithidia, Angomonas* and ‘jaculum’ species, respectively. The infection prevalence did not differ significantly among monoxenous species of trypanosomatids. These pooled estimates correspond to a prevalence of 24.1%, 31.0%, 3.94%, 2.82%, 23.5%, 0.994%, 0.185% and 28.8%. (B) Pooled estimates of infection prevalence in dixenous genera, of 0.116 (CI: 0.217, 0.449), 0.298 (CI: 0.223, 0.374), 0.270 (CI: −0.0121, 0.553) and 0.426 (CI: 0.362, 0.490) for *Endotrypanum, Leishmania, Phytomonas* and *Trypanosoma* corresponding to prevalence of 0.948%, 8.30%, 6.78% and 16.8%. The infection prevalence did not differ significantly among dixenous species of trypanosomatids (*P* >adjusted cut-off based on Benjamini−Hochberg correction).

One of the main aims of this work was to quantitatively examine, for the first time, the impact of life-history traits on infection prevalence. Thus, we sat to test whether pooled prevalence of monoxenous infections differ from dixenous infections (both among insect and non-insect hosts). We found a significant difference (*P* = 0.0067) between pooled prevalence of monoxenous (20.6%) and dixenous infections (13.2%) (Fig. 3A). Introducing ‘host-traits’ as a sole moderator (i.e. insects and non-insects) reveals no significant difference (after correcting for multiple testing) between pooled prevalence of the two groups, 15% and 13.3%, respectively (Fig. 3B). Limiting the analysis to insects alone revealed a similar trend where monoxenous infections are approximately two-fold more prevalent (21.9%) than dixenous infections (11.1%) (Fig. 4, Table 2). We then compared prevalence in the groups of insects that host both dixenous and monoxenous parasites (true bugs and flies). Here, however, we found non-significantly higher pooled prevalence for dixenous infections compared to monoxenous infections in flies, and similar pooled prevalence for dixenous and monoxenous infections in true bugs (Table 2).

**Table 2.** Meta-regressions on subgroups of interest

Meta-regressions ID	Host grouping	*K*	Pooled estimate for group 1 (CI·lb, CI·ub)	Microscopic (%)	Pooled estimate for group 2 (CI·lb, CI·ub)	Molecular (%)	(*t*-test)	Sig	Adjusted cut-off	I2
F1	Insects	359	0.455 (0.399, 0.510)	19.1	0.389 (0.342, 0.436)	14.1	0.0013	***	0.008	99.55
F2	Non-insects	656	0.277 (0.236, 0.318)	7.14	0.375 (0.351, 0.399)	13.1	<0.0001	***	0.002	99.25
	**Host grouping**	***K***	**Pooled estimate for group 1 (CI·lb, CI·ub)**	**Dixenous** **(%)**	**Pooled estimate for group 2** **(CI·lb, CI·ub)**	**Monoxenous (%)**	**(*t*-test)**	**Sig**	**Adjusted cut-off**	**I2**
F3	Insects	350	0.372 (0.322, 0.422)	12.34	0.497 (0.416, 0.578)	22.1	0.0097	**	0.014	99.5
F4	Flies	186	0.297 (0.245 0.347)	8.24	0.219 (−0.013, 0.424)	4.37	0.457			98.9
F5	True bugs	48	0.513 (0.423, 0.605)	23.7	0.482 (0.250, 0.713)	21.0	0.788			99.2
	**Parasite grouping** ^a^	***K***	**Pooled estimate for group 1 (CI·lb, CI·ub)**	**Insects** **(%)**	**Pooled estimate for group 2** **(CI·lb, CI·ub)**	**Non-insects (%)**	**(*t*-test)**	**Sig**	**Adjusted cut-off**	**I2**
*F6*	*Dixenous spp*	819	0.357 (0.334, 0.380)	11.9	0.378 (0.358, 0.398)	13.3	0.0067	**	0.01	99.28
*F7*	*Leishmania spp·*	298	0.332 (0.294, 0.369)	10.4	0.394 (0.367, 0.421)	14·5	0.0001	***	0.006	99.2
*F8*	*Trypanosoma spp·* ^b^	427	0.326 (0.292, 0.360)	10.0	0.337 (0.306, 0.367)	10.7	0.241			99.4
*F9*	*Trypanosoma cruzi*	94	0.442 (0.373, 0.510)	18.0	0.469 (0.403, 0.529)	20.2	0.340			99.2
	**Bee Groups**	***K***	**Pooled estimate for group 1 (CI·lb, CI·ub)**	**Wild** **(%)**	**Pooled estimate for group 2** **(CI·lb, CI·ub)**	**Managed (%)**	**(*t*-test)**	**Sig**	**Adjusted cut-off**	**I2**
F10	All	123	0.554 (0.440, 0.668)	26.8	0.505 (0.207, 0.804)	22.2	0.75			99.7
F11	Honeybee	17	0.631 (0.448, 0.815)	34.2	0.481 (0.082, 0.880)	20.1	0.50			99.8
F12	Bumblebee	106	0.530 (0.398, 0.663)	24.4	0.527 (0.113, 0.941)	24.2	0.99			99.2

^a^Only dixenous species with prevalence data from both insects and non-insects.

^b^Prevalence data for *Trypanosoma cruzi* are not included here.

All of the analysis used meta-regressions with random effect model and an appropriate nested random design.

K is the total prevalence data included in the analysis in each row.

Adjusted cut-off of significance is based on Benjamini–Hochberg.

Given that most of the prevalence data for monoxenous species comes from bees, we wanted to ensure that sociality did not introduce a bias due to high within-colony prevalence when comparing between monoxenous and dixenous species (Supplementary File: Data extraction and processing). Thus, we re-extracted infection prevalence for bees by collapsing samples down to ‘colonies’ rather than ‘individuals’ and re-run all relevant meta-regressions (B.1 and F3). In cases where there was no information about infection prevalence at the colony level, we employed a highly stringent approach which assumes that any bee found in a single collection site is from the same colony. Such an approach should counter any inflated infection prevalence estimates due to high prevalence within colonies. We accordingly updated the adjusted *P*-values for multiple testing (i.e. Supplementary Table 4). We found similar results for both analyses, demonstrating that the results hold when colony level prevalence is analyzed rather than the number of infected individual bees (i.e. meta-regressions B.1 and F3).

Notably, dixenous parasites infect 1–17% of their insect hosts with no significant difference among parasite genera (Fig. 4B). Common dixenous parasites, with prevalence data from both insects and non-insects, were slightly but significantly more prevalent in their non-insect hosts (13.3%) compared to their insect hosts (11.9%) (Table 2).

Monoxenous genera exhibit two alternative trends: high pooled prevalence for *Lotmaria*, jaculum and *Crithidia* species, but low pooled prevalence for *Herpetomonas, Leptomonas, Agomonas* and *Blastocrithidia* groups (Fig. 4B). Taking into consideration that much of the monoxenous data (80%) were on bee infections, we performed a meta-regression on subgroups to further understand the main drivers of bee infection prevalence. While the majority of included data represent natural infection among wild bees, the remaining evidence describes spillover of infections e.g. by sampling non-native/commercial bees. We did not find any difference in infection prevalence between wild-bees and commercial/managed bees for bumblebees or honeybees (Table 2).

Honeybee infections seem to predominantly occur in Western/European honeybees (i.e. *Apis mellifera* Linnaeus) compared to Asian honeybees (e.g. *Apis dorsata* Fabricius and *Apis cerana* Fabricius). We also noted a significant reduction in heterogeneity of evidence across all honeybee-infections due to spatial variation only (46% heterogeneity). However, a much higher diversity of infection prevalence was found in bumblebee taxa. Accounting for spatial and temporal factors failed to significantly reduce the heterogeneity in evidence for bumblebee infections, suggesting a unique impact of various bumblebee-trypanosomatids systems on different patterns of prevalence (Fig. 3B) and likely the greater diversity of bumblebees.

Diagnostic approach also affected infection prevalence but differently according to host. Microscopic diagnostics revealed higher infection prevalence in insects (191%) whereas molecular assays report higher infection prevalence (13.1%) in non-insects (Table 2): suggesting a preferred, or necessary, methodology of researchers working with insects versus non-insects hosts.

## DISCUSSION

Trypanosomatid parasites have two distinct life histories that vary in the number of host species necessary to complete their life cycle. This difference is profound and may have commensurately profound effects on the evolution of parasite traits, including their ability to establish infection. How these alternative life-history strategies influence infection prevalence, however, is poorly understood. To our knowledge, this is the first comprehensive study designed to test for differences in infection prevalence.

We found that monoxenous trypanosomatids are consistently more prevalent, by nearly two-fold, than dixenous species in insects alone (Table 2) and similarly across all hosts (Fig. 3A). Taken together, one cannot escape the impression that complex parasite life-cycles have a cost to infection prevalence. In line with such speculation, high prevalence for monoxenous species was mainly notable among genera that infects bees such as *Crithidia* and *Lotmaria* with pooled prevalence of 23.9% and 24.9%, respectively; ranking bees with the highest pooled prevalence among all insects (27.0%) (Supplementary Fig. 5). It is striking to realize that about a quarter of surveyed bees are infected by trypanosomatid pathogens. This is of particular concern considering the crucial role of bees as pollinators, which, with other insect pollinators, account for the fertilization of 83% of flowering plants [30] including nutritionally and economically important crop species. Bees and other pollinators have been declining. Among the presumed causes of pollinator declines are infectious diseases [31]. Despite the attention that bee-trypanosomatid infection has attracted, the distribution of interest remains uneven. Infection prevalence of trypanosomatids is largely unexplored outside of Europe and North America (Supplementary Fig. 5). The relationship between wild and managed bees, and the risk of spillover in either direction, has raised considerable concern. While managed bees have been blamed for parasite transmission to wild bees [32], we found no significant difference between managed and wild honeybees and bumblebees (either independently or combined) in infection prevalence. Whether this reflects shared susceptibility to infection or ongoing spillover to and from wild pollinators remains unknown. Data about trypanosomatid infection prevalence in solitary bees is scant. However, recent studies have found that *C. bombi* and *C. mellificae* are both able to infect and replicate in solitary bees, with high infection prevalence [33, 34], suggesting that solitary bees are host to trypanosomatids parasites. Thus, future studies on infection prevalence of monoxenous trypanosomatids in solitary bees might help disentangle the impact of host social structure on parasite prevalence.

To our knowledge, this is the first study to provide a full summary of prevalence data among all host-trypanosomatids systems. Encouragingly, our findings recapitulate other taxon- or geographically restricted systematic reviews. For instance, a similar range of prevalence (2.4–9.2%) for trypanosomatids was reported in sand-flies (*Phlebotomus* spp.) in Iran [35], which is comparable to our finding of 10.14% (Table 2, meta-regression F7). Another meta-analysis on trypanosomatids in tsetse flies revealed a prevalence of 10%, as did we in flies infected by *Trypanosoma* [36]. Thus, although biting flies are responsible for most human trypanosomatids, our results seem to comport with previous studies revealing low infection prevalence in flies as a group, with no significant difference between monoxenous and dixenous parasites within flies (Table 2). Meanwhile, pooled prevalence for trypanosomiasis in cattle, sheep and goats was reported as ranging between 1% and 9%, which is comparable to what we find (meta-regression F8 in Table 2, and Supplementary Fig. 7) [37–39].

We also reveal significantly higher prevalence in the vertebrate hosts than vector flies, consistently across genera (Table 2). This brings to the fore a possibility that dixenous trypanosomatids infect more of their vertebrate hosts than their insects vectors, seemingly similar to other dixenous pathogens [16–19]. Evolutionary theories predict a life history trade-off between investment in survival (within-host persistence and growth) and reproduction (between-host transmission). For *Trypanosoma brucei* and other dixenous trypanosomatids; such relationship is amplified by the number of host species needed to complete the life-cycle [40]. While it was a widely held view that dixenous trypanosomatids are less virulent in their vectors than their non-vector hosts, this has been called into question recently. Dixenous trypanosomatids not only alter the feeding behaviour of vectors [41] but also reduce survival and life expectancy of infected insect vectors [42]. Virulence can also be exacerbated when vectors are infected by multiple species or genotypes of parasites [43]. These studies suggest that virulence in vectors may act as a selection force that lowers infection prevalence in vectors. This trend warrants further investigation, as understanding how these species are maintained in the multi-host metapopulation holds great potential in managing the spread of vector-borne diseases. For instance, if the prevalence of infection in vectors is low, further reduction in insect hosts may better control infection in the metapopulation than suppression in vertebrate hosts.

In this study, we found that microscopic and culture-based diagnostics revealed higher infection prevalence in insects but greater infection prevalence was revealed from molecular assays in non-insects (Table 2). This result does not necessarily indicate greater accuracy for either assay, but may rather indicate the preferred methodology of researchers working with insect versus vertebrate hosts. In line with this explanation, Abdi and researchers [36] concluded that microscopic diagnostics were more common in surveys of tsetse flies than molecular assays and Aregawi *et al* [44]. reported a higher range of prevalence (6–28%) by molecular tests in vertebrates compared to microscopic tests (2–9%) for the detection of *Trypanosoma evansi*. This preference may actually reflect practical or ethical necessity rather than methodological predilection. For example, sampling an entire animal is entirely standard for the microscopic screening of a fly, but sampling a whole ungulate is an entirely different matter. Recognizing differences in the diagnostic method applied for the detection of trypanosomatids within insects and non-insects is a crucial consideration for future research to enable accurate comparisons of prevalence. To control for this effect, we included this factor in all meta-regressions (either on subgroups or the full dataset) to ensure that the difference in prevalence accounts for variable diagnostics. To our assurance, in all of the main subgroup analysis, there was no significant difference in pooled prevalence due to different diagnostic tools when examining dixenous trypanosomatids among insects and non-insects, and individual groups of vertebrate hosts (e.g. cats, dogs, rodents, camels, goats, pigs, bats and buffaloes).

The key takeaway from this study is the unique pattern of infection prevalence in association with different life-history traits of trypanosomatids: revealing both (i) higher pooled prevalence for monoxenous species in insects and across all hosts, and (ii) consistently significant lower prevalence among vectors compared to subsequent hosts for dixenous species. We present a number of non-mutually exclusive arguments that could explain these findings:

- First, monoxenous species are subject to selection pressure from only one host, thereby affording these species more opportunity to adapt to this host than parasites that must co-evolve with both an insect vector and an additional, often vertebrate, host. We would thus expect to see greater parasite fitness in specialised monoxenous parasites than in dixenous parasites as the monoxenous parasites would arrive closer to their evolutionary optima. Dixenous parasites, in contrast, may be forced to trade-off infection relevant traits in one host for traits in the second host [22].
- Second, these findings might be due to an equal force of infection shared among differing numbers of host species. Here, we offer a numerical explanation, where the same hypothetical number of infectious units are simply shared among more host species in dixenous parasites, thus diluting the infection prevalence in any one host species—to approximately half. This explanation is more than passingly similar to the ‘dilution effect’ which is often, and sometimes controversially, used to describe infection risk in human populations when the natural host is not usually the human—e.g. Lyme disease [45], although to our knowledge it has not been invoked to explain variation in infection prevalence among vectored and non-vectored parasites.
- Third, host characteristics such as lifespan, recruitment rate, sociality, and physiology may explain some of the variation in infection prevalence. For instance, social bees, which have higher infection prevalence, differ from the rest of the insect hosts not only in their sociality but also in their lifespan. The biological unit for social bees is the colony, which can extend the lifespan of an infection to at least a year (bumblebees) and to several years in honeybees. The other insect hosts live for up to a few months, and have high birth and death rates which, along with rarer direct interactions can suppress the overall prevalence of infection regardless of the parasite life-cycle strategy. Consistent with this view, we found low pooled prevalence among flies with no significant difference between monoxenous and dixenous species. Furthermore, flies might be physiologically harder to infect by trypanosomatids than bees as genomic studies have described a reduced immune repertoire in bees relative to dipterans [46].
- Fourth, lifespan may also explain the higher infection prevalence revealed in vertebrates compared to their vectors. Given that vertebrates live longer, they may encounter higher risk of being exposed to multiple infectious bites from vectors. Similarly, parasite diminished lifespan, i.e. virulence, may affect vectors and long lived hosts differently. If, for instance, virulence is appreciably higher in vectors we may see lower infection prevalence in vectors due to greater parasite induced mortality.
- Another possibility may have hidden the magnitude of infection prevalence in large mammals (versus insects) is the lower parasitemia for chronic infections. While we did not see any differences in infection prevalence between molecular and histological approaches in vertebrate screenings, it does raise the possibility of missing low titre cryptic chronic infections in vertebrates, especially when using microscopic diagnostic tools.
- Finally, while we excluded human trypanosomatid prevalence to avoid underestimating natural infection in the vertebrate component of dixenous life cycles due to medical intervention, we cannot exclude that existing management strategies could suppress infection prevalence of livestock hosts and insects (e.g. pesticides, sprays and bed nets). We do, however, naively assume that veterinary intervention is likely rarer than medical intervention would be, and so should bias these results less. In depth analysis of infection prevalence in managed and wild vertebrates, or disentangling the role of veterinary management in infection prevalence could help elucidate the consistency of this pattern of greater infection prevalence in vertebrate hosts relative to their vectors. Investigating the impact of various anthropogenic interventions on infection prevalence of both insects and non-insects is warranted in the future to fully understand the impact of the regional approaches in suppressing the transmission. For instance, although it seems that dixenous species have lower infection prevalence, several dixenous species alter their vector hosts’ feeding behaviour to feed on multiple hosts and thus increase transmission [41]. Vectors also appear to change behaviour in response to bed nets. Understanding the role of human intervention on prevalence would be important to examine more closely in more targeted studies.

Dixenous parasitism in trypanosomatids had evolved multiple times and millions of years ago. These lineages have clearly been successful, persevering for an estimated 100 million years in the Leishmaniinae [47]. Thus, despite the disadvantage imposed by the complexity of life-cycle that we describe here (i.e. reduced infection prevalence compared to their monoxenous kin), there must be other benefits to the dixenous life history that selected for the transition to, and the maintenance of, this complex life cycle. The tautological benefit of a dixenous life cycle, is having two hosts. Having multiple hosts allows several potential benefits. The greater division between investment in growth (e.g. vertebrate host) and sexual reproduction (largely in insect vector host) [48, 49] may optimize the processes of both. This, for instance, allows greater clonal amplification in resource-rich large-bodied hosts and uses abundant highly mobile vectors for transmission. Maintaining infection in a long-lived host may also buffer parasites from the boom-and-bust population dynamics, seasonality and high extrinsic mortality rates of insect hosts. Finally, transmitting to many vectors may allow occasional co-infection with other genotypes of parasite, and thus outcrossing potential.

It is worth noting that variation in transmission route has been implicated in other key parasite traits, most notably, in virulence. Ewald’s landmark paper [50] proposed that vectored parasites are freed from the trade-off between transmission and virulence. Support for this attractively intuitive hypothesis has been both theoretically and empirically mixed [51, 52]. While we did not examine virulence here, we were able to avoid one criticism of Ewald’s original work [53] by comparing only among closely related parasites and including both host and parasite as random effects we can escape being phylogenetically confounded. We found distinct differences in infection prevalence depending on transmission route, demonstrating that broad scale parasite features, such as prevalence, or virulence, can be detected with such approaches.

We speculate that these findings may provide direction for potential trypanosomatid interventions. By identifying lower prevalence in the insect vector of dixenous trypanosomatids we may also have identified the weaker link in transmission; where any intervention that further suppresses infection, may have out-sized effects on disease burden. This could take the form of reducing likelihood of vectors being infected via transmissible symbionts, as has been implemented in mosquito vectors of viral diseases [54]; suppression of vector population growth using transgenic-based approaches, delivery of drug to vertebrates that prevent establishment in or transmission to insect vectors [55] or preferential targeting of infected adult vectors for trapping efforts. Or any number of other interventions that elude the creativity and expertise of these authors. Modelling efforts to describe how differential suppression of infection prevalence in different components of dixenous parasites would be particularly informative.

While this study was ambitious in scope and exhaustive in design, there are limitations. Foremost is the comparatively small number of studies describing prevalence in monoxenous species. This is likely due to scarcity of publications for neglected trypanosomatids rather than study selection/handling approaches employed here. This study included all published reports of trypanosomatid systems, written in English, over the last 20 years that contribute to sylvatic transmission cycles, except studies that only examined human medical infections. Despite the exclusion of human infections, their impact and contribution to the transmission cycles either directly (to insects) or indirectly (to non-insects) have been accounted for by including studies surveying animals living around humans. This includes pets (dogs, cats), domesticated animals, and the vectors, such as true bugs (tritomines), which are particularly found in houses. Thus, although the direct interaction in humans-trypanosomatids systems falls beyond the scope of this study, what we show here (i.e. how trypanosomatids differ in their infection prevalence among various non-human hosts) is particularly important to enable effective prevention plans for neglected tropical diseases of humans and livestock. Understanding the fundamental processes that determine infection prevalence will help us better understand trypanosomatids in general and identifying ‘weak-links’ in the infection chain of vectored parasites, will enable better tailored control strategies for trypanosomatid diseases relevant to human, livestock and wildlife health as well as food security.

## Supplementary Material

eoad014_suppl_Supplementary_Figure_S1

eoad014_suppl_Supplementary_Figure_S2

eoad014_suppl_Supplementary_Figure_S3

eoad014_suppl_Supplementary_Figure_S4

eoad014_suppl_Supplementary_Figure_S5

eoad014_suppl_Supplementary_Figure_S6

eoad014_suppl_Supplementary_Figure_S7

eoad014_suppl_Supplementary_File

eoad014_suppl_Supplementary_Table_S1

eoad014_suppl_Supplementary_Table_S2

eoad014_suppl_Supplementary_Table_S3
