# Supplementary figures and images for: Double trouble: trypanosomatids with two hosts have lower infection prevalence than single host trypanosomatids
Source: Evol Med Public Health. 2023 May 16;11(1):202–18. doi: 10.1093/emph/eoad014 (PMC10317189; doi:10.1093/emph/eoad014)

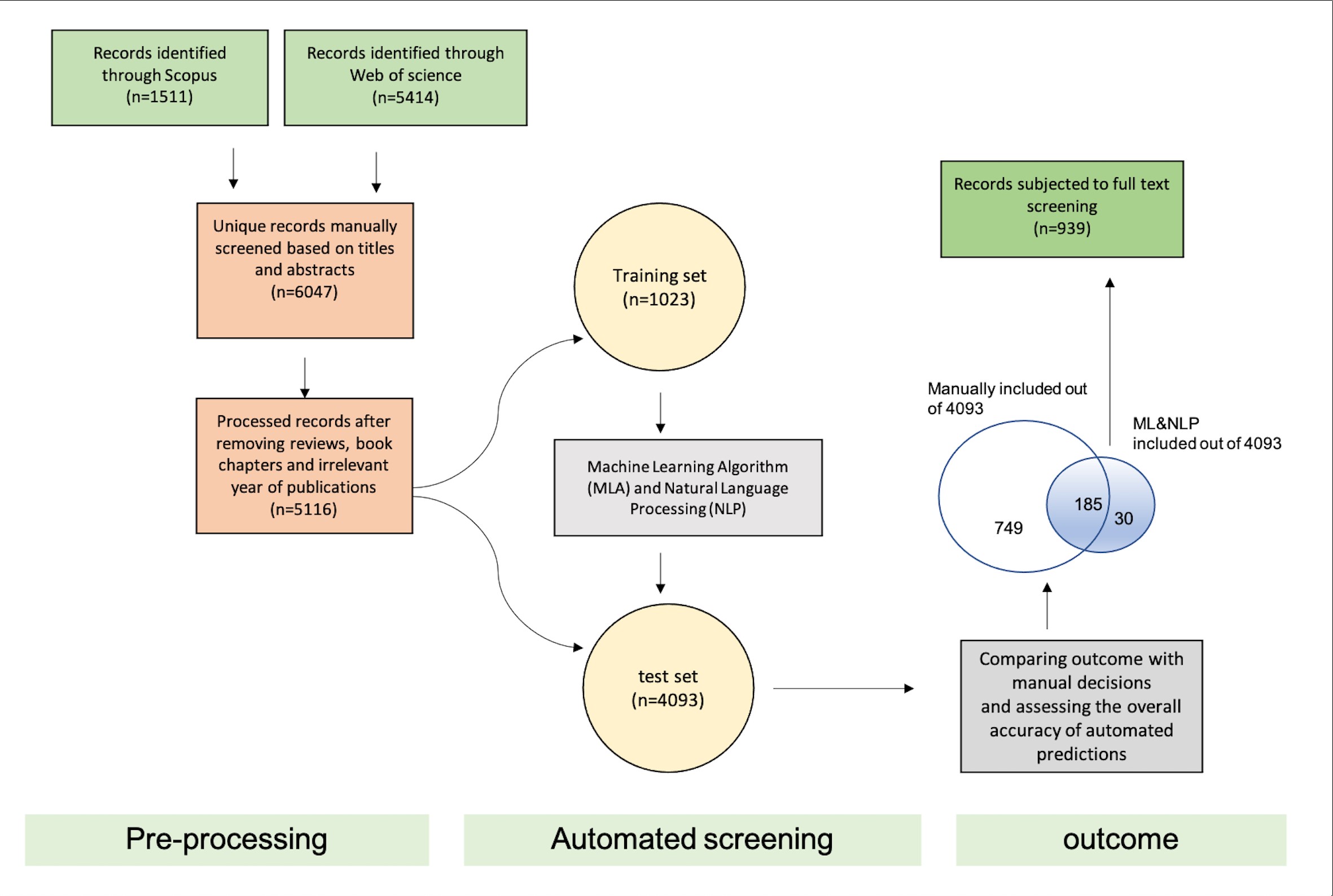

Supplement: eoad014_suppl_Supplementary_Figure_S1 [file eoad014_suppl_supplementary_figure_s1.jpeg]

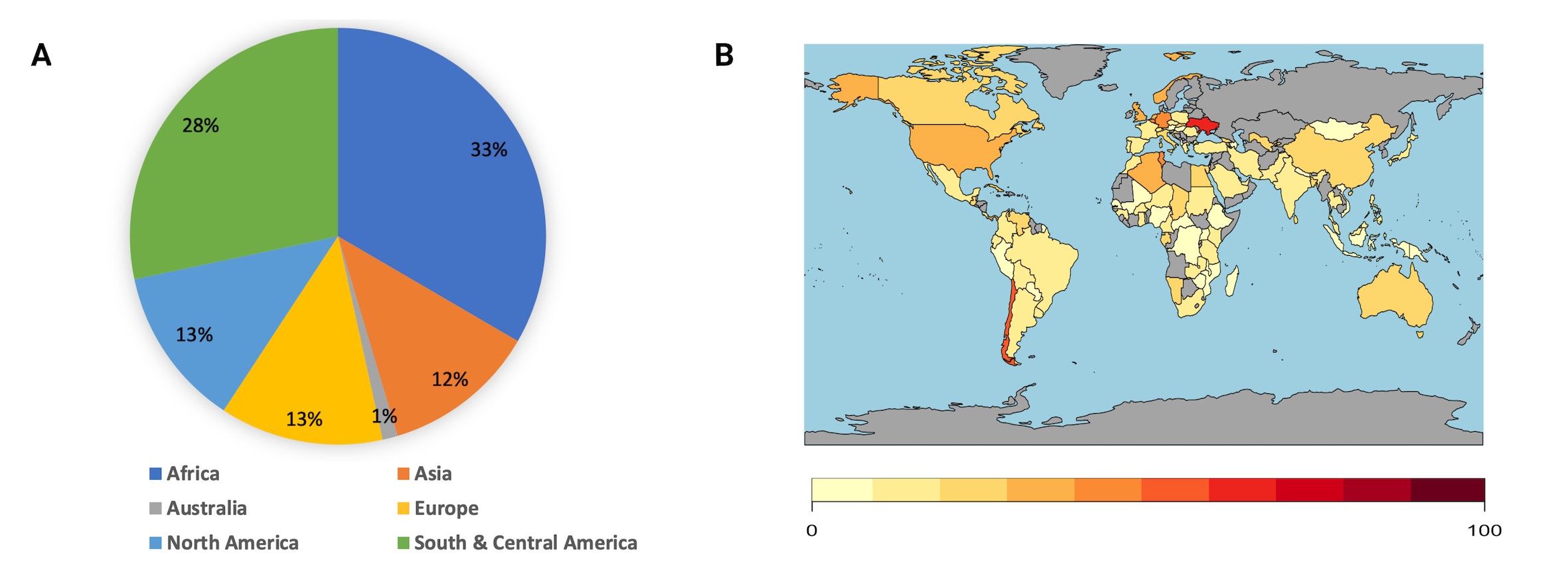

Supplement: eoad014_suppl_Supplementary_Figure_S2 [file eoad014_suppl_supplementary_figure_s2.jpeg]

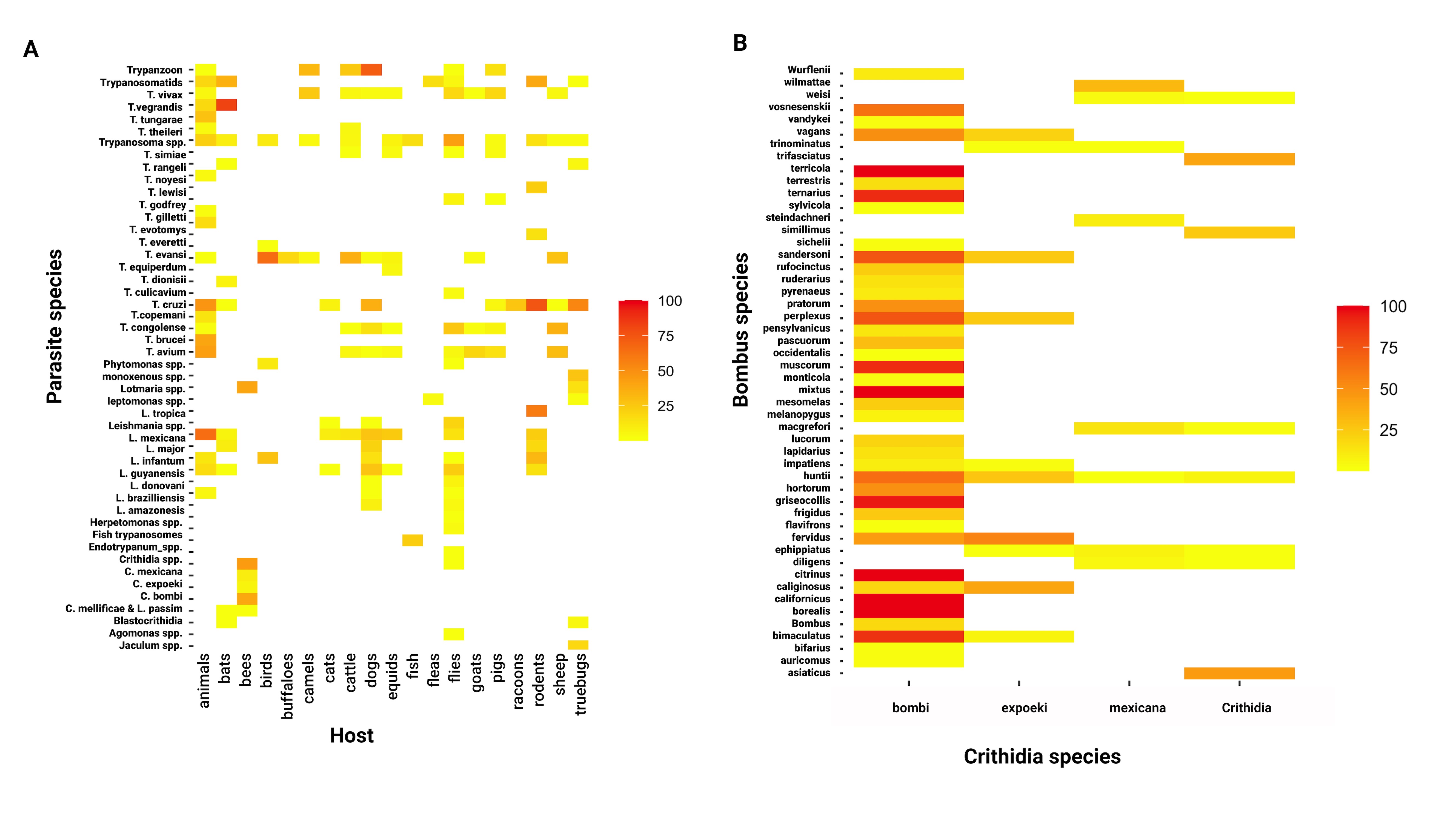

Supplement: eoad014_suppl_Supplementary_Figure_S3 [file eoad014_suppl_supplementary_figure_s3.jpeg]

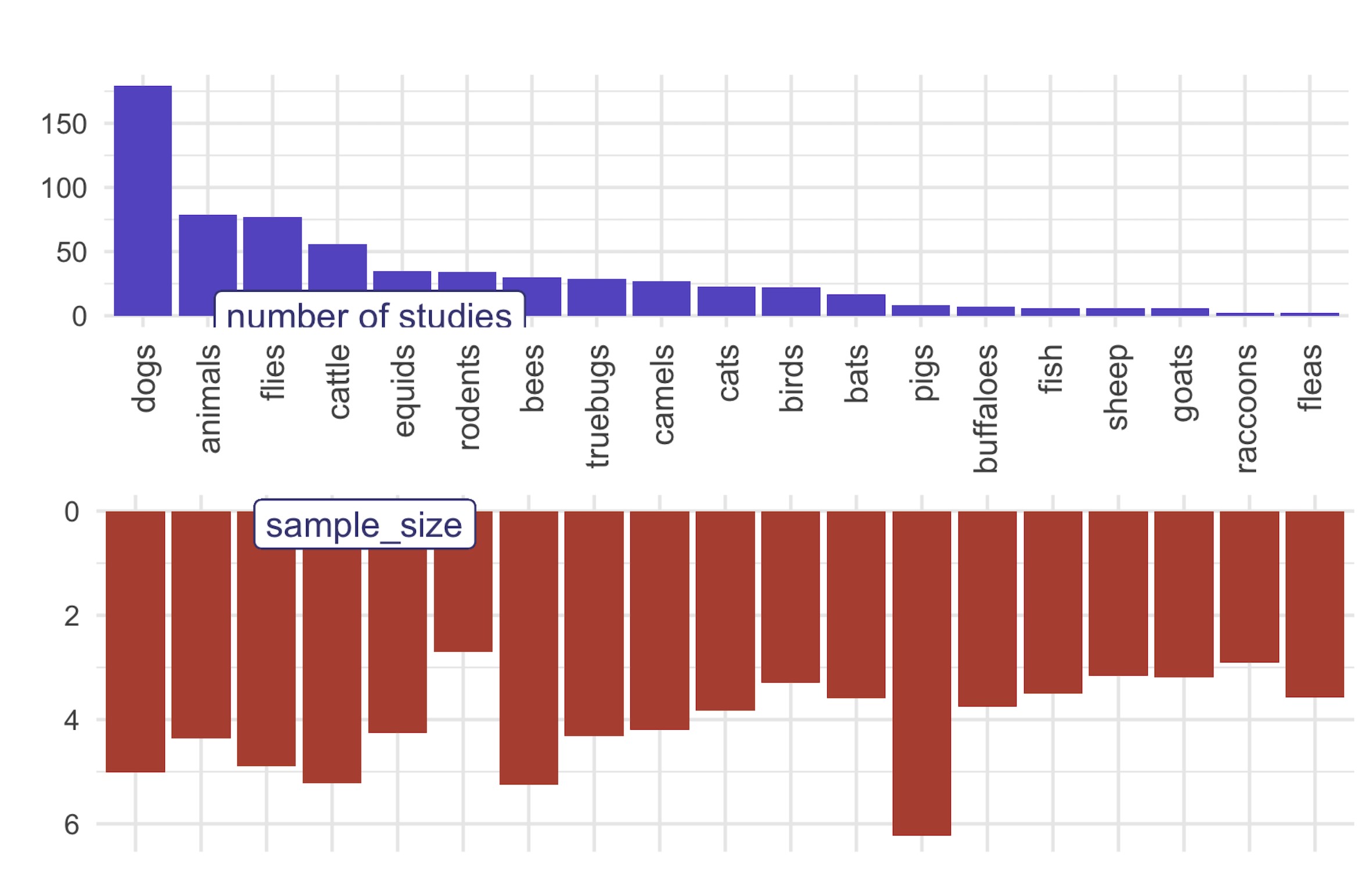

Supplement: eoad014_suppl_Supplementary_Figure_S4 [file eoad014_suppl_supplementary_figure_s4.jpeg]

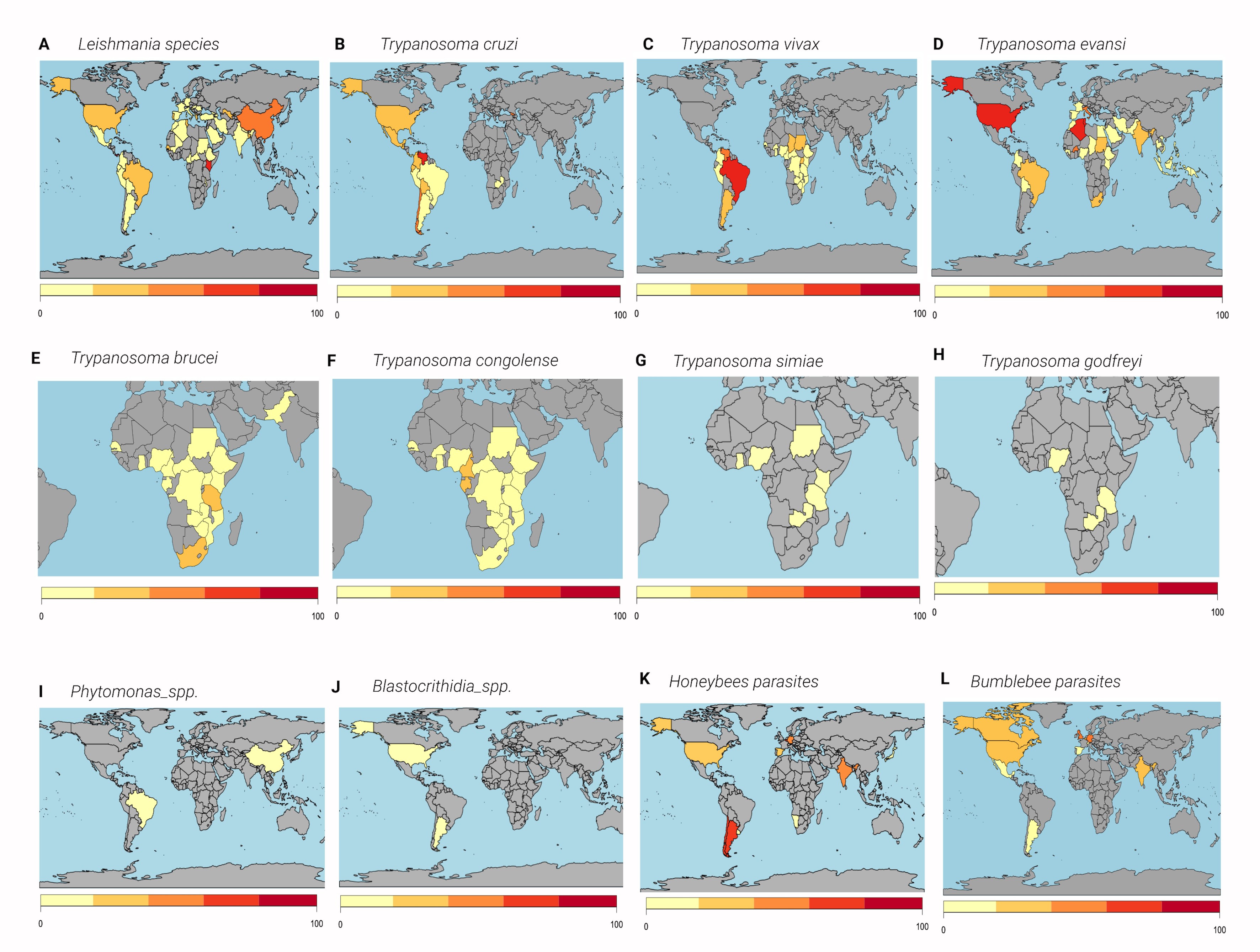

Supplement: eoad014_suppl_Supplementary_Figure_S5 [file eoad014_suppl_supplementary_figure_s5.jpeg]

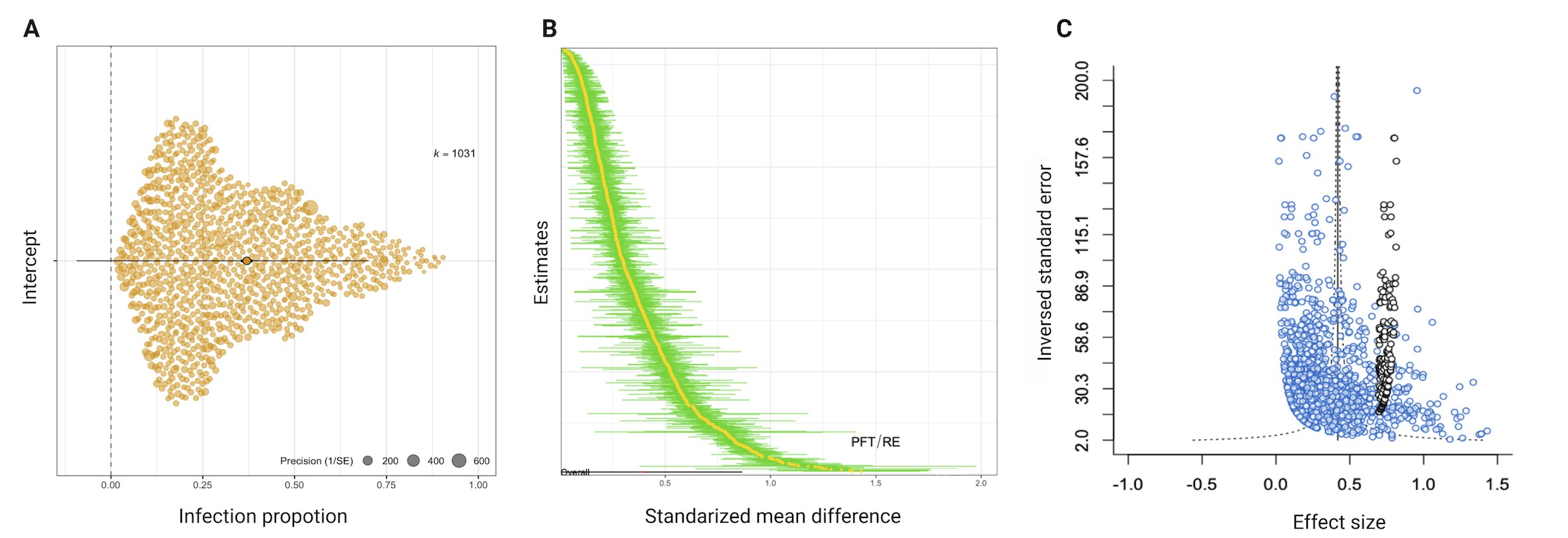

Supplement: eoad014_suppl_Supplementary_Figure_S6 [file eoad014_suppl_supplementary_figure_s6.jpeg]

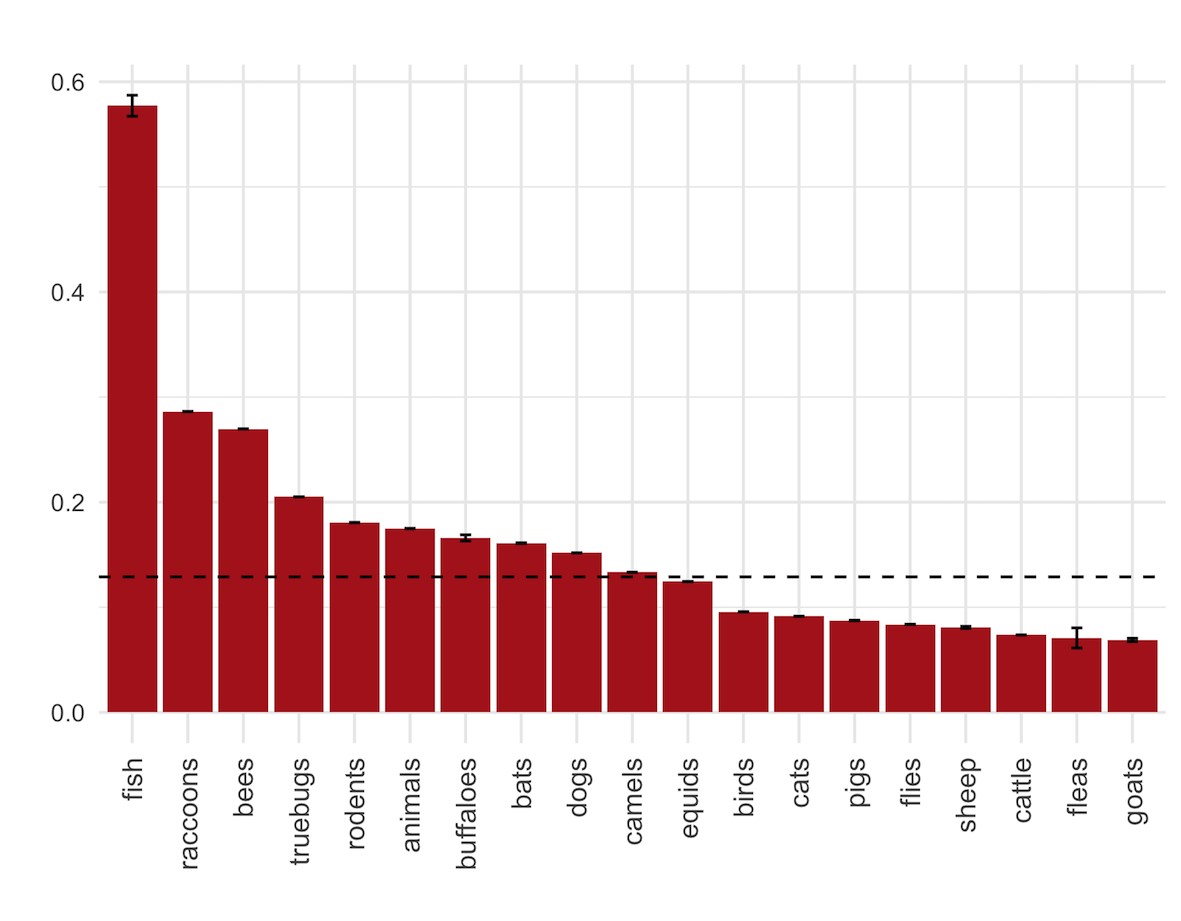

Supplement: eoad014_suppl_Supplementary_Figure_S7 [file eoad014_suppl_supplementary_figure_s7.jpeg]
