# Supplementary material for: Double trouble: trypanosomatids with two hosts have lower infection prevalence than single host trypanosomatids
Source: Evol Med Public Health. 2023 May 16;11(1):202–18. doi: 10.1093/emph/eoad014 (PMC10317189; doi:10.1093/emph/eoad014)
Supplement: eoad014_suppl_Supplementary_File [file eoad014_suppl_supplementary_file.docx]

# Supplementary material

# Extended protocol

## Inclusion and exclusion criteria

A publication was provisionally included if it (I) was published in English between 2000 to 2020 with cross-sectional or prospective (longitudinal) design, (II) contains data on any positive diagnostic test for trypanosomatids in any naturally infected non-human host, (III) contains all necessary information to assess prevalence or enough information to calculate prevalence independently, (IV) describes the diagnostic test used.

Conversely, a publication was excluded if it failed to meet the above criteria or reports data derived from secondary sources (e.g. publicly available databases or previously published data), (I) reports data on human infections or based on experimental infections only, (II) is not an original paper (e.g. reviews and letter to editors), (III) is based on clinical signs only with no diagnostic tests conducted to confirm prevalence, (IV) has insufficient information to calculate the prevalence independently or reports zero infection by a diagnostic test for trypanosomatids.

All hosts infected by single or multiple species were included , similarly, species infecting different hosts or single hosts were also included. Prevalence data was broken down to the taxa level of both the host and the parasite whenever its possible based on reported information.

## Manual screening based on titles and abstracts

A total of 6046 studies were entered to Rayyan website to initiate the manual screening process.^1^ Reviews, book-chapters, letter to editors, single case studies and papers reporting only human cases or experimental infections have been identified using a list of relevant keywords/phrases (e.g “experiment”, “experimentally infected”, “retrospective”, “review”, “meta-analysis”, “systematic review”, “in vitro”, “in vivo”, “drug”, “evolution”, “treatment”, “clinical trials”, “human”, “patients”, “individuals”, etc.). This generated a list of potentially irrelevant studies to be screened manually based on titles and abstract. A similar approach was employed to guide the initial screening of potentially relevant and eligible citations using keywords/phrases that include but not limited to the following: “survey”, “cross-sectional”, “prospective”, “natural infection”, “prevalence”, “occurrence” and “field study”. All studies then were subjected to full manual screening based on titles and abstracts, and no decision was made based on keyword calling only.

## Automated screening based on titles and abstracts

The primary dataset of citations, encompassing 6046 studies, was further processed before MLA-screening. This was done by removing all publications that would not necessarily be excluded by NLP and MLA alone, such as due to irrelevant year of publication, duplication and wrong study type (e.g reviews, single case studies, and letter to editor). The dataset was then split randomly into a training-set (20% of total dataset) and test-set (n=4093)(Supplementary Figure.[1](#suppfigure:Sup1)). The final outcome was compared with manual decisions, revealing an accuracy of 80%. All studies included by MLA and/or human reviewer were subjected to full-text review by two different reviewers (HA & SB) to ensure eligibility. The machine learning was done using "sklearn" package using "LogisticRegression model" and the code is available at <https://github.com/Hawra480/ML_updated1> and was run using python3.^2^

## Data extraction and processing

We extracted the main biological and geographical moderators (e.g “country”, “host type”, “parasite species” “year of publication”, “study type” and “study ID”) using partially automated python scripting, followed by full manual review of extracted data to ensure accuracy. The extraction of prevalence data (extracted as “total sample size” and “positive cases”) was manual extracted. Prevalence data was broken down to the taxa level of both the host and the parasite, with an average of 2-3 prevalence data extracted from each study. The exact host species was not available for all studies, thus, we conducted all relevant meta-regression models using the broad host categories (i.e. pigs, dogs, cats, camels, cattle, sheep, fish, raccoons, buffaloes, rodents, true bugs, flies, bees, birds, bats, fleas) and not the host-taxa level. Meta-regression models on bees, on the contrary, were conducted with the exact host taxa of bees-since it was available for all studies. Studies that report an overall prevalence data among several host categories without enough information to further break it down to each host level, were grouped under “animals". This group also includes host taxa found in single studies (such as frogs) as meta-regression models would otherwise be meaningless for that taxa. Sample size was standardised to the number of “individuals" sampled. Therefore, prevalence data among flies and bees, which are usually reported either as number of “pools” and number of “colonies” receptively, was estimated to “individual" number based on information reported to reflect the reported prevalence in each study. To make sure that eusociallty did not introduce a bias due to within-colony prevalence, we re-extracted infection prevalence among bees using number of "colonies" rather than "individuals" and re-run all relevant meta-regressions. Studies that do not outline the number of colonies and only report the number of bees examined were normalised by utilising the prevalence per geographic site, with all bees collected from one geographic site being assumed to belong to one colony to be extraordinarily conservative. We revealed similar significance for both data extraction methods of bees while comparing between monoxenous and dixenous infections among insects only and all hosts (meta-regressions B.1 and F3), and the p-values were reordered from lowest to highest to account for multiple tastings (Supplementary Table 4). A phylogenetic representation of host taxa was created using "rotl" and "ggtreeExtra" packages^3–5^ in R.^6^

## Source data for qualitative and quantitative assessments in the main manuscript and supplementary figures

met-regression A (Table-1), met-regression B(Figure 3A), meta-regression C (figure 3B), meta-regression G (supplementary Figure 8) and the qualitative assessment for the geographic burden of trypanosomatid infection (supplementary Figure 3 and 5) were all conducted using the full data-set (n=584, K=1031).^7–588^ The remaining meta-regressions were conducted on subgroups: meta-regression D (n=584, K=1031),^22,40,43,51,58,60,64,69,91,99,105–107,119,132,134,150,153,172,177,197,198,201,221,250–254,256,257,262,263,265,272,273^ meta-regression E,^9–11,19,26,27,37,46,53,54,72,76,83,89,93,94,97,100,103, 108,118,125,128,129, 137–140,146,149,152–154,158,159,171,176,179,180,187,189,192,205,206,208,213, 217,218,221,231, 235,246,248,249,255–257,260,261,267–270,290,298,301,309,317,320,324,327,334,344,347,349,361,368, 393,414,422,425, 429,444,446,456,467,476,477,483,487,498,500,508,514,517,529,533,539,547,548,568,584,586–589^ meta-regressions F1,^10,11,19,22,26,27,37,40,43,46,51,53,54,58,60,64,69,72,76,83,89,91,93,94,97,99,100,103,105–108,118,119,125,128,129,132,134, 137–140,146,149,150,152–154,158,159,171,172,176,177, 179,180,187,189,192,197,198,201,205,206,208,213,217, 218,221,231,235,246,248–257 ,260–263,265,267-270,272,273,290,298,301,309,317,320,324,327, 334,344,347, 349,361,368,393,414,422,425,429,444, 446,456,467, 476,477,483,487,498, 500,508,514,517,529,533,539,547,548,568,584,589^ meta-regression F2,^7,8,12–18,20,21,23–26,28,29,31–36,38,39,41,42,44,45,47–50,52,55–57,59,61–63,65–68,70,71,74,75,77–82,84–88,90,92,95,96,98,100–102,104,109–117,120–127,130,131,133,135,137,141–145,147,148,151,152,155,156,160–170,173–176,178,181–186,188,190,191,193–196,199,200,202–204,207,209–216,219,220,222–230,232–234,236–240,242–245,247,259,266,271,275–289,291–297,299,300,302–319, 321–323,325,326,328–332,335–342,345,346,348,350–360,362–367,369–392,394–413,415–421,423–428,430–443,445,447–455,457–466,468–475,478–486,488–497,499,501–506,508–513,515–528,530–532,534–538,540–546,548–562,564,566,567,569–583,585,590^ meta-regression F3,^10,11,19,22,26,27,37,40, 43,46,51,53,54,58,60,64,69,72,76,83,89,91,93,94,97,99,100,103,105–108,118,119,125,128,129,132,134,137–140,146,149,150,152–154,158,159,171,172,176,177,179,180,187,189,192,197,198,201,205,206,208,213,217,218,221,231,235,246,248–257,260–263,265,267–270,272,273,290,298,301,309,317,320,324,327, 334,344,347,349,361,368,393,414,422,425,429,444,446,456,467,476,477, 483,487,498,500,508,514,517,529,533,539,547,548,568,584,589^ meta-regression F4,^9–11,19,26,37,40,53,54,76,83,89,93,94,97,100,118, 125,128,129,137,138,149,152,154,159,176,179,180,187, 189,192,205,208, 213,217,221,231,235,246,248, 249,256,260,261,267–269, 290,298,309,334,347,361,368,422,425,429,446,456,467, 477,483,487,498,500,514,529,533,539,547,589^ meta-regression F5,^27,46,72,103,108,139,140,146,153,158,171,206,218,255–257,301,317,320,324,344,349,393,414,444,476,508,517,548,568,584^ meta-regression F6,^8–16,18–21,23–27,29,31–33,36–39,41,42,44–50,52–56,59,61–63,65–68,70–72,74–90,92–98,100–102,104,108,110–118,120–123,125,126,128–131,133,135,137–149,151–156,158–171,173–176,178–182,184–196,199,200,202–221,223–240,242–249,255,259–261,266–271,275,276,278,279,281–291,293–295,297–301,303–422,424–489,491–562,564,566–585,589,590^ meta-regression F7,^10–12,14,18–20,25,26,29,31,36,37,44,45,47–49,53–55,61,62,65,66,68,70,71,78,79,83–86,88–90,92,93,97,98,101,102,113,117,122,123, 126,128,129,135,137,141–143,147,154,156,164,167–169,186,189–191,193,194,196,203–205, 207,208,210,211,213,216,217, 219,232,238,246,248,249,261,267,275,276,278,279,281,283–285,287,290,291,294,295,298–300,306–310,312,313, 315,318,322,327–329, 331–334,336,339,341,343,345,347,348,350,353,355,357–363,365–368,371–373,375,376,378,379,383, 386,387,389,390,392,395,398,400–402,404,409,410,413,417,422,425,429,430,432–435,438–441,443,445,446,448,449,452–455,457–459,462,463,465–469,472,474,475,477,481,482,485,489,491,493,494,497–502,507,509–512,514,515,521,523,525,529,532,535–540,543,549,551,552,554,555,557–559,564,566,567,571,573–577,579,580,589,590^ meta-regression F8,^8,9,13,15,21,23,24,33,38,39,41,42,56,67,74–77,82,87,94–96,100,104,108,110–112,114,115,118,120,121,125,130,131,133,138,145,148,149,152,159–163,166,170,171,173–176,178–182,184, 185,187,188,192,195,200,202,209,214,215,220,221,223–231,233–237,239,240,242–245,247, 259,260, 266,268, 269,277,282,288,289,297,303–305,311,314,316,319,321, 326,330,337,338,340,342,349,354,356, 364,369, 370,377, 380,381,384,385,391,396,397,399,405–408,411,412,418–421,424,426–428,431,436,437,442, 447,450,451,456, 460,461,464,470,479,480,483,484,486–488,496,505,511–513,516,519,520,522,524,526, 527,530,531,533–535,541,542,544–547, 550,553, 560,561,572,578,581–583,585^ meta-regression F9,^16,27,32,36, 46,50,52,59,63,71, 72,80,81, 108,116, 139,140, 144,146,151,153,155,156,158,165,171,199,206,212,218,255,270,271,286,293,301,317,318,320,323–325,335, 344,346,351, 352,354,374,382,388,393,394,403,414–416,444,471,473,476,478,492 ,493,495 ,501, 503–506,508, 517,518, 528,548,556,562,568–570,579,584^meta-regression F10,^22,43,51,58,60,64,91,99,105–107,119, 132,134, 150,172, 177,197,198,201,250–254,262,263, 265,272,273^ meta-regression F11,^22,43,51,58,60,64,91,99,105–107,119, 132,134, 150,172, 177,197,198,201,250–254,262,263,265,272,273^ and meta-regression F12.^22,43,51,58,60,64,91,99,105–107,119, 132,134,150,172,177,197,198,201,250–254,262,263,265,272,273^

# Extended results

## General model and publication bias

Out of 6046 unique citations, we have included 584 studies. Preforming the meta-analysis for all trypanosomatids infections (k=1031) reveals a low overall pooled estimate of (0.367, Cls: 0.354, 0.385) back-transformed to a prevalence of (12.6%, Cls:11.5, 13.7), high heterogeneity rate (more than 90%) and no evidence of significant publication bias based on Egger’s regression test for funnel plot asymmetry(p=0.726) with the predictor being the sample size as recommended for ecological and evolutionary studies with high heterogeneities (Supplementary Fig.[6](#suppfigure:Sup6)A-C). We also run separate meta-regression models with each of the sole moderators to assess the overall impact on the mean effect size. As expected, all tested moderators showed significant influence (P$\leq$ 0.05) on the mean effect size, as per the Q_M_ statistics.

## Geographic burden of trypanosomatids infections

The majority of the infections (60%) were reported from the American continents. In contrast, infections in Africa, Asia and Europe each contribute 12-13% of the total infections. The remaining 1% of quantitatively included data were from trypanosomatids infections in Australasia (Supplementary Fig.[2](#suppfigure:Sup2)A).

Considering the huge diversity of trypanosomatids species, it is perhaps not surprising that most countries exhibit a low range of an overall prevalence (1-20%)(Supplementary Fig.[2](#suppfigure:Sup2)B), with the exception of some geographic hot spots for trypanosomatids infections. An example of these hot spots include Chile and Ukraine with an average prevalence of 50-70%. This is followed by a 40-50% prevalence in Belgium, Germany and Tunisia and lastly between 20-40% in Algeria, Norway, Netherlands, United Kingdom and United States.

Interestingly, these geographic hot spots are usually caused by 1-2 groups of trypanosomatids species (i.e. either due to concentrated reporting and/or high transmission dynamics in an ecological niche). For instance, high prevalences in Algeria is mainly caused by *Leishmania* and *Trypanosoma evansi* infections (Supplementary Fig.[5](#suppfigure:Sup5)A & Fig. [5](#suppfigure:Sup5)D). Similarly, high infection rates in Chile is mainly affected by honeybee parasites (i.e. *C. mellificae* and *L. passim*) and *Trypanosoma cruzi*(Supplementary Fig.[5](#suppfigure:Sup5)K & Supplementary Fig. [5](#suppfigure:Sup5)B). On the other hand, prevalence in India seems to be predominantly influenced by high prevalence of multiple Trypanosomatidae-groups: *Leishmania* species, *Trypanosoma evansi* and bee parasites (i.e. *C. mellificae*, *L. passim*, *C. bombi* and *C. expoeki*) (Fig.[5](#suppfigure:Sup5)A & Fig. [5](#suppfigure:Sup5)D & Fig. [5](#suppfigure:Sup5)K & Fig. [5](#suppfigure:Sup5)L ).

**References**

1. Ouzzani, M., Hammady, H., Fedorowicz, Z. & Elmagarmid, A. Rayyan—a web and mobile app for systematic reviews. *Systematic Reviews* **5**, 210 (2016).

2. Van Rossum, G. & Drake, F. L. *Python 3 reference manual*. (CreateSpace, 2009).

3. Michonneau, F., Brown, J. W. & Winter, D. J. rotl: An r package to interact with the open tree of life data. *Methods in Ecology and Evolution* **7**, 1476–1481 (2016).

4. Paradis, E. & Schliep, K. Ape 5.0: An environment for modern phylogenetics and evolutionary analyses in R. *Bioinformatics* **35**, 526–528 (2019).

5. Xu, S. *et al.* ggtreeExtra: Compact visualization of richly annotated phylogenetic data. (2021) doi:[10.21203/rs.3.rs-155672/v2](https://doi.org/10.21203/rs.3.rs-155672/v2).

6. R Core Team. *R: A language and environment for statistical computing*. (R Foundation for Statistical Computing, 2020).

7. Abebe, G., R. & Simon, I. Bovine trypanosomosis and vector density in omo-ghibe tsetse belt, south ethiopia. *Acta Trop* **167**, 79–85 (2017).

8. Adam, M., Y. & Van den Bossche, P. Bovine trypanosomosis in the upper west region of ghana: Entomological, parasitological and serological cross-sectional surveys. *Res Vet Sci* **92**, 462–8 (2012).

9. Adams, H., E. R. & Gibson, W. C. The identification, diversity and prevalence of trypanosomes in field caught tsetse in tanzania using ITS-1 primers and fluorescent fragment length barcoding. *Infect Genet Evol* **8**, 439–44 (2008).

10. Ajaoud, E.-S., M. & Lemrani, M. Phlebotomus sergenti in a cutaneous leishmaniasis focus in azilal province (high atlas, morocco): Molecular detection and genotyping of leishmania tropica, and feeding behavior. *PLoS Negl Trop Dis* **9**, e0003687 (2015).

11. Akhoundi, B., M. & Parvizi, P. Molecular characterization of leishmania infection from naturally infected sand flies caught in a focus of cutaneous leishmaniasis (eastern iran). *J Arthropod Borne Dis* **7**, 122–31 (2013).

12. Akhoundi, M., M. & Mirzaei, A. Molecular characterization of leishmania spp. In reservoir hosts in endemic foci of zoonotic cutaneous leishmaniasis in iran. *Folia Parasitol (Praha)* **60**, 218–24 (2013).

13. Alanazi, P., A. D. & Al-Shehri, H. R. Molecular detection of equine trypanosomiasis in the riyadh province of saudi arabia. *J Vet Diagn Invest* **30**, 942–945 (2018).

14. Alanazi, P., A. D. & Alraey, Y. A. Molecular detection of leishmania spp. In skin and blood of stray dogs from endemic areas of cutaneous leishmaniasis in saudi arabia. *Iran J Parasitol* **14**, 231–239 (2019).

15. Elsiddig, M., Mohamed, Y., Elshafie, E., Alharbi, Y. & Al-Mekhlafi, H. Molecular detection of trypanosoma evansi in camels (camelus dromedarius) in southwestern saudi arabia. *The Thai veterinary medicine* **49**, 93–100 (2019).

16. Aleman, G., A. & Hahn, D. The prevalence of trypanosoma cruzi, the causal agent of chagas disease, in texas rodent populations. *Ecohealth* **14**, 130–143 (2017).

17. Alias, S., S. N. & Mohd-Zain, S. N. Epidemiology of blood parasitic infections in the urban rat population in peninsular malaysia. *Trop Biomed* **31**, 230–40 (2014).

18. Amusategui, A. A., I.Sainz. Annals of the new york academy of sciences. in vol. 1026 154–157 (2004).

19. Anderson, S., J. M. & Kamhawi, S. Seasonality and prevalence of leishmania major infection in phlebotomus duboscqi neveu-lemaire from two neighboring villages in central mali. *PLoS Negl Trop Dis* **5**, e1139 (2011).

20. Cortes, S. *et al.* Risk factors for canine leishmaniasis in an endemic mediterranean region. *Veterinary Parasitology* **189**, 189–196 (2012).

21. Angwech, N., H. & Skilton, R. A. Heterogeneity in the prevalence and intensity of bovine trypanosomiasis in the districts of amuru and nwoya, northern uganda. *BMC Vet Res* **11**, 255 (2015).

22. Arismendi, B., N. & Vargas, M. PCR-specific detection of recently described lotmaria passim (trypanosomatidae) in chilean apiaries. *J Invertebr Pathol* **134**, 1–5 (2016).

23. Astudillo, H., V. G. & Yabsley, M. J. Spatial, temporal, molecular, and intraspecific differences of haemoparasite infection and relevant selected physiological parameters of wild birds in georgia, USA. *Int J Parasitol Parasites Wildl* **2**, 178–89 (2013).

24. Atarhouch, R., T. & Dakkak, A. Camel trypanosomosis in morocco 1: Results of a first epidemiological survey. *Vet Parasitol* **111**, 277–86 (2003).

25. Athanasiou, K., L. V. & Diakou, A. A cross-sectional sero-epidemiological study of canine leishmaniasis in greek mainland. *Acta Trop* **122**, 291–5 (2012).

26. Babuadze, A., G. & Imnadze, P. Epidemiology of visceral leishmaniasis in georgia. *PLoS Negl Trop Dis* **8**, e2725 (2014).

27. Bacigalupo, T.-P., A. & Cattan, P. E. Sylvatic foci of the chagas disease vector triatoma infestans in chile: Description of a new focus and challenges for control programs. *Mem Inst Oswaldo Cruz* **105**, 633–41 (2010).

28. Bajer, P., A. & Sinski, E. Factors affecting the component community structure of haemoparasites in bank voles (clethrionomys glareolus) from the mazury lake district region of poland. *Parasitology* **122 Pt 1**, 43–54 (2001).

29. Bamorovat, S., M. & Keyhani, A. Leishmania tropica in stray dogs in southeast iran. *Iran J Public Health* **44**, 1359–66 (2015).

30. Batistoti, C., M. & Jankevicius, S. I. Genetic variability of trypanosomatids isolated from phytophagous hemiptera defined by morphological, biochemical, and molecular taxonomic markers. *J Parasitol* **87**, 1335–41 (2001).

31. Belo, G., V. S. & Silva, E. S. da. Reliability of techniques used in the diagnosis of canine visceral leishmaniasis by the national control program in brazil: A survey in an area of recent transmission. *Prev Vet Med* **146**, 10–15 (2017).

32. Bento, G.-H., E. C. & Ramirez, L. E. Identification of bat trypanosomes from minas gerais state, brazil, based on 18S rDNA and cathepsin-l-like targets. *Parasitol Res* **117**, 737–746 (2018).

33. Berlin, N., D. & Baneth, G. Prevalence of trypanosoma evansi in horses in israel evaluated by serology and reverse dot blot. *Res Vet Sci* **93**, 1225–30 (2012).

34. Bernal, X. E. & Pinto, C. M. Sexual differences in prevalence of a new species of trypanosome infecting tungara frogs. *Int J Parasitol Parasites Wildl* **5**, 40–7 (2016).

35. Berzunza-Cruz, R.-M., M. & Becker, I. Leishmania (l.) Mexicana infected bats in mexico: Novel potential reservoirs. *PLoS Negl Trop Dis* **9**, e0003438 (2015).

36. Bezerra, C., C. M. & Diotaiut, L. Domestic, peridomestic and wild hosts in the transmission of trypanosoma cruzi in the caatinga area colonised by triatoma brasiliensis. *Mem Inst Oswaldo Cruz* **109**, 887–98 (2014).

37. Bhattarai, D., N. R. & Dujardin, J. C. Natural infection of phlebotomus argentipes with leishmania and other trypanosomatids in a visceral leishmaniasis endemic region of nepal. *Trans R Soc Trop Med Hyg* **103**, 1087–92 (2009).

38. Birhanu, F., H. & Buscher, P. Epidemiology of trypanosoma evansi and trypanosoma vivax in domestic animals from selected districts of tigray and afar regions, northern ethiopia. *Parasit Vectors* **8**, 212 (2015).

39. Biryomumaisho, R., S. & Lubega, G. W. Livestock trypanosomosis in uganda: Parasite heterogeneity and anaemia status of naturally infected cattle, goats and pigs. *Parasitol Res* **112**, 1443–50 (2013).

40. Borghesan, C., T. C. & Camargo, E. P. Genetic diversity and phylogenetic relationships of coevolving symbiont-harboring insect trypanosomatids, and their neotropical dispersal by invader african blowflies (calliphoridae). *Front Microbiol* **9**, 131 (2018).

41. Botero, T., A. & Thompson, R. C. Trypanosomes genetic diversity, polyparasitism and the population decline of the critically endangered australian marsupial, the brush tailed bettong or woylie (bettongia penicillata). *Int J Parasitol Parasites Wildl* **2**, 77–89 (2013).

42. Boushaki, A., D. & Kechemir Issad, N. Epidemiological investigations on trypanosoma evansi infection in dromedary camels in the south of algeria. *Heliyon* **5**, e02086 (2019).

43. Buendía, M.-H., Maria & Higes, M. Epidemiological study of honeybee pathogens in europe: The results of castilla-la mancha (spain). *Spanish Journal of Agricultural Research* **16**, (2018).

44. Can, D., H. & Guruz, Y. Seroprevalence of leishmania infection and molecular detection of leishmania tropica and leishmania infantum in stray cats of izmir, turkey. *Exp Parasitol* **167**, 109–14 (2016).

45. Cardia, C., D. F. & Bresciani, K. D. Prevalence of toxoplasma gondii and leishmania spp. Infection in cats from brazil. *Vet Parasitol* **197**, 634–7 (2013).

46. Cardinal, O., M. V. & Gurtler, R. E. Heterogeneities in the ecoepidemiology of trypanosoma cruzi infection in rural communities of the argentinean chaco. *Am J Trop Med Hyg* **90**, 1063–73 (2014).

47. Cardoso, R., L. & Schallig, H. D. Sero-epidemiological study of canine leishmania spp. Infection in the municipality of alijo (alto douro, portugal). *Vet Parasitol* **121**, 21–32 (2004).

48. Carvalho, W., F. S. & Rezende, R. P. Leishmania (viannia) braziliensis in dogs in brazil: Epidemiology, co-infection, and clinical aspects. *Genet Mol Res* **14**, 12062–73 (2015).

49. Cassini, S., R. & Pietrobelli, M. Preliminary study of the effects of preventive measures on the prevalence of canine leishmaniosis in a recently established focus in northern italy. *Vet Ital* **49**, 157–61 (2013).

50. Castillo-Neyra, C. C., R. & Levy, M. Z. The potential of canine sentinels for reemerging trypanosoma cruzi transmission. *Prev Vet Med* **120**, 349–56 (2015).

51. Cavigli, D., I. & Flenniken, M. L. Pathogen prevalence and abundance in honey bee colonies involved in almond pollination. *Apidologie* **47**, 251–266 (2016).

52. Ceballos, C., L. A. & Gurtler, R. E. Long-term reduction of trypanosoma cruzi infection in sylvatic mammals following deforestation and sustained vector surveillance in northwestern argentina. *Acta Trop* **98**, 286–96 (2006).

53. Chagas, S., Ecds & Guerra, M. Composition of sand fly fauna (diptera: Psychodidae) and detection of leishmania DNA (kinetoplastida: Trypanosomatidae) in different ecotopes from a rural settlement in the central amazon, brazil. *Parasit Vectors* **11**, 180 (2018).

54. Chargui, H., N. & Babba, H. Use of PCR, IFAT and in vitro culture in the detection of leishmania infantum infection in dogs and evaluation of the prevalence of canine leishmaniasis in a low endemic area in tunisia. *Parasite* **16**, 65–9 (2009).

55. Chemkhi, S., J. & Guerbouj, S. Natural infection of algerian hedgehog, atelerix algirus (lereboullet 1842) with leishmania parasites in tunisia. *Acta Trop* **150**, 42–51 (2015).

56. Cherenet, S., T. & Bossche, P. van den. Seasonal prevalence of bovine trypanosomosis in a tsetse-infested zone and a tsetse-free zone of the amhara region, north-west ethiopia. *Onderstepoort J Vet Res* **71**, 307–12 (2004).

57. Clausen, C., P. H. & Schein, E. A field study to estimate the prevalence of trypanosoma equiperdum in mongolian horses. *Vet Parasitol* **115**, 9–18 (2003).

58. Colla, O., Sheila R. & Thomson, J. D. Plight of the bumble bee: Pathogen spillover from commercial to wild populations. *Biological Conservation* **129**, 461–467 (2006).

59. Comeaux, C.-R., J. M. & Hamer, S. A. Survey of feral swine ( sus scrofa ) infection with the agent of chagas disease ( trypanosoma cruzi ) in texas, 2013-14. *J Wildl Dis* **52**, 627–30 (2016).

60. Cordes, H., N. & Solter, L. F. Interspecific geographic distribution and variation of the pathogens nosema bombi and crithidia species in united states bumble bee populations. *J Invertebr Pathol* **109**, 209–16 (2012).

61. Coura-Vital, M., W. & Carneiro, M. Prevalence and factors associated with leishmania infantum infection of dogs from an urban area of brazil as identified by molecular methods. *PLoS Negl Trop Dis* **5**, e1291 (2011).

62. Cringoli, R., G. & Capelli, G. Serological survey of neospora caninum and leishmania infantum co-infection in dogs. *Vet Parasitol* **106**, 307–13 (2002).

63. Crisante, R., G. & Anez, N. Infected dogs as a risk factor in the transmission of human trypanosoma cruzi infection in western venezuela. *Acta Trop* **98**, 247–54 (2006).

64. D’Alvise, S., P. & Hasselmann, M. Seasonal dynamics and co-occurrence patterns of honey bee pathogens revealed by high-throughput RT-qPCR analysis. *Ecol Evol* **9**, 10241–10252 (2019).

65. D’Andrea, F. E., L. A. & Tolezano, J. E. The shadows of a ghost: A survey of canine leishmaniasis in presidente prudente and its spatial dispersion in the western region of sao paulo state, an emerging focus of visceral leishmaniasis in brazil. *BMC Vet Res* **11**, 273 (2015).

66. Costa, C. da, A. P. & Marcili, A. Trypanosoma cruzi and leishmania infantum chagasi infection in wild mammals from maranhao state, brazil. *Vector Borne Zoonotic Dis* **15**, 656–66 (2015).

67. Davison, T., H. C. & Luckins, A. G. The occurrence of trypanosoma evansi in buffaloes in indonesia, estimated using various diagnostic tests. *Epidemiol Infect* **124**, 163–72 (2000).

68. Almeida Ado, S. de, B. & Madeira Mde, F. Canine visceral leishmaniasis: Seroprevalence and risk factors in cuiaba, mato grosso, brazil. *Rev Bras Parasitol Vet* **21**, 359–65 (2012).

69. Avelar, B. de, D. M. & Linardi, P. M. Endosymbionts of ctenocephalides felis felis (siphonaptera: Pulicidae) obtained from dogs captured in belo horizonte, minas gerais, brazil. *J Invertebr Pathol* **94**, 149–52 (2007).

70. Carvalho, L., A. G. & Fontes, C. J. F. High seroprevalence and peripheral spatial distribution of visceral leishmaniasis among domestic dogs in an emerging urban focus in central brazil: A cross-sectional study. *Pathog Glob Health* **112**, 29–36 (2018).

71. Matos, C., Amrn & Navarro, I. T. Antibodies anti-trypanosomatides in domestic cats in parana: Who is at highest risk of infection? *Rev Bras Parasitol Vet* **27**, 232–236 (2018).

72. Paula, C., M. B. & Costa-Cruz, J. M. Occurrence of positivity for trypanosoma cruzi in triatomine from municipalities in southeastern brazil, from 2002 to 2004. *Rev Soc Bras Med Trop* **43**, 9–14 (2010).

73. Thoisy, M. de, B. & Vie, J. C. A survey of hemoparasite infections in free-ranging mammals and reptiles in french guiana. *J Parasitol* **86**, 1035–40 (2000).

74. Delafosse, A. & Doutoum, A. A. Prevalence of trypanosoma evansi infection and associated risk factors in camels in eastern chad. *Vet Parasitol* **119**, 155–64 (2004).

75. Delafosse, T., A. & Michaux, Y. Epidemiology of trypanosoma vivax infection in cattle in the tse-tse free area of lake chad. *Prev Vet Med* **74**, 108–19 (2006).

76. Dennis, D., J. W. & MacLeod, E. T. Sodalis glossinidius prevalence and trypanosome presence in tsetse from luambe national park, zambia. *Parasit Vectors* **7**, 378 (2014).

77. Desquesnes, K., M. & Jittapalapong, S. Antibody-ELISA for trypanosoma evansi: Application in a serological survey of dairy cattle, thailand, and validation of a locally produced antigen. *Prev Vet Med* **90**, 233–41 (2009).

78. Dias, R.-S., E. S. & Fortes-Dias, C. L. Eco-epidemiology of visceral leishmaniasis in the urban area of paracatu, state of minas gerais, brazil. *Vet Parasitol* **176**, 101–11 (2011).

79. Dias, T.-S., R. C. F. & Navarro, I. T. Variables associated with the prevalence of anti-leishmania spp. Antibodies in dogs on the tri-border of foz do iguacu, parana, brazil. *Rev Bras Parasitol Vet* **27**, 338–347 (2018).

80. Dorn, D., P. L. & Phillippi-Falkenstein, K. M. Low prevalence of chagas parasite infection in a nonhuman primate colony in louisiana. *J Am Assoc Lab Anim Sci* **51**, 443–7 (2012).

81. Elmayan, T., A. & Dumonteil, E. High prevalence of trypanosoma cruzi infection in shelter dogs from southern louisiana, USA. *Parasit Vectors* **12**, 322 (2019).

82. Elshafie, R. A. H., E. I.Sani. Active infection and morphometric study of trypanosoma evansi among horses in peninsula malaysia. *Tropical Biomedicine* **30**, 444–450 (2013).

83. Felipe, A., I. M. & Caldas Ade, J. Leishmania infection in humans, dogs and sandflies in a visceral leishmaniasis endemic area in maranhao, brazil. *Mem Inst Oswaldo Cruz* **106**, 207–11 (2011).

84. Fernandez-Bellon, S.-G., H. & Ramis, A. Little evidence of seasonal variation of natural infection by leishmania infantum in dogs in spain. *Vet Parasitol* **155**, 32–6 (2008).

85. Ferroglio, B., E. & Biglino, A. Epidemiological evaluation of leishmania infantum zoonotic transmission risk in the recently established endemic area of northwestern italy. *Zoonoses Public Health* **65**, 675–682 (2018).

86. Figueiredo, M., F. B. & Schubach, T. M. Canine visceral leishmaniasis: Study of methods for the detection of IgG in serum and eluate samples. *Rev Inst Med Trop Sao Paulo* **52**, 193–6 (2010).

87. Fikru, G., R. & Buscher, P. Widespread occurrence of trypanosoma vivax in bovines of tsetse- as well as non-tsetse-infested regions of ethiopia: A reason for concern? *Vet Parasitol* **190**, 355–61 (2012).

88. Franca-Silva, da C., J. C. & Nascimento, E. Epidemiology of canine visceral leishmaniosis in the endemic area of montes claros municipality, minas gerais state, brazil. *Vet Parasitol* **111**, 161–73 (2003).

89. Freitas, N., R. A. & Barrett, T. V. Species diversity and flagellate infections in the sand fly fauna near porto grande, state of amapa, brazil (diptera: Psychodidae. Kinetoplastida: trypanosomatidae). *Mem Inst Oswaldo Cruz* **97**, 53–9 (2002).

90. Fujimori, A., M.de Almeida. Prevalence and associated factors of canine visceral leishmaniasis in an endemic area of mato grosso, brazil. *Acta Scientiae Veterinariae* **44**, (2016).

91. Gallot-Lavallee, S.-H., M. & Schmid-Hempel, P. Large scale patterns of abundance and distribution of parasites in mexican bumblebees. *J Invertebr Pathol* **133**, 73–82 (2016).

92. Galvez, M., R. & Molina, R. Emerging trends in the seroprevalence of canine leishmaniasis in the madrid region (central spain). *Vet Parasitol* **169**, 327–34 (2010).

93. Galvis-Ovallos, C., F. & Galati, E. A. B. Ecological parameters of the (s)-9-methylgermacrene-b population of the lutzomyia longipalpis complex in a visceral leishmaniasis area in sao paulo state, brazil. *Parasit Vectors* **10**, 269 (2017).

94. Garcia, R., H. A. & Teixeira, M. M. G. Remarkable richness of trypanosomes in tsetse flies (glossina morsitans morsitans and glossina pallidipes) from the gorongosa national park and niassa national reserve of mozambique revealed by fluorescent fragment length barcoding (FFLB). *Infect Genet Evol* **63**, 370–379 (2018).

95. Garcia, G., H. & Mendoza-Leon, A. Trypanosomiasis in venezuelan water buffaloes: Association of packed-cell volumes with seroprevalence and current trypanosome infection. *Ann Trop Med Parasitol* **100**, 297–305 (2006).

96. Garvin, S., M. C. & Moore, F. R. Blood parasites of nearctic-neotropical migrant passerine birds during spring trans-gulf migration: Impact on host body condition. *J Parasitol* **92**, 990–6 (2006).

97. Gebre-Michael, B., T. & Gramiccia, M. The isolation of leishmania tropica and l. Aethiopica from phlebotomus (paraphlebotomus) species (diptera: Psychodidae) in the awash valley, northeastern ethiopia. *Trans R Soc Trop Med Hyg* **98**, 64–70 (2004).

98. Ghawar, T., W. & Ben-Salah, A. Leishmania major infection among psammomys obesus and meriones shawi: Reservoirs of zoonotic cutaneous leishmaniasis in sidi bouzid(central tunisia). *Vector Borne Zoonotic Dis* **11**, 1561–8 (2011).

99. Gillespie, S. Factors affecting parasite prevalence among wild bumblebees. *Ecological Entomology* **35**, 737–747 (2010).

100. Gillingwater, M., K. & Majiwa, P. A. Prevalence of mixed trypanosoma congolense infections in livestock and tsetse in KwaZulu-natal, south africa. *J S Afr Vet Assoc* **81**, 219–23 (2010).

101. Giorgobiani, C., E. & Sacks, D. Epidemiologic aspects of an emerging focus of visceral leishmaniasis in tbilisi, georgia. *PLoS Negl Trop Dis* **5**, e1415 (2011).

102. Gizzarelli, F. M., M. & Maurelli, M. P. Simultaneous detection of parasitic vector borne diseases: A robust cross-sectional survey in hunting, stray and sheep dogs in a mediterranean area. *Front Vet Sci* **6**, 288 (2019).

103. Godoi, S., M. M. & Camargo, E. P. A PCR-based survey on phytomonas (euglenozoa: Trypanosomatidae) in phytophagous hemipterans of the amazon region. *J Eukaryot Microbiol* **49**, 275–9 (2002).

104. Goossens, M., B. & Vreysen, M. Trypanosomosis prevalence in cattle on mafia island (tanzania). *Vet Parasitol* **139**, 74–83 (2006).

105. Goulson, O., Dave & Park, K. J. The impacts of predators and parasites on wild bumblebee colonies. *Ecological Entomology* **43**, 168–181 (2018).

106. Goulson, W., Dave & Fowley, M. Influence of urbanisation on the prevalence of protozoan parasites of bumblebees. *Ecological Entomology* **37**, 83–89 (2012).

107. Castelli, L. *et al.* Detection of lotmaria passim in africanized and european honey bees from uruguay, argentina and chile. *Journal of Invertebrate Pathology* **160**, (2018).

108. Grijalva, V., M. J. & Baus, E. G. Comprehensive survey of domiciliary triatomine species capable of transmitting chagas disease in southern ecuador. *PLoS Negl Trop Dis* **9**, e0004142 (2015).

109. Grybchuk-Ieremenko, L., A. & Yurchenko, V. High prevalence of trypanosome co-infections in freshwater fishes. *Folia Parasitol (Praha)* **61**, 495–504 (2014).

110. Gutierrez, J., C. & Montoya, J. A. Camel trypanosomosis in the canary islands: Assessment of seroprevalence and infection rates using the card agglutination test (CATT/t. Evansi) and parasite detection tests. *Veterinary Parasitology* **90**, 155–159 (2000).

111. Hagos, A., A. & Claes, F. Serological and parasitological survey of dourine in the arsi-bale highlands of ethiopia. *Trop Anim Health Prod* **42**, 769–76 (2010).

112. Hagos, D., A. & Goddeeris, B. M. Seroepidemiological survey of trypanozoon infection in horses in the suspected dourine-infected bale highlands of the oromia region, ethiopia. *Rev Sci Tech* **29**, 649–54 (2010).

113. Hamel, S., D. & Rehbein, S. Parasites and vector-borne pathogens in client-owned dogs in albania. Blood pathogens and seroprevalences of parasitic and other infectious agents. *Parasitol Res* **115**, 489–99 (2016).

114. Hauptmanova, V. L., K.Benedikt. Blood parasites in passerine birds in slovakian east carpathians. *Acta Protozoologica* **45**, 105–109 (2006).

115. Herrera, D., H. M. & Jansen, A. M. Enzootiology of trypanosoma evansi in pantanal, brazil. *Vet Parasitol* **125**, 263–75 (2004).

116. Hodo, G., C. L. & Hamer, S. A. Trypanosome species, including trypanosoma cruzi, in sylvatic and peridomestic bats of texas, USA. *Acta Trop* **164**, 259–266 (2016).

117. Hosseininejad, M. H., M.Mohebali. Seroprevalence of canine visceral leishmaniasis in asymptomatic dogs in iran. *Iranian Journal of Veterinary Research* **13**, 54–57 (2012).

118. Isaac, C., C. & Turner, C. M. Molecular identification of different trypanosome species and subspecies in tsetse flies of northern nigeria. *Parasit Vectors* **9**, 301 (2016).

119. Jabal-Uriel, M.-H., Clara & De la Rua, P. Short communication: First data on the prevalence and distribution of pathogens in bumblebees (bombus terrestris and bombus pascuorum) from spain. *Spanish Journal of Agricultural Research* **15**, (2017).

120. Jaimes-Duenez, Z.-Z., J. & Mejia-Jaramillo, A. M. Evaluation of an alternative indirect-ELISA test using in vitro-propagated trypanosoma brucei brucei whole cell lysate as antigen for the detection of anti-trypanosoma evansi IgG in colombian livestock. *Prev Vet Med* **169**, 104712 (2019).

121. Jaimes-Duenez, T.-C., J. & Mejia-Jaramillo, A. M. Parasitological and molecular surveys reveal high rates of infection with vector-borne pathogens and clinical anemia signs associated with infection in cattle from two important livestock areas in colombia. *Ticks Tick Borne Dis* **8**, 290–299 (2017).

122. Jiang, W., W. & Wang, Q. Seroepidemiological study of canine leishmania infantum and toxoplasma gondii infections in shanghai, china, and analysis of risk factors. *Ann Agric Environ Med* **23**, 420–4 (2016).

123. Karakus, T., M. & Ozbel, Y. Evaluation of conjunctival swab sampling in the diagnosis of canine leishmaniasis: A two-year follow-up study in cukurova plain, turkey. *Vet Parasitol* **214**, 295–302 (2015).

124. Karlsbakk, E. Prevalence of trypanosome infections in marine fishes from western norway. *Sarsia* **89**, 459–466 (2010).

125. Karshima, A., S. N. & Mohammed, G. Eco-epidemiology of porcine trypanosomosis in karim lamido, nigeria: Prevalence, seasonal distribution, tsetse density and infection rates. *Parasit Vectors* **9**, 448 (2016).

126. Kassahun, S., A. & Votypka, J. Detection of leishmania donovani and l. Tropica in ethiopian wild rodents. *Acta Trop* **145**, 39–44 (2015).

127. Katakweba, M., Abdul A. S. & Belmain, S. R. Prevalence of haemoparasites, leptospires and coccobacilli with potential for human infection in the blood of rodents and shrews from selected localities in tanzania, namibia and swaziland†. *African Zoology* **47**, 119–127 (2012).

128. Kato, G., Hirotomo & Cáceres, A. G. Molecular mass screening to incriminate sand fly vectors of andean-type cutaneous leishmaniasis in ecuador and peru. *The American Journal of Tropical Medicine and Hygiene* **79**, 719–721 (2008).

129. Kato, C., H. & Hashiguchi, Y. First evidence of a hybrid of leishmania (viannia) braziliensis/l. (V.) Peruviana DNA detected from the phlebotomine sand fly lutzomyia tejadai in peru. *PLoS Negl Trop Dis* **10**, e0004336 (2016).

130. Khan, A. S. H., A. U.Qureshi. Molecular identification of trypanosomes and their effects on hematological and biochemical parameters in donkeys in punjab, pakistan. *International Journal of Agriculture and Biology* **20**, 1607–1612 (2018).

131. Kidanemariam, H., A. & Sahle, M. Parasitological prevalence of bovine trypanosomosis in kindo koisha district, wollaita zone, south ethiopia. *Onderstepoort J Vet Res* **69**, 107–13 (2002).

132. Kissinger, C., C. N. & Solter, L. F. Survey of bumble bee (bombus) pathogens and parasites in illinois and selected areas of northern california and southern oregon. *J Invertebr Pathol* **107**, 220–4 (2011).

133. Kocher, D., A. & Jittapalapong, S. Evaluation of an indirect-ELISA test for trypanosoma evansi infection (surra) in buffaloes and its application to a serological survey in thailand. *Biomed Res Int* **2015**, 361037 (2015).

134. Korner, P. & Schmid-Hempel, P. Correlates of parasite load in bumblebees in an alpine habitat. *Entomological Science* **8**, 151–160 (2005).

135. Kouam, D., M. K. & Theodoropoulos, G. A seroepidemiological study of exposure to toxoplasma, leishmania, echinococcus and trichinella in equids in greece and analysis of risk factors. *Vet Parasitol* **170**, 170–5 (2010).

136. Kozminsky, K., E. & Maslov, D. A. Host-specificity of monoxenous trypanosomatids: Statistical analysis of the distribution and transmission patterns of the parasites from neotropical heteroptera. *Protist* **166**, 551–68 (2015).

137. Lana, M., R. S. & Dias, E. S. Ecoepidemiological aspects of visceral leishmaniasis in an endemic area in the steel valley in brazil: An ecological approach with spatial analysis. *PLoS One* **13**, e0206452 (2018).

138. Laohasinnarong, G., D. & Namangala, B. Studies of trypanosomiasis in the luangwa valley, north-eastern zambia. *Parasit Vectors* **8**, 497 (2015).

139. Lardeux, A., F. & Depickere, S. Bias due to methods of parasite detection when estimating prevalence of infection of triatoma infestans by trypanosoma cruzi. *J Vector Ecol* **41**, 285–291 (2016).

140. Lauricella, S., M. A. & Gurtler, R. E. Distribution and pathogenicity of trypanosoma cruzi isolated from peridomestic populations of triatoma infestans and triatoma guasayana from rural western argentina. *Mem Inst Oswaldo Cruz* **100**, 123–9 (2005).

141. Leal, C., G. G. A. & Coura-Vital, W. Risk profile for leishmania infection in dogs coming from an area of visceral leishmaniasis reemergence. *Prev Vet Med* **150**, 1–7 (2018).

142. Lledo, G.-P., L. & Serrano, J. L. Wild red foxes (vulpes vulpes) as sentinels of parasitic diseases in the province of soria, northern spain. *Vector Borne Zoonotic Dis* **15**, 743–9 (2015).

143. Lopez-Cespedes, L., A. & Marin, C. Leishmania spp. Epidemiology of canine leishmaniasis in the yucatan peninsula. *ScientificWorldJournal* **2012**, 945871 (2012).

144. Lopez-Cespedes, L., A. & Marin, C. Seroprevalence of antibodies against the excreted antigen superoxide dismutase by trypanosoma cruzi in dogs from the yucatan peninsula (mexico). *Zoonoses Public Health* **60**, 277–83 (2013).

145. Machila, S., N. & Eisler, M. C. Antibody-ELISA seroprevalence of bovine trypanosomosis in the eastern province of zambia. *Prev Vet Med* **49**, 249–57 (2001).

146. Magallon-Gastelum, L.-K., E. & Breniere, S. F. Epidemiological risk for trypanosoma cruzi transmission by species of phyllosoma complex in the occidental part of mexico. *Acta Trop* **97**, 331–8 (2006).

147. Maia, R., C. & Campino, L. Bacterial and protozoal agents of feline vector-borne diseases in domestic and stray cats from southern portugal. *Parasit Vectors* **7**, 115 (2014).

148. Majekodunmi, F., A. O. & Welburn, S. C. A longitudinal survey of african animal trypanosomiasis in domestic cattle on the jos plateau, nigeria: Prevalence, distribution and risk factors. *Parasit Vectors* **6**, 239 (2013).

149. Malele, C., Imna & Gibson, W. The use of specific and generic primers to identify trypanosome infections of wild tsetse flies in tanzania by PCR. *Infection, Genetics and Evolution* **3**, 271–279 (2003).

150. Malfi, R. L. & Roulston, T. H. Patterns of parasite infection in bumble bees (bombusspp.) Of northern virginia. *Ecological Entomology* **39**, 17–29 (2014).

151. Maloney, N., J. & Moncayo, A. C. Seroprevalence of trypanosoma cruzi in raccoons from tennessee. *J Parasitol* **96**, 353–8 (2010).

152. Mamoudou, N., A. & Achukwi, M. D. Animal trypanosomosis in clinically healthy cattle of north cameroon: Epidemiological implications. *Parasit Vectors* **9**, 206 (2016).

153. Marti, E., G. A. & Garcia, J. J. Prevalence and distribution of parasites and pathogens of triatominae from argentina, with emphasis on triatoma infestans and triatoma virus TrV. *J Invertebr Pathol* **102**, 233–7 (2009).

154. Martin-Sanchez, G., J. & Morillas-Marquez, F. Pool screen PCR for estimating the prevalence of leishmania infantum infection in sandflies (diptera: Nematocera, phlebotomidae). *Trans R Soc Trop Med Hyg* **100**, 527–32 (2006).

155. Martinez-Hernandez, R.-F., F. & Villalobos, G. Follow up of natural infection with trypanosoma cruzi in two mammals species, nasua narica and procyon lotor (carnivora: Procyonidae): Evidence of infection control? *Parasit Vectors* **7**, 405 (2014).

156. Mascolli, S., Roberta & Vasconcellos, S. A. Prevalence and risk factors for leishmaniasis and chagas disease in the canine population of the tourist city of ibiúna, são paulo, brazil. *Semina: Ciências Agrárias* **37**, 1971–1980 (2016).

157. Maslov, W., D. A. & Sturm, N. R. Discovery and barcoding by analysis of spliced leader RNA gene sequences of new isolates of trypanosomatidae from heteroptera in costa rica and ecuador. *J Eukaryot Microbiol* **54**, 57–65 (2007).

158. Mejia-Jaramillo, A.-U., A. M. & Triana-Chavez, O. Genotyping of trypanosoma cruzi in a hyper-endemic area of colombia reveals an overlap among domestic and sylvatic cycles of chagas disease. *Parasit Vectors* **7**, 108 (2014).

159. Mekata, K., H. & Ohashi, K. Prevalence and source of trypanosome infections in field-captured vector flies (glossina pallidipes) in southeastern zambia. *J Vet Med Sci* **70**, 923–8 (2008).

160. Mekata, K., H. & Ohashi, K. Molecular detection of trypanosomes in cattle in south america and genetic diversity of trypanosoma evansi based on expression-site-associated gene 6. *Infect Genet Evol* **9**, 1301–5 (2009).

161. Mekibib, M. T., B.Manegerew. Prevalence of haemoparasites and associated risk factors in working donkeys in adigudem and kwiha districts of tigray region, northern ethiopia. *Journal of Animal and Veterinary Advances* **9**, 2249–2255 (2010).

162. Mekonnen, V. K., B.Regassa. Epidemiology of trypanosomosis in goats in abelti, bede and ghibe valley, south west ethiopia. *International Journal of Tropical Medicine* **9**, 10–14 (2014).

163. Araujo Melo, B. de, S. & Abreu-Silva, A. L. Bovine trypanosomiasis an emerging disease in maranhao state-brazil. *Vector Borne Zoonotic Dis* **11**, 853–6 (2011).

164. Menezes, F. E., J. A. & Margonari, C. An integrated approach using spatial analysis to study the risk factors for leishmaniasis in area of recent transmission. *Biomed Res Int* **2015**, 621854 (2015).

165. Meyers, M., A. C. & Hamer, S. A. Widespread trypanosoma cruzi infection in government working dogs along the texas-mexico border: Discordant serology, parasite genotyping and associated vectors. *PLoS Negl Trop Dis* **11**, e0005819 (2017).

166. Mihret, A. & Mamo, G. Bovine trypanosomosis in three districts of east gojjam zone bordering the blue nile river in ethiopia. *J Infect Dev Ctries* **1**, 321–5 (2007).

167. Miro, C., G. & Galvez, R. Current situation of leishmania infantum infection in shelter dogs in northern spain. *Parasit Vectors* **5**, 60 (2012).

168. Miro, M., G. & Molina, R. A leishmaniosis surveillance system among stray dogs in the region of madrid: Ten years of serodiagnosis (1996-2006). *Parasitol Res* **101**, 253–7 (2007).

169. Mohebali, H., M. & Fakhar, M. Epidemiological aspects of canine visceral leishmaniosis in the islamic republic of iran. *Vet Parasitol* **129**, 243–51 (2005).

170. Molina, R., J. M. & Gutiérrez, C. Seroprevalence of trypanosoma evansi in dromedaries (camelus dromedarius) from the canary islands (spain) using an antibody ab-ELISA. *Preventive Veterinary Medicine* **47**, 53–59 (2000).

171. Monroy, R., C. & Tabaru, Y. Epidemiology of chagas disease in guatemala: Infection rate of triatoma dimidiata, triatoma nitida and rhodnius prolixus (hemiptera, reduviidae) with trypanosoma cruzi and trypanosoma rangeli (kinetoplastida, trypanosomatidae). *Mem Inst Oswaldo Cruz* **98**, 305–10 (2003).

172. Morimoto, K., T. & Kadowaki, T. Molecular detection of protozoan parasites infecting apis mellifera colonies in japan. *Environ Microbiol Rep* **5**, 74–7 (2013).

173. Muhanguzi, M., D. & Tweyongyere, R. African animal trypanosomiasis as a constraint to livestock health and production in karamoja region: A detailed qualitative and quantitative assessment. *BMC Vet Res* **13**, 355 (2017).

174. Muhanguzi, P., D. & Welburn, S. C. The burden and spatial distribution of bovine african trypanosomes in small holder crop-livestock production systems in tororo district, south-eastern uganda. *Parasit Vectors* **7**, 603 (2014).

175. N’Djetchi, I., M. K. & Jamonneau, V. The study of trypanosome species circulating in domestic animals in two human african trypanosomiasis foci of cote d’ivoire identifies pigs and cattle as potential reservoirs of trypanosoma brucei gambiense. *PLoS Negl Trop Dis* **11**, e0005993 (2017).

176. Nakayima, N., J. & Sugimoto, C. Molecular epidemiological studies on animal trypanosomiases in ghana. *Parasit Vectors* **5**, 217 (2012).

177. Sachman-Ruiz, N.-P., Bernardo & Reynaud, E. Commercial bombus impatiens as reservoirs of emerging infectious diseases in central méxico. *Biological Invasions* **17**, 2043–2053 (2015).

178. Ngaira, B., J. M. & Karanja, S. M. Animal-level risk factors for trypanosoma evansi infection in camels in eastern and central parts of kenya. *Onderstepoort J Vet Res* **69**, 263–71 (2002).

179. Ngonyoka, G., A. & Cattadori, I. M. Patterns of tsetse abundance and trypanosome infection rates among habitats of surveyed villages in maasai steppe of northern tanzania. *Infect Dis Poverty* **6**, 126 (2017).

180. Mbang Nguema, B. A., O. & Mawili Mboumba, D. P. Variations of glossina sp. And trypanosome species frequency within different habitats in a sleeping sickness focus, gabon. *J Infect Dev Ctries* **13**, 67–72 (2019).

181. Njiokou, N., F. & Herder, S. Domestic animals as potential reservoir hosts of trypanosoma brucei gambiense in sleeping sickness foci in cameroon. *Parasite* **17**, 61–6 (2010).

182. Njiru, C., Z. K. & Reid, S. A. Detection of trypanosoma evansi in camels using PCR and CATT/t. Evansi tests in kenya. *Vet Parasitol* **124**, 187–99 (2004).

183. Northover, G., A. S. & Thompson, R. C. A. Increased trypanosoma spp. Richness and prevalence of haemoparasite co-infection following translocation. *Parasit Vectors* **12**, 126 (2019).

184. Oakgrove, H., K. S. & Sehgal, R. N. Distribution, diversity and drivers of blood-borne parasite co-infections in alaskan bird populations. *Int J Parasitol* **44**, 717–27 (2014).

185. Odeniran, M., P. O. & Welburn, S. C. Molecular identification of bovine trypanosomes in relation to cattle sources in southwest nigeria. *Parasitol Int* **68**, 1–8 (2019).

186. Oliveira, P., G. C. & Langoni, H. Antibodies to leishmania spp. In domestic felines. *Rev Bras Parasitol Vet* **24**, 464–70 (2015).

187. Onyekwelu, F. E. E., K. C.Ejezie. Molecular identification of trypanosomes in tsetse flies trapped from onicha ugbo in delta state of nigeria. *Biomedical Research-India* **28**, 5463–5467 (2017).

188. Opara, B. O., M. N.Fagbemi. Animal biodiversity and emerging diseases: Prediction and prevention. **1149**, 394–397 (2008).

189. Oshaghi, R., M. A. & Rassi, Y. Vector incrimination of sand flies in the most important visceral leishmaniasis focus in iran. *Am J Trop Med Hyg* **81**, 572–7 (2009).

190. Otranto, N., D. & Brianti, E. Feline and canine leishmaniosis and other vector-borne diseases in the aeolian islands: Pathogen and vector circulation in a confined environment. *Vet Parasitol* **236**, 144–151 (2017).

191. Otranto, T., D. & Breitschwerdt, E. B. Diagnosis of canine vector-borne diseases in young dogs: A longitudinal study. *J Clin Microbiol* **48**, 3316–24 (2010).

192. Ouedraogo, D.-U., G. M. S. & Abd-Alla, A. M. M. Prevalence of trypanosomes, salivary gland hypertrophy virus and wolbachia in wild populations of tsetse flies from west africa. *BMC Microbiol* **18**, 153 (2018).

193. Ozbel, O., Y. & Ozcel, M. A. A survey on canine leishmaniasis in western turkey by parasite, DNA and antibody detection assays. *Acta Trop* **74**, 1–6 (2000).

194. Paiz, F., L. M. & Langoni, H. Serological evidence of infection by leishmania (leishmania) infantum (synonym: Leishmania (leishmania) chagasi) in free-ranging wild mammals in a nonendemic region of the state of sao paulo, brazil. *Vector Borne Zoonotic Dis* **15**, 667–73 (2015).

195. Parashar, S., R. & Bal, M. S. Unraveling cryptic epizootiology of equid trypanosomosis in punjab state of india by parasitological and sero-molecular techniques. *Acta Trop* **185**, 18–26 (2018).

196. Paz, R., G. F. & Dias, E. S. Association between the prevalence of infestation by rhipicephalus sanguineus and ctenocephalides felis felis and the presence of anti-leishmania antibodies: A case-control study in dogs from a brazilian endemic area. *Prev Vet Med* **97**, 131–3 (2010).

197. Plischuk, A., S. & Lange, C. E. Long-term prevalence of the protists crithidia bombi and apicystis bombi and detection of the microsporidium nosema bombi in invasive bumble bees. *Environ Microbiol Rep* **9**, 169–173 (2017).

198. Popp, E., M. & Lattorff, H. M. Seasonal variability of prevalence and occurrence of multiple infections shape the population structure of crithidia bombi, an intestinal parasite of bumblebees (bombus spp.). *Microbiologyopen* **1**, 362–72 (2012).

199. Rangel, L., D. A. & Roque, A. L. R. Isolation and characterization of trypanosomatids, including crithidia mellificae, in bats from the atlantic forest of rio de janeiro, brazil. *PLoS Negl Trop Dis* **13**, e0007527 (2019).

200. Ravel, M., S. & Davoust, B. A study on african animal trypanosomosis in four areas of senegal. *Folia Parasitol (Praha)* **62**, (2015).

201. Ravoet, M., J. & Graaf, D. C. de. Comprehensive bee pathogen screening in belgium reveals crithidia mellificae as a new contributory factor to winter mortality. *PLoS One* **8**, e72443 (2013).

202. Reid, S. A. & Copeman, D. B. Surveys in papua new guinea to detect the presence of trypanosoma evansi infection. *Aust Vet J* **78**, 843–5 (2000).

203. Reithinger, E., R. & Davies, C. R. Evaluation of PCR as a diagnostic mass-screening tool to detect leishmania (viannia) spp. In domestic dogs (canis familiaris). *J Clin Microbiol* **41**, 1486–93 (2003).

204. Reithinger, B. E. B., R.Lambson. Use of PCR to detect leishmania (viannia) spp. In dog blood and bone marrow. *Journal of Clinical Microbiology* **38**, 748–751 (2000).

205. Resadore, J., F. & Medeiros, J. F. Composition and vertical stratification of phlebotomine sand fly fauna and the molecular detection of leishmania in forested areas in rondonia state municipalities, western amazon, brazil. *Vector Borne Zoonotic Dis* **19**, 347–357 (2019).

206. Ribeiro, M., A. R. & Rosa, J. A. da. Trypanosoma cruzi strains from triatomine collected in bahia and rio grande do sul, brazil. *Rev Saude Publica* **48**, 295–302 (2014).

207. Rocha, M., Avvo & Costa, A. P. D. Diagnosis and epidemiology of leishmania infantum in domestic cats in an endemic area of the amazon region, brazil. *Vet Parasitol* **273**, 80–85 (2019).

208. Rodrigues, S., A. C. & Bevilaqua, C. M. Epidemiological survey of lutzomyia longipalpis infected by leishmania infantum in an endemic area of brazil. *Rev Bras Parasitol Vet* **23**, 55–62 (2014).

209. Rodriguez, T.-J., N. F. & Gutierrez, C. Cross-sectional study on prevalence of trypanosoma evansi infection in domestic ruminants in an endemic area of the canary islands (spain). *Prev Vet Med* **105**, 144–8 (2012).

210. Rohousova, T.-F., I. & Baneth, G. Exposure to leishmania spp. And sand flies in domestic animals in northwestern ethiopia. *Parasit Vectors* **8**, 360 (2015).

211. Rondon, B., F. C. & Diniz, A. T. Cross-sectional serological study of canine leishmania infection in fortaleza, ceara state, brazil. *Vet Parasitol* **155**, 24–31 (2008).

212. Rowland, M., M. E. & Moncayo, A. C. Factors associated with trypanosoma cruzi exposure among domestic canines in tennessee. *J Parasitol* **96**, 547–51 (2010).

213. Saghafipour, V., A. & Hanafi-Bojd, A. A. Epidemiological study on cutaneous leishmaniasis in an endemic area, of qom province, central iran. *J Arthropod Borne Dis* **11**, 403–413 (2017).

214. Salim, B., B. & Sugimoto, C. Molecular epidemiology of camel trypanosomiasis based on ITS1 rDNA and RoTat 1.2 VSG gene in the sudan. *Parasit Vectors* **4**, 31 (2011).

215. Salim, B., B. & Sugimoto, C. Molecular detection of equine trypanosomes in the sudan. *Vet Parasitol* **200**, 246–50 (2014).

216. Santos, G., Helcileia Dias & Minharro, S. High frequency of visceral leishmaniasis in dogs under veterinary clinical care in an intense transmission area in the state of tocantins, brazil. *Ciência Rural* **47**, (2017).

217. Saraiva, L., L. & Andrade, J. D. F. Seasonality of sand flies (diptera: Psychodidae) and leishmania DNA detection in vector species in an area with endemic visceral leishmaniasis. *Mem Inst Oswaldo Cruz* **112**, 309–318 (2017).

218. Sarquis, B.-P., O. & Lima, M. M. Epidemiology of chagas disease in jaguaruana, ceara, brazil. I. Presence of triatomines and index of trypanosoma cruzi infection in four localities of a rural area. *Mem Inst Oswaldo Cruz* **99**, 263–70 (2004).

219. Sauda, M., F. & Perrucci, S. Leishmania infantum, dirofilaria spp. And other endoparasite infections in kennel dogs in central italy. *Parasite* **25**, 2 (2018).

220. Savage, R., A. F. & Greiner, E. C. Blood parasites in birds from madagascar. *J Wildl Dis* **45**, 907–20 (2009).

221. Schoener, U., E. & Fuehrer, H. P. Trypanosomatid parasites in austrian mosquitoes. *PLoS One* **13**, e0196052 (2018).

222. Schultz, U., Albert & Underhill, G. Infection prevalence and absence of positive correlation between avian haemosporidian parasites, mass and body condition in the cape weaver ploceus capensis. *Ostrich* **81**, 69–76 (2010).

223. Sebaio, B., Fabiane & Marini, M. Ân. Blood parasites in brazilian atlantic forest birds: Effects of fragment size and habitat dependency. *Bird Conservation International* **20**, 432–439 (2010).

224. Sebaio, B., F. & Marini, M. A. Blood parasites in passerine birds from the brazilian atlantic forest. *Rev Bras Parasitol Vet* **21**, 7–15 (2012).

225. Seck, B., M. T. & Vreysen, M. J. The prevalence of african animal trypanosomoses and tsetse presence in western senegal. *Parasite* **17**, 257–65 (2010).

226. Sehgal, J., R. N. & Smith, T. B. Host specificity and incidence of trypanosoma in some african rainforest birds: A molecular approach. *Mol Ecol* **10**, 2319–27 (2001).

227. Shahzad, M., W. & Iqbal, M. Prevalence and molecular diagnosis of trypanosoma evansi in nili-ravi buffalo (bubalus bubalis) in different districts of punjab (pakistan). *Trop Anim Health Prod* **42**, 1597–9 (2010).

228. Sharma, D. S., A. & Juyal, P. D. Molecular prevalence of babesia bigemina and trypanosoma evansi in dairy animals from punjab, india, by duplex PCR: A step forward to the detection and management of concurrent latent infections. *Biomed Res Int* **2013**, 893862 (2013).

229. Sharma, J., P. & Pawar, H. Comparative evaluation of real time PCR assay with conventional parasitological techniques for diagnosis of trypanosoma evansi in cattle and buffaloes. *Vet Parasitol* **190**, 375–82 (2012).

230. Sheferaw, B., D. & Woldesenbet, Z. Bovine trypanosomosis and glossina distribution in selected areas of southern part of rift valley, ethiopia. *Acta Trop* **154**, 145–8 (2016).

231. Shereni, A., W. & Cecchi, G. Spatial distribution and trypanosome infection of tsetse flies in the sleeping sickness focus of zimbabwe in hurungwe district. *Parasit Vectors* **9**, 605 (2016).

232. Silva, P., R. B. S. & Melo, M. A. Seroprevalence and risk factors associated with canine visceral leishmaniasis in the state of paraiba, brazil. *Rev Soc Bras Med Trop* **51**, 683–688 (2018).

233. Simo, S., G. & Asonganyi, T. Identification and genetic characterization of trypanosoma congolense in domestic animals of fontem in the south-west region of cameroon. *Infect Genet Evol* **18**, 66–73 (2013).

234. Simukoko, M., H. & Van den Bossche, P. The comparative role of cattle, goats and pigs in the epidemiology of livestock trypanosomiasis on the plateau of eastern zambia. *Vet Parasitol* **147**, 231–8 (2007).

235. Simwango, N., M. & Gwakisa, P. S. Molecular prevalence of trypanosome infections in cattle and tsetse flies in the maasai steppe, northern tanzania. *Parasit Vectors* **10**, 507 (2017).

236. Singh, A. K. S., A. P.Tripathi. Seroprevalence of trypanosoma evansi in buffaloes in south western semiarid plane zone of uttarpradesh. *Buffalo Bulletin* **36**, 483–488 (2017).

237. Sinshaw, A., A. & Yoni, W. Biting flies and trypanosoma vivax infection in three highland districts bordering lake tana, ethiopia. *Vet Parasitol* **142**, 35–46 (2006).

238. Sousa, L., S. & Cordeiro da Silva, A. Seroepidemiological survey of leishmania infantum infection in dogs from northeastern portugal. *Acta Trop* **120**, 82–7 (2011).

239. Specht, E. J. Prevalence of bovine trypanosomosis in central mozambique from 2002 to 2005. *Onderstepoort J Vet Res* **75**, 73–81 (2008).

240. Suh, N., P. F. & Garabed, R. Bovine trypanosomiasis in tsetse-free pastoral zone of the far-north region, cameroon. *J Vector Borne Dis* **54**, 263–269 (2017).

241. Svobodova, V., M. & Votypka, J. Trypanosomatids in ornithophilic bloodsucking diptera. *Med Vet Entomol* **29**, 444–7 (2015).

242. Takeet, F., M. I. & Imumorin, I. G. Molecular survey of pathogenic trypanosomes in naturally infected nigerian cattle. *Res Vet Sci* **94**, 555–61 (2013).

243. Tasew, R., S.Duguma. Cattle anaemia and trypanosomiasis in western oromia state, ethiopia. *Revue De Medecine Veterinaire* **163**, 581–588 (2012).

244. Tatard, G., C. & Dobigny, G. Rodent-borne trypanosoma from cities and villages of niger and nigeria: A special role for the invasive genus rattus? *Acta Trop* **171**, 151–158 (2017).

245. Tehseen, J., S. & Buscher, P. Parasitological, serological and molecular survey of trypanosoma evansi infection in dromedary camels from cholistan desert, pakistan. *Parasit Vectors* **8**, 415 (2015).

246. Teles, S., C. B. & Camargo, L. M. Phlebotomine sandfly (diptera: Psychodidae) diversity and their leishmania DNA in a hot spot of american cutaneous leishmaniasis human cases along the brazilian border with peru and bolivia. *Mem Inst Oswaldo Cruz* **0**, 0 (2016).

247. Terefe, H., E. & Mwai, O. Phenotypic characteristics and trypanosome prevalence of mursi cattle breed in the bodi and mursi districts of south omo zone, southwest ethiopia. *Trop Anim Health Prod* **47**, 485–93 (2015).

248. Thies, R., S. F. & Dias, E. S. Phlebotomine sandfly fauna and natural leishmania infection rates in a rural area of cerrado (tropical savannah) in nova mutum, state of mato grosso in brazil. *Rev Soc Bras Med Trop* **46**, 293–8 (2013).

249. Tiwary, K., P. & Sundar, S. Prevalence of sand flies and leishmania donovani infection in a natural population of female phlebotomus argentipes in bihar state, india. *Vector Borne Zoonotic Dis* **12**, 467–72 (2012).

250. Tognazzo, S.-H., M. & Schmid-Hempel, P. Probing mixed-genotype infections II: High multiplicity in natural infections of the trypanosomatid, crithidia bombi, in its host, bombus spp. *PLoS One* **7**, e49137 (2012).

251. Tripodi, C.-S., Amber D. & Szalanski, A. L. Nosema bombi(microsporidia: Nosematidae) and trypanosomatid prevalence in spring bumble bee queens (hymenoptera: Apidae:bombus) in kansas. *Journal of the Kansas Entomological Society* **87**, 225–233 (2014).

252. Tripodi, S., A. D. & Strange, J. P. Novel multiplex PCR reveals multiple trypanosomatid species infecting north american bumble bees (hymenoptera: Apidae: bombus). *J Invertebr Pathol* **153**, 147–155 (2018).

253. Vanderplanck, R., Maryse & Michez, D. Bumble bee parasite prevalence but not genetic diversity impacted by the invasive plant impatiens glandulifera. *Ecosphere* **10**, (2019).

254. Vavilova, K., V. Y. & Blinov, A. G. Parasites of the genus nosema, crithidia and lotmaria in the honeybee and bumblebee populations: A case study in india. *Vavilov Journal of Genetics and Breeding* **21**, 943–951 (2017).

255. Villagran, M., M. E. & Diego, J. A. de. Natural infection and distribution of triatomines (hemiptera: Reduviidae) in the state of queretaro, mexico. *Trans R Soc Trop Med Hyg* **102**, 833–8 (2008).

256. Votypka, K., J. & Lukes, J. High prevalence and endemism of trypanosomatids on a small caribbean island. *J Eukaryot Microbiol* **66**, 600–607 (2019).

257. Votypka, M., J. & Lukes, J. Probing into the diversity of trypanosomatid flagellates parasitizing insect hosts in south-west china reveals both endemism and global dispersal. *Mol Phylogenet Evol* **54**, 243–53 (2010).

258. Votypka, S., J. & Yurchenko, V. Diversity of trypanosomatids (kinetoplastea: Trypanosomatidae) parasitizing fleas (insecta: Siphonaptera) and description of a new genus blechomonas gen. n. *Protist* **164**, 763–81 (2013).

259. Waiswa, O.-M., C. & Katunguka-Rwakishaya, E. Domestic animals as reservoirs for sleeping sickness in three endemic foci in south-eastern uganda. *Ann Trop Med Parasitol* **97**, 149–55 (2003).

260. Weber, N., J. S. & Kelm, S. Genetic diversity of trypanosome species in tsetse flies (glossina spp.) In nigeria. *Parasit Vectors* **12**, 481 (2019).

261. Wei, S., F. & Liu, Q. Molecular detection and genetic diversity of leishmania donovani in naturally infected phlebotomus chinensi from southwestern china. *Vector Borne Zoonotic Dis* **11**, 849–52 (2011).

262. Whitehorn, T., P. R. & Goulson, D. Genetic diversity, parasite prevalence and immunity in wild bumblebees. *Proc Biol Sci* **278**, 1195–202 (2011).

263. Whitehorn, T., Penelope R. & Goulson, D. Investigating the impact of deploying commercial bombus terrestris for crop pollination on pathogen dynamics in wild bumble bees. *Journal of Apicultural Research* **52**, 149–157 (2015).

264. Wilfert, L., L. & Jiggins, F. M. Trypanosomatids are common and diverse parasites of drosophila. *Parasitology* **138**, 858–65 (2011).

265. Xu, P.-Y., G. & Rich, S. Triplex real-time PCR for detection of crithidia mellificae and lotmaria passim in honey bees. *Parasitol Res* **117**, 623–628 (2018).

266. Yadav, K., S. C. & Kumar, R. Seroprevalence of trypanosoma evansi infection in equines of north and north western states of india. *J Equine Vet Sci* **79**, 63–67 (2019).

267. Zorrilla, D. L. S., V. & Valdivia, H. O. Distribution and identification of sand flies naturally infected with leishmania from the southeastern peruvian amazon. *PLoS Negl Trop Dis* **11**, e0006029 (2017).

268. Simo, G. *et al.* Population genetics of forest type of trypanosoma congolense circulating in glossina palpalis palpalis of fontem in the south-west region of cameroon. *Parasites & vectors* **7**, 385 (2014).

269. Simo, G. *et al.* Population genetics of trypanosoma brucei circulating in glossina palpalis palpalis and domestic animals of the fontem sleeping sickness focus of cameroon. *Parasites & vectors* **7**, 156 (2014).

270. Acosta, N. *et al.* Hosts and vectors of trypanosoma cruzi discrete typing units in the chagas disease endemic region of the paraguayan chaco. *Parasitology* **144**, 1–15 (2017).

271. Tome, R., Gaio, F., Generoso, D., Menozzi, B. & Langoni, H. Active surveillance of canine visceral leishmaniasis and american trypanossomiasis in rural dogs from non endemic area. *Revista brasileira de parasitologia veterinária = Brazilian journal of veterinary parasitology : Órgão Oficial do Colégio Brasileiro de Parasitologia Veterinária* **20**, 64–6 (2011).

272. Vargas, M. *et al.* Viral and intestinal diseases detected in apis mellifera in central and southern chile. *Chilean journal of agricultural research* **77**, 243–249 (2017).

273. Williams, M.-K., Tripodi, A. & Szalanski, A. Molecular survey for the honey bee (apis mellifera l.) Trypanosome parasites crithidia mellificae and lotmaria passim. *Journal of Apicultural Research* **58**, (2019).

274. Gomez, E. A., Kato, H. & Hashiguchi, Y. Man-biting sand fly species and natural infection with the leishmania promastigote in leishmaniasis-endemic areas of ecuador. *Acta Tropica* **140**, 41–49 (2014).

275. Abbaszadeh Afshar, S., M. J. & Naderi, A. Canine visceral leishmaniasis; a seroepidemiological survey in jiroft district, southern kerman province, southeastern iran in 2015. *Iran J Parasitol* **13**, 67–71 (2018).

276. Abdeen, S., Z. A. & Baneth, G. Epidemiology of visceral leishmaniasis in the jenin district, west bank: 1989-1998. *Am J Trop Med Hyg* **66**, 329–33 (2002).

277. Abebe, R. & Wolde, A. A cross-sectional study of trypanosomosis and its vectors in donkeys and mules in northwest ethiopia. *Parasitol Res* **106**, 911–6 (2010).

278. Abrantes, M. M., T. R. & Figueiredo, F. B. Identification of canine visceral leishmaniasis in a previously unaffected area by conventional diagnostic techniques and cell-block fixation. *Rev Inst Med Trop Sao Paulo* **58**, 3 (2016).

279. Abrantes, W., T. R. & Figueiredo, F. B. Environmental factors associated with canine visceral leishmaniasis in an area with recent introduction of the disease in the state of rio de janeiro, brazil. *Cad Saude Publica* **34**, e00021117 (2018).

280. Akhavan, Y.-E., A. & Khamesipour, A. Molecular epizootiology of rodent leishmaniasis in a hyperendemic area of iran. *Iran J Public Health* **39**, 1–7 (2010).

281. Akhtardanesh, S., B. & Pourafshar, N. G. Feline visceral leishmaniasis in kerman, southeast of iran: Serological and molecular study. *J Vector Borne Dis* **54**, 96–102 (2017).

282. Akinpelu, A. I. Prevalence and intensity of blood parasites in wild pigeons and doves (family: Columbidae) from shasha forest reserve, ile-ife, nigeria. *Asian Journal of Animal and Veterinary Advances* **3**, 109–114 (2008).

283. Akter, A., S. & Katakura, K. Molecular and serological evidence of leishmania infection in stray dogs from visceral leishmaniasis-endemic areas of bangladesh. *Am J Trop Med Hyg* **95**, 795–799 (2016).

284. Albuquerque, C., A. & Cortes, S. Evaluation of four molecular methods to detect leishmania infection in dogs. *Parasit Vectors* **10**, 57 (2017).

285. Alho, P., A. M. & Carvalho, L. M. de. Seroprevalence of vector-borne pathogens and molecular detection of borrelia afzelii in military dogs from portugal. *Parasit Vectors* **9**, 225 (2016).

286. Alroy, H., K. A. & Working Group on Chagas Disease in, P. Prevalence and transmission of trypanosoma cruzi in people of rural communities of the high jungle of northern peru. *PLoS Negl Trop Dis* **9**, e0003779 (2015).

287. Alvasen, J., K. & Emanuelson, U. A field survey on parasites and antibodies against selected pathogens in owned dogs in lilongwe, malawi. *J S Afr Vet Assoc* **87**, e1–6 (2016).

288. Leite Rabelo, T., Elida Mara & Bhering, L. L. Comparison of three methods for diagnosis of trypanosoma (duttonella) vivax in cattle. *Genetics and Molecular Research* **16**, (2017).

289. Amer, R., S. & Nakai, Y. Molecular identification and phylogenetic analysis of trypanosoma evansi from dromedary camels (camelus dromedarius) in egypt, a pilot study. *Acta Trop* **117**, 39–46 (2011).

290. Amiri Ghannat Saman, D., I. & Pirestani, M. Biodiversity, leishmania genetic typing and host identification of phlebotomine species in endemic foci of southeastern iran. *Heliyon* **5**, e02369 (2019).

291. Antonio, M., E. G. & Machado-Coelho, G. L. Canine visceral leishmaniasis in the krenak indigenous community, resplendor, minas gerais state, brazil, 2007. *Cad Saude Publica* **27**, 603–7 (2011).

292. Aragort, A., W. & Sanmartin, M. L. Blood protozoans in elasmobranchs of the family rajidae from galicia (NW spain). *Dis Aquat Organ* **65**, 63–8 (2005).

293. Arce-Fonseca, C.-S., M. & Rodriguez-Morales, O. Seropositivity for trypanosoma cruzi in domestic dogs from sonora, mexico. *Infect Dis Poverty* **6**, 120 (2017).

294. Aslantas, O., O. & Babur, C. Seroepidemiology of leptospirosis, toxoplasmosis, and leishmaniosis among dogs in ankara, turkey. *Vet Parasitol* **129**, 187–91 (2005).

295. Attipa, P., C. & Tasker, S. Prevalence study and risk factor analysis of selected bacterial, protozoal and viral, including vector-borne, pathogens in cats from cyprus. *Parasit Vectors* **10**, 130 (2017).

296. Austen, O., J. M. & Ryan, U. High prevalence of trypanosoma vegrandis in bats from western australia. *Vet Parasitol* **214**, 342–7 (2015).

297. Azeem, T., Tamoor & Umar, S. Hematobiochemical disorder in camels suffering from different hemoparasites. *Pakistan Journal of Zoology* **51**, 591–596 (2019).

298. Azpurua, D. L. C., J. & Windsor, D. Lutzomyia sand fly diversity and rates of infection by wolbachia and an exotic leishmania species on barro colorado island, panama. *PLoS Negl Trop Dis* **4**, e627 (2010).

299. Bakirci, B., Serkan & KaragenÇ, T. Molecular and seroprevalence of canine visceral leishmaniasis in west anatolia, turkey. *Turkish Journal of Veterinary and Animal Sciences* **40**, 637–644 (2016).

300. Bamorovat, S., M. & Aflatoonian, M. R. Canine visceral leishmaniasis in kerman, southeast of iran: A seroepidemiological, histopathological and molecular study. *Iran J Parasitol* **9**, 342–9 (2014).

301. Bar, M. E. & Wisnivesky-Colli, C. Triatoma sordida stal 1859 (hemiptera, reduviidae: Triatominae) in palms of northeastern argentina. *Mem Inst Oswaldo Cruz* **96**, 895–9 (2001).

302. Barbosa, A., A. & Ryan, U. First report of trypanosoma vegrandis in koalas (phascolarctos cinereus). *Parasitol Int* **65**, 316–8 (2016).

303. Barbosa, G., A. D. & Ryan, U. Increased genetic diversity and prevalence of co-infection with trypanosoma spp. In koalas (phascolarctos cinereus) and their ticks identified using next-generation sequencing (NGS). *PLoS One* **12**, e0181279 (2017).

304. Barbosa, R., A. & Ryan, U. Prevalence, genetic diversity and potential clinical impact of blood-borne and enteric protozoan parasites in native mammals from northern australia. *Vet Parasitol* **238**, 94–105 (2017).

305. Barnard, M.-H., William H. & Matsuoka, S. M. Prevalence of hematozoa infections among breeding and wintering rusty blackbirds. *The Condor* **112**, 849–853 (2010).

306. Barroso, P. *et al.* Visceral leishmaniasis caused by leishmania infantum in salta, argentina: Possible reservoirs and vectors. *The American journal of tropical medicine and hygiene* **93**, 334 (2015).

307. Ben Othman, G., S. & Ben Abderrazak, S. First detection of leishmania DNA in psammomys obesus and psammomys vexillaris: Their potential involvement in the epidemiology of leishmaniasis in tunisia. *Infect Genet Evol* **59**, 7–15 (2018).

308. Benassi, B., J. C. & Oliveira, T. Detection of leishmania infantum DNA in conjunctival swabs of cats by quantitative real-time PCR. *Exp Parasitol* **177**, 93–97 (2017).

309. Berger, W., R. & Kotler, B. P. Zoonotic disease in a peripheral population: Persistence and transmission of leishmania major in a putative sink-source system in the negev highlands, israel. *Vector Borne Zoonotic Dis* **14**, 592–600 (2014).

310. Bhattarai, D., N. R. & Van der Auwera, G. Development and evaluation of different PCR-based typing methods for discrimination of leishmania donovani isolates from nepal. *Parasitology* **137**, 947–57 (2010).

311. Bhutto, J. A. S., B.Gadahi. Field investigation on the prevalence of trypanosomiasis in camels in relation to sex, age, breed and herd size. *Pakistan Veterinary Journal* **30**, 175–177 (2010).

312. Bigeli, O., J. G. & Teles, N. M. Diagnosis of leishmania (leishmania) chagasi infection in dogs and the relationship with environmental and sanitary aspects in the municipality of palmas, state of tocantins, brazil. *Rev Soc Bras Med Trop* **45**, 18–23 (2012).

313. Bilgin, N. Y., Z.Turan. Prevalence of leishmaniosis in dogs in istanbul, turkey determined by using PCR. *Journal of the Hellenic Veterinary Medical Society* **66**, 106–112 (2015).

314. Bitew, Y. A., M.Amedie. Prevalence of bovine trypanosomosis in selected areas of jabi tehenan district, west gojam of amhara regional state, northwestern ethiopia. *African Journal of Agricultural Research* **6**, 140–144 (2011).

315. Bolukbas, G. Z. G., C. S.Pekmezci. Evidence of leishmania spp. Antibodies and DNA in dogs in the middle black sea region of turkey. *Ankara Universitesi Veteriner Fakultesi Dergisi* **63**, 111–114 (2016).

316. Borji, G. P., H.Razmi. Epidemiological study on haemoparasites of dromedary (camelus dromedarius) in iran. *Journal of Camel Practice and Research* **16**, 217–219 (2009).

317. Botto-Mahan, B., C. & Solari, A. Field assessment of trypanosoma cruzi infection and host survival in the native rodent octodon degus. *Acta Trop* **122**, 164–7 (2012).

318. Brasil, M., Arthur Willian de Lima & Azevedo, S. S. de. Prevalence and risk factors associated with leishmania spp. And trypanosoma cruzi infections in dogs presented at veterinary clinics in joão pessoa, paraíba state, northeastern brazil. *Semina: Ciências Agrárias* **39**, 2293–2300 (2018).

319. Bray, B., D. P. & Birtles, R. J. Haemoparasites of common shrews (sorex araneus) in northwest england. *Parasitology* **134**, 819–26 (2007).

320. Breniere, A., S. F. & Noireau, F. Genetic characterization of trypanosoma cruzi DTUs in wild triatoma infestans from bolivia: Predominance of TcI. *PLoS Negl Trop Dis* **6**, e1650 (2012).

321. Brigada, D., A. M. & Basso, B. American tripanosomiasis: A study on the prevalence of trypanosoma cruzi and trypanosoma cruzi-like organisms in wild rodents in san luis province, argentina. *Rev Soc Bras Med Trop* **43**, 249–53 (2010).

322. Brito, L., F. G. & Paz, G. S. Canine visceral leishmaniasis in the northeast region of brazil. *J Venom Anim Toxins Incl Trop Dis* **22**, 15 (2016).

323. Brown, R., E. L. & Yabsley, M. J. Seroprevalence of trypanosoma cruzi among eleven potential reservoir species from six states across the southern united states. *Vector Borne Zoonotic Dis* **10**, 757–63 (2010).

324. Buhaya, G., M. H. & Maldonado, R. A. Incidence of trypanosoma cruzi infection in triatomines collected at indio mountains research station. *Acta Trop* **150**, 97–9 (2015).

325. Bustamante, D. U.-S., D. M. & Pennington, P. M. Ecological, social and biological risk factors for continued trypanosoma cruzi transmission by triatoma dimidiata in guatemala. *PLoS One* **9**, e104599 (2014).

326. Cai, W., Y. & Zhang, X. First report of the prevalence and genotype of trypanosoma spp. In bats in yunnan province, southwestern china. *Acta Trop* **198**, 105105 (2019).

327. Caldart, C., Eloiza Teles & Freire, R. L. Zoonosis in dogs and cats attended by the birth control project: Toxoplasma gondii, leishmania spp. And leptospira spp., Serodiagnosis and epidemiology. *Semina: Ciências Agrárias* **36**, 253–265 (2015).

328. Caldart, F., E. T. & Navarro, I. T. Leishmania in synanthropic rodents (rattus rattus): New evidence for the urbanization of leishmania (leishmania) amazonensis. *Rev Bras Parasitol Vet* **26**, 17–27 (2017).

329. Calzada, S., J. E. & Chaves, L. F. Cutaneous leishmaniasis in dogs: Is high seroprevalence indicative of a reservoir role? *Parasitology* **142**, 1202–14 (2015).

330. Camejo, P. M. G., M. I.Aso. Relationship between asymptomatic infections with anaplasma marginale, babesia spp. And trypanosoma vivax in bulls and testosterone levels. *Revista Cientifica-Facultad De Ciencias Veterinarias* **26**, 13–19 (2016).

331. Cardoso, G., L. & Baneth, G. First report of anaplasma platys infection in red foxes (vulpes vulpes) and molecular detection of ehrlichia canis and leishmania infantum in foxes from portugal. *Parasit Vectors* **8**, 144 (2015).

332. Cardoso, L., L. & Solano-Gallego, L. Low seroprevalence of leishmania infantum infection in cats from northern portugal based on DAT and ELISA. *Vet Parasitol* **174**, 37–42 (2010).

333. Castro, B., J. C. & Barcante, J. M. P. Molecular detection of leishmania spp in lutzomyia longipalpis in the city of lavras, minas gerais, brazil. *Braz J Med Biol Res* **52**, e8224 (2019).

334. Chargui, S., N. & Babba, H. Transmission cycle analysis in a leishmania infantum focus: Infection rates and blood meal origins in sand flies (diptera: psychodidae). *J Vector Ecol* **43**, 321–327 (2018).

335. Charles, K., R. A. & Yabsley, M. J. Southern plains woodrats (neotoma micropus) from southern texas are important reservoirs of two genotypes of trypanosoma cruzi and host of a putative novel trypanosoma species. *Vector Borne Zoonotic Dis* **13**, 22–30 (2013).

336. Chatzis, A., M. K. & Saridomichelakis, M. N. Cytological and molecular detection of leishmania infantum in different tissues of clinically normal and sick cats. *Vet Parasitol* **202**, 217–25 (2014).

337. Chaudhary, Z. I. & Iqbal, J. Incidence, biochemical and haematological alterations induced by natural trypanosomosis in racing dromedary camels. *Acta Trop* **77**, 209–13 (2000).

338. Chaudhry, A. A., Z. I.JahanzaibAslam. Prevalence of trypanosoma evansi in camels through polymerase chain reaction and haematocrit centrifugation technique in punjab (pakistan). *Journal of Camel Practice and Research* **15**, 183–185 (2008).

339. Chen, L., H. & Chen, J. Multi-locus characterization and phylogenetic inference of leishmania spp. In snakes from northwest china. *PLoS One* **14**, e0210681 (2019).

340. Cherenet, S., T. & Van den Bossche, P. A comparative longitudinal study of bovine trypanosomiasis in tsetse-free and tsetse-infested zones of the amhara region, northwest ethiopia. *Vet Parasitol* **140**, 251–8 (2006).

341. Chitimia, M.-G., L. & Berriatua, E. Cryptic leishmaniosis by leishmania infantum, a feature of canines only? A study of natural infection in wild rabbits, humans and dogs in southeastern spain. *Vet Parasitol* **181**, 12–6 (2011).

342. Concannon, W.-O., R. & Birtles, R. J. Molecular characterization of haemoparasites infecting bats (microchiroptera) in cornwall, UK. *Parasitology* **131**, 489–96 (2005).

343. Constantino, P., Caroline & Navarro, I. T. Seroepidemiology of leishmania spp. In dogs residing in telêmaco borba, paraná, brazil. *Semina: Ciências Agrárias* **35**, 3181–3190 (2014).

344. Coronado, R., X. & Solari, A. Molecular epidemiology of chagas disease in the wild transmission cycle: The evaluation in the sylvatic vector mepraia spinolai from an endemic area of chile. *Am J Trop Med Hyg* **81**, 656–9 (2009).

345. Cortada, D., V. M. & Goncalves da Costa, S. C. Canine visceral leishmaniosis in anastacio, mato grosso do sul state, brazil. *Vet Res Commun* **28**, 365–74 (2004).

346. Cortez, V., A. P. & Teixeira, M. M. The taxonomic and phylogenetic relationships of trypanosoma vivax from south america and africa. *Parasitology* **133**, 159–69 (2006).

347. Coulibaly, T., C. A. & Doumbia, S. Impact of insecticide-treated bednets and indoor residual spraying in controlling populations of phlebotomus duboscqi, the vector of leishmania major in central mali. *Parasit Vectors* **11**, 345 (2018).

348. Couto, L., C. G. & Ruano, R. Serological study of selected vector-borne diseases in shelter dogs in central spain using point-of-care assays. *Vector Borne Zoonotic Dis* **10**, 885–8 (2010).

349. Cuba, A.-F., C. A. & Miles, M. A. The triatomines of northern peru, with emphasis on the ecology and infection by trypanosomes of rhodnius ecuadoriensis (triatominae). *Mem Inst Oswaldo Cruz* **97**, 175–83 (2002).

350. Curi, M., N. H. & Talamoni, S. A. Serologic evidence of leishmania infection in free-ranging wild and domestic canids around a brazilian national park. *Mem Inst Oswaldo Cruz* **101**, 99–101 (2006).

351. Curtis-Robles, Z., R. & Hamer, S. A. Trypanosoma cruzi (agent of chagas disease) in sympatric human and dog populations in "colonias" of the lower rio grande valley of texas. *Am J Trop Med Hyg* **96**, 805–814 (2017).

352. Costa, C., Letícia Da & Freire, R. L. Leishmaniasis in dogs from recycling centers and from a neighborhood with adjacent forest in an urban area of londrina, paraná, brazil. *Semina: Ciências Agrárias* **37**, 1407–1414 (2016).

353. Dantas-Torres, de P.-C., F. & Brandao-Filho, S. P. Cutaneous and visceral leishmaniasis in dogs from a rural community in northeastern brazil. *Vet Parasitol* **170**, 313–7 (2010).

354. Dario, L., M. A. & Jansen, A. M. High trypanosoma spp. Diversity is maintained by bats and triatomines in espirito santo state, brazil. *PLoS One* **12**, e0188412 (2017).

355. Davami, M., M. H. & Pourahmad, M. Molecular survey on detection of leishmania infection in rodent reservoirs in jahrom district, southern iran. *J Arthropod Borne Dis* **8**, 139–46 (2014).

356. Dayo, B., G. K. & Thevenon, S. Prevalence and incidence of bovine trypanosomosis in an agro-pastoral area of southwestern burkina faso. *Res Vet Sci* **88**, 470–7 (2010).

357. Curi, P., N. H. & Chiarello, A. G. Factors associated with the seroprevalence of leishmaniasis in dogs living around atlantic forest fragments. *PLoS One* **9**, e104003 (2014).

358. Carvalho, V. de, M. R. & Brandao Filho, S. P. Natural leishmania infantum infection in migonemyia migonei (franca, 1920) (diptera:psychodidae:phlebotominae) the putative vector of visceral leishmaniasis in pernambuco state, brazil. *Acta Trop* **116**, 108–10 (2010).

359. Lima, G., J. T. & Soares, R. M. Serodiagnosis of visceral and cutaneous leishmaniasis in human and canine populations living in indigenous reserves in the brazilian amazon region. *Rev Soc Bras Med Trop* **50**, 61–66 (2017).

360. Mendonca, B. de, I. L. & Alves, L. C. Leishmania (infantum) chagasi in canine urinary sediment. *Rev Bras Parasitol Vet* **24**, 92–4 (2015).

361. Oliveira, C., E. F. & Galati, E. A. Leishmania amazonensis DNA in wild females of lutzomyia cruzi (diptera: Psychodidae) in the state of mato grosso do sul, brazil. *Mem Inst Oswaldo Cruz* **110**, 1051–7 (2015).

362. Oliveira, A., L. C. & Mendonca-Lima, F. W. Seroprevalence and risk factors for canine visceral leishmaniasis in the endemic area of dias d’avila, state of bahia, brazil. *Rev Soc Bras Med Trop* **43**, 400–4 (2010).

363. Oliveira, G., Patrícia Magalhães de & Saut, J. P. E. Seroepidemiology ofLeishmaniaspp. In equids from uberlândia, minas gerais, brazil. *Ciência Rural* **47**, (2017).

364. Padua, I. de, S. B. & Pilarski, F. First record of trypanosoma sp. (Protozoa: Kinetoplastida) in tuvira (gymnotus aff. Inaequilabiatus) in the pantanal wetland, mato grosso do sul state, brazil. *Rev Bras Parasitol Vet* **20**, 85–7 (2011).

365. Seabra, P. de, N. M. & Oliveira, T. M. Toxoplasma gondii, neospora caninum and leishmania spp. Serology and leishmania spp. PCR in dogs from pirassununga, SP. *Rev Bras Parasitol Vet* **24**, 454–8 (2015).

366. Sousa, H., K. C. & Andre, M. R. Serological detection of toxoplasma gondii, leishmania infantum and neospora caninum in cats from an area endemic for leishmaniasis in brazil. *Rev Bras Parasitol Vet* **23**, 449–55 (2014).

367. Deiró, M., Ana Graziela De Jesus & Albuquerque, G. R. Antibody occurrence of anti-toxoplasma gondii, leishmania sp. And ehrlichia canis in dogs in bahia state. *Semina: Ciências Agrárias* **39**, 199–210 (2018).

368. Demir, S. & Karakus, M. Natural leishmania infection of phlebotomus sergenti (diptera: Phlebotominae) in an endemic focus of cutaneous leishmaniasis in sanliurfa, turkey. *Acta Trop* **149**, 45–8 (2015).

369. Deviche, G., P. & Manteca, X. Interspecific variability of prevalence in blood parasites of adult passerine birds during the breeding season in alaska. *J Wildl Dis* **37**, 28–35 (2001).

370. Dia, M. L. Parasites of the camel in burkina faso. *Trop Anim Health Prod* **38**, 17–21 (2006).

371. Diakou, P., A. & Lazarides, K. Specific anti-leishmania spp. Antibodies in stray cats in greece. *J Feline Med Surg* **11**, 728–30 (2009).

372. Diaz-Saez, M.-E., V. & Martin-Sanchez, J. High rates of leishmania infantum and trypanosoma nabiasi infection in wild rabbits (oryctolagus cuniculus) in sympatric and syntrophic conditions in an endemic canine leishmaniasis area: Epidemiological consequences. *Vet Parasitol* **202**, 119–27 (2014).

373. Dogan, O., N. & Bor, O. Sero-epidemological survey on canine visceral leishmaniasis and the distribution of sandfly vectors in northwestern turkey: Prevention strategies for childhood visceral leishmaniasis. *J Trop Pediatr* **52**, 212–7 (2006).

374. Dos Santos, L., F. C. B. & Jansen, A. M. Trypanosoma sp. Diversity in amazonian bats (chiroptera; mammalia) from acre state, brazil. *Parasitology* **145**, 828–837 (2018).

375. Duarte, C., A. & Vaz, Y. Survey of infectious and parasitic diseases in stray cats at the lisbon metropolitan area, portugal. *J Feline Med Surg* **12**, 441–6 (2010).

376. Dumitrache, N.-B., M. O. & Baneth, G. The quest for canine leishmaniasis in romania: The presence of an autochthonous focus with subclinical infections in an area where disease occurred. *Parasit Vectors* **9**, 297 (2016).

377. Durrani, B., A. Z. & Azeem, M. Use of physiological biomarkers in diagnosis along with field trials of different trypanisidal drugs in camels of cholistan desert. *Microb Pathog* **108**, 1–5 (2017).

378. Ebani, P., V. V. & Mancianti, F. Serological survey on some pathogens in wild brown hares (lepus europaeus) in central italy. *Asian Pac J Trop Med* **9**, 465–9 (2016).

379. Echchakery, C., M. & Boumezzough, A. Molecular detection of leishmania infantum and leishmania tropica in rodent species from endemic cutaneous leishmaniasis areas in morocco. *Parasit Vectors* **10**, 454 (2017).

380. Echeverria, S., J. T. & Borges, F. A. Clinical and therapeutic aspects of an outbreak of canine trypanosomiasis. *Rev Bras Parasitol Vet* **28**, 320–324 (2019).

381. Elhaig, M. M. & Sallam, N. H. Molecular survey and characterization of trypanosoma evansi in naturally infected camels with suspicion of a trypanozoon infection in horses by molecular detection in egypt. *Microb Pathog* **123**, 201–205 (2018).

382. Estrada-Franco, B., J. G. & Garg, N. Human trypanosoma cruzi infection and seropositivity in dogs, mexico. *Emerg Infect Dis* **12**, 624–30 (2006).

383. Faye, B. *et al.* Canine visceral leishmaniasis caused by leishmania infantum in senegal: Risk of emergence in humans? *Microbes and infection / Institut Pasteur* **12**, 1219–25 (2010).

384. Faye, P. de A., D. & Geerts, S. Prevalence and incidence of trypanosomosis in horses and donkeys in the gambia. *Vet Parasitol* **101**, 101–14 (2001).

385. Fereig, M., R. M. & Nishikawa, Y. Seroprevalence of babesia bovis, b. Bigemina, trypanosoma evansi, and anaplasma marginale antibodies in cattle in southern egypt. *Ticks Tick Borne Dis* **8**, 125–131 (2017).

386. Ferreira Ede, C., C. & Gontijo, C. M. Mixed infection of leishmania infantum and leishmania braziliensis in rodents from endemic urban area of the new world. *BMC Vet Res* **11**, 71 (2015).

387. Castro Ferreira, P. de, E. & Gontijo, C. M. F. Leishmania (v.) Braziliensis infecting bats from pantanal wetland, brazil: First records for platyrrhinus lineatus and artibeus planirostris. *Acta Trop* **172**, 217–222 (2017).

388. Ferreira, C., R. T. B. & Moreira, O. C. Detection and genotyping of trypanosoma cruzi from acai products commercialized in rio de janeiro and para, brazil. *Parasit Vectors* **11**, 233 (2018).

389. Figueiredo, M., F. B. & Schubach, T. M. Efficacy of an indirect immunofluorescence test in the diagnosis of canine leishmaniosis. *Vet J* **186**, 123–4 (2010).

390. Figueiredo, L. D. de V., F. B.Nascimento. Serological diagnosis of feline tegumentary leishmaniasis by indirect immunofluorescence (IFI) and enzyme-linked immunosorbent assay (ELISA) in an endemic area in brazil. *Acta Scientiae Veterinariae* **44**, (2016).

391. Fikru, A., R. & Buscher, P. Trypanosome infection in dromedary camels in eastern ethiopia: Prevalence, relative performance of diagnostic tools and host related risk factors. *Vet Parasitol* **211**, 175–81 (2015).

392. Silva Filho, T., Mauro de F. & Navarro, I. T. Infection by toxoplasma gondii and leishmania spp. In humans and dogs from rural settlements in northern paraná state, brazil. *Semina: Ciências Agrárias* **33**, 3251–3264 (2012).

393. Freitas, S., Y. B. N. & Amora, S. S. A. Natural infection by trypanosoma cruzi in triatomines and seropositivity for chagas disease of dogs in rural areas of rio grande do norte, brazil. *Rev Soc Bras Med Trop* **51**, 190–197 (2018).

394. Galaviz-Silva, M.-H., L. & Molina-Garza, Z. J. Prevalence of trypanosoma cruzi infection in dogs and small mammals in nuevo leon, mexico. *Rev Argent Microbiol* **49**, 216–223 (2017).

395. Garcia, M., N. & Dominguez, M. Evidence of leishmania infantum infection in rabbits (oryctolagus cuniculus) in a natural area in madrid, spain. *Biomed Res Int* **2014**, 318254 (2014).

396. Gari, A., F. R. & Claes, F. Comparative diagnosis of parasitological, serological, and molecular tests in dourine-suspected horses. *Trop Anim Health Prod* **42**, 1649–54 (2010).

397. Garvin, M., M. C. & Crain, S. K. Prevalence of hematozoa in overwintering american redstarts (setophaga ruticilla): No evidence for local transmission. *J Wildl Dis* **40**, 115–8 (2004).

398. Gavgani, M., A. S. & Davies, C. R. Domestic dog ownership in iran is a risk factor for human infection with leishmania infantum. *Am J Trop Med Hyg* **67**, 511–5 (2002).

399. Ghaemi, Z., M. & Jannati Pirouz, H. Evaluation of trypanosama evansi prevalence and risk factors in the one-humped camels (camelus dromedarius) of the north-east of iran by a real-time PCR test. *Prev Vet Med* **168**, 60–65 (2019).

400. Giannakopoulos, C. N. P., A.Tsokana. Molecular investigation and geographical distribution of leishmania spp infection in stray and owned cats (felis catus) in thessaly, central greece. *Journal of the Hellenic Veterinary Medical Society* **68**, 27–34 (2017).

401. Gicheru, J., M. M. & Suleman, M. A. Prevalence of antibodies and cell mediated immune response against leishmania major in feral nonhuman primates from kenya. *Acta Trop* **109**, 136–40 (2009).

402. Gontijo, da S., C. M. & Melo, M. N. Epidemiological studies of an outbreak of cutaneous leishmaniasis in the rio jequitinhonha valley, minas gerais, brazil. *Acta Trop* **81**, 143–50 (2002).

403. Grisard, C.-P., E. C. & Steindel, M. Trypanosoma cruzi infection in didelphis marsupialis in santa catarina and arvoredo islands, southern brazil. *Mem Inst Oswaldo Cruz* **95**, 795–800 (2000).

404. Guimaraes, B., K. S. & Abreu-Silva, A. L. Canine visceral leishmaniasis in sao jose de ribamar, maranhao state, brazil. *Vet Parasitol* **131**, 305–9 (2005).

405. Gunasekara, S., E. & Yokoyama, N. Epidemiological survey of hemoprotozoan parasites in cattle from low-country wet zone in sri lanka. *Parasitol Int* **71**, 5–10 (2019).

406. Haas, L., M. & Hrehova, Z. Occurrence of blood parasites and intensity of infection in prunella modularis in the montane and subalpine zone in the slovak carpathians. *Acta Parasitol* **57**, 221–7 (2012).

407. Haenen, L., O. L. M. & Breteler, J. K. The health status of european silver eels, anguilla anguilla, in the dutch river rhine watershed and lake IJsselmeer. *Aquaculture* **309**, 15–24 (2010).

408. Haji, M., I. J. & Namangala, B. Occurrence of haemoparasites in cattle in monduli district, northern tanzania. *Onderstepoort J Vet Res* **81**, (2014).

409. Hamad, F., I. & Bittar, F. Wild gorillas as a potential reservoir of leishmania major. *J Infect Dis* **211**, 267–73 (2015).

410. Hamarsheh, N., O. & Al-Jawabreh, A. Serological and molecular survey of leishmania parasites in apparently healthy dogs in the west bank, palestine. *Parasit Vectors* **5**, 183 (2012).

411. Hamill, K., L. C. & Picozzi, K. Domestic pigs as potential reservoirs of human and animal trypanosomiasis in northern tanzania. *Parasit Vectors* **6**, 322 (2013).

412. Hanel, D., J. & Votypka, J. Blood parasites in northern goshawk (accipiter gentilis) with an emphasis to leucocytozoon toddi. *Parasitol Res* **115**, 263–70 (2016).

413. Hassan, O., M. M. & Elnaiem, D. E. Role of the domestic dog as a reservoir host of leishmania donovani in eastern sudan. *Parasit Vectors* **2**, 26 (2009).

414. Hernandez, S., C. & Ramirez, J. D. Untangling the transmission dynamics of primary and secondary vectors of trypanosoma cruzi in colombia: Parasite infection, feeding sources and discrete typing units. *Parasit Vectors* **9**, 620 (2016).

415. Herrera, L., C. P. & Wesson, D. M. Genotype diversity of trypanosoma cruzi in small rodents and triatoma sanguisuga from a rural area in new orleans, louisiana. *Parasit Vectors* **8**, 123 (2015).

416. Hodo, R., C. L. & Hamer, S. A. Repeated cross-sectional study of trypanosoma cruzi in shelter dogs in texas, in the context of dirofilaria immitis and tick-borne pathogen prevalence. *J Vet Intern Med* **33**, 158–166 (2019).

417. Hofmann, H., M. & Joachim, A. Vector-borne pathogens affecting shelter dogs in eastern crete, greece. *Parasitol Res* **118**, 1661–1666 (2019).

418. Holland, T., W. G. & Vercruysse, J. Prevalence of trypanosoma evansi in water buffaloes in remote areas in northern vietnam using PCR and serological methods. *Trop Anim Health Prod* **36**, 45–8 (2004).

419. Holmstad, A., P. R. & Skorping, A. Standard sampling techniques underestimate prevalence of avian hematozoa in willow ptarmigan (lagopus lagopus). *J Wildl Dis* **39**, 354–8 (2003).

420. Hussain, Z. G., M.Saeed. Molecular detection and seasonal prevalence of trypanosoma brucei and its effect on hematobiochemical parameters in donkeys from dera ghazi khan district in southern punjab, pakistan. *Pakistan Journal of Zoology* **48**, 1781–1786 (2016).

421. Hussain, K., R. & Mehmood, K. Clinico-hematological and oxidative stress status in nili ravi buffaloes infected with trypanosoma evansi. *Microb Pathog* **123**, 126–131 (2018).

422. Jafari, N., R. & Parvizi, P. Molecular characterization of sandflies and leishmania detection in main vector of zoonotic cutaneous leishmaniasis in abarkouh district of yazd province, iran. *Asian Pac J Trop Med* **6**, 792–7 (2013).

423. Jakes, O., K. A. & Adlard, R. Hemoprotozoa of freshwater turtles in queensland. *J Wildl Dis* **37**, 12–9 (2001).

424. Jing, M., Z. & Inoue, N. A field study to estimate the prevalence of bovine african trypanosomosis in butaleja district, uganda. *J Vet Med Sci* **71**, 525–7 (2009).

425. Karakus, A., M. & Ozbel, Y. Vector and reservoir surveillance study in a canine and human leishmaniasis endemic area in most western part of turkey, karaburun. *Acta Trop* **190**, 177–182 (2019).

426. Karbowiak, R., Grzegorz & Wita, I. Natural infections of small mammals with blood parasites on the borderland of boreal and temperate forest zones. *Acta Theriologica* **50**, 31–42 (2005).

427. Karimuribo, M., E. D. & Ballingall, K. T. Analysis of host genetic factors influencing african trypanosome species infection in a cohort of tanzanian bos indicus cattle. *Vet Parasitol* **179**, 35–42 (2011).

428. Kassa, T. C., T.Eguale. Prevalence of camel trypanosomosis and its vectors in fentale district, south east shoa zone, ethiopia. *Veterinarski Arhiv* **81**, 611–621 (2011).

429. Kato, U., H. & Hashiguchi, Y. Establishment of a mass screening method of sand fly vectors for leishmania infection by molecular biological methods. *Am J Trop Med Hyg* **77**, 324–9 (2007).

430. Keck, J., N.Dereuer. Epidemiology of canine leishmaniasis by cross-sectional study in the french focus of cevennes. *Revue De Medecine Veterinaire* **154**, 599–604 (2003).

431. Kelly, B., E. & Lymbery, A. J. Haematozoa of wild catfishes in northern australia. *Int J Parasitol Parasites Wildl* **7**, 12–17 (2018).

432. Kenubih, D., A. & Lemma, W. Preliminary survey of domestic animal visceral leishmaniasis and risk factors in north-west ethiopia. *Trop Med Int Health* **20**, 205–10 (2015).

433. Khanmohammadi, E. R., M.Fallah. Study on seroprevalence of canine visceral leishmaniasis (CVL) in ownership dogs of sarab, east azerbaijan, province, northwest of iran with indirect immuno fluorescence antibody test (IFAT) and its health importance in 2008-2009. *Journal of Animal and Veterinary Advances* **9**, 139–143 (2010).

434. Kilic, C. O., S.Babur. Investigation of anti-toxoplasma gondii and anti-leishmania infantum antibodies among sivas kangal dogs. *Turkish Journal of Veterinary & Animal Sciences* **32**, 299–304 (2008).

435. Kovalenko, R., D. A. & Baneth, G. Canine leishmaniosis and its relationship to human visceral leishmaniasis in eastern uzbekistan. *Parasit Vectors* **4**, 58 (2011).

436. Kubacka, G., J. & Podmokla, E. Correlates of blood parasitism in a threatened marshland passerine: Infection by kinetoplastids of the genus trypanosoma is related to landscape metrics of habitat edge. *Parasitology* **146**, 1036–1046 (2019).

437. Laohasinnarong, T., D. & Inoue, N. Prevalence of trypanosoma sp. In cattle from tanzania estimated by conventional PCR and loop-mediated isothermal amplification (LAMP). *Parasitol Res* **109**, 1735–9 (2011).

438. Laranjeira, M., D. F. & Laurenti, M. D. Serological and infection statuses of dogs from a visceral leishmaniasis-endemic area. *Rev Saude Publica* **48**, 563–71 (2014).

439. Leca Junior, G., N. F. & Silva, F. L. Epidemiology of canine leishmaniasis in southern bahia, brazil. *Acta Trop* **148**, 115–9 (2015).

440. Lemma, B., W. & Hailu, A. Preliminary study on investigation of zoonotic visceral leishmaniasis in endemic foci of ethiopia by detecting leishmania infections in rodents. *Asian Pac J Trop Med* **10**, 418–422 (2017).

441. Leontides, S., L. S. & Mylonakis, M. E. A cross-sectional study of leishmania spp. Infection in clinically healthy dogs with polymerase chain reaction and serology in greece. *Vet Parasitol* **109**, 19–27 (2002).

442. Leppert, D., L. L. & Kaltenecker, G. S. Survey of blood parasites in two forest owls, northern saw-whet owls and flammulated owls, of western north america. *J Wildl Dis* **44**, 475–9 (2008).

443. Lima, D.-T., B. S. & Brandao-Filho, S. P. Small mammals as hosts of leishmania spp. In a highly endemic area for zoonotic leishmaniasis in north-eastern brazil. *Trans R Soc Trop Med Hyg* **107**, 592–7 (2013).

444. Lima-Oliveira, F., T. M. & Almeida, C. E. Molecular eco-epidemiology on the sympatric chagas disease vectors triatoma brasiliensis and triatoma petrocchiae: Ecotopes, genetic variation, natural infection prevalence by trypanosomatids and parasite genotyping. *Acta Trop* **201**, 105188 (2020).

445. Lins, S., T. N. B. & Ramos, R. A. N. Seroprevalence and spatial distribution of canine leishmaniasis in an endemic region in brazil: How has the situation changed after 10 years? *Rev Soc Bras Med Trop* **51**, 680–682 (2018).

446. Lisi, D., O. & Maroli, M. Persistence of phlebotomine leishmania vectors in urban sites of catania (sicily, italy). *Parasit Vectors* **7**, 560 (2014).

447. Lisulo, S., M. & Namangala, B. Determination of the prevalence of african trypanosome species in indigenous dogs of mambwe district, eastern zambia, by loop-mediated isothermal amplification. *Parasit Vectors* **7**, 19 (2014).

448. Lopes, S., A. P. & Cordeiro-da-Silva, A. Prevalence of antibodies to leishmania infantum and toxoplasma gondii in horses from the north of portugal. *Parasit Vectors* **6**, 178 (2013).

449. Lopes, S. E., P. M. & Sousa, V. R. Seroprevalence and risk factors associated with visceral leishmaniasis in dogs in jaciara, state of mato grosso. *Rev Soc Bras Med Trop* **47**, 791–5 (2014).

450. Maganga, M., G. D. & Ollomo, B. Molecular identification of trypanosome species in trypanotolerant cattle from the south of gabon. *Parasite* **24**, 4 (2017).

451. Maharjan, D. R., M.Mishra. Impact of emerging zoonotic diseases on animal health. **1081**, 320–321 (2006).

452. Maia, A., C. & Roura, X. Molecular detection of leishmania infantum, filariae and wolbachia spp. In dogs from southern portugal. *Parasit Vectors* **9**, 170 (2016).

453. Maia, C., C. & Campino, L. Serological investigation of leishmania infantum, dirofilaria immitis and angiostrongylus vasorum in dogs from southern portugal. *Parasit Vectors* **8**, 152 (2015).

454. Maia, G., C. & Campino, L. Feline leishmania infection in a canine leishmaniasis endemic region, portugal. *Vet Parasitol* **174**, 336–40 (2010).

455. Maia, R., C. & Campino, L. Prevalence of dirofilaria immitis antigen and antibodies to leishmania infantum in cats from southern portugal. *Parasitol Int* **64**, 154–6 (2015).

456. Malele, M., II & Kimbita, E. N. Multiple trypanosoma infections are common amongst glossina species in the new farming areas of rufiji district, tanzania. *Parasit Vectors* **4**, 217 (2011).

457. Foglia Manzillo, G., V. & Oliva, G. Serological and entomological survey of canine leishmaniasis in lampedusa island, italy. *BMC Vet Res* **14**, 286 (2018).

458. Marcelino, F., A. P. & Gontijo, C. M. Molecular detection of leishmania braziliensis in rattus norvegicus in an area endemic for cutaneous leishmaniasis in brazil. *Vet Parasitol* **183**, 54–8 (2011).

459. Martinez-Sanchez, C., A. & Alejandre-Aguilar, R. Effect of ectoparasitic pimeliaphilus plumifer mites (acari: Pterygosomatidae) on meccus pallidipennis (hemiptera: Reduviidae) and several other chagas’ disease vectors under laboratory conditions. *Exp Appl Acarol* **42**, 139–49 (2007).

460. Matete, G. O. Occurrence, clinical manifestation and the epidemiological implications of naturally occurring canine trypanosomosis in western kenya. *Onderstepoort J Vet Res* **70**, 317–23 (2003).

461. Mattioli, F., R. C. & Jaitner, J. Estimation of trypanosomal status by the buffy coat technique and an antibody ELISA for assessment of the impact of trypanosomosis on health and productivity of n’dama cattle in the gambia. *Vet Parasitol* **95**, 25–35 (2001).

462. Maziero, T.-S., N. & Nascimento, A. J. Rural-urban focus of canine visceral leishmaniosis in the far western region of santa catarina state, brazil. *Vet Parasitol* **205**, 92–5 (2014).

463. Medkour, D., H. & Mediannikov, O. Potential animal reservoirs (dogs and bats) of human visceral leishmaniasis due to leishmania infantum in french guiana. *PLoS Negl Trop Dis* **13**, e0007456 (2019).

464. Mekuria, A. R., S.Eyob. A cross-sectional study of equine trypanosomosis and its vectors in wolayta zone, southern ethiopia. *Journal of Animal and Veterinary Advances* **9**, 2061–2066 (2010).

465. Melo, T.-N., S. N. & Belo, V. S. Prevalence of visceral leishmaniasis in a population of free-roaming dogs as determined by multiple sampling efforts: A longitudinal study analyzing the effectiveness of euthanasia. *Prev Vet Med* **161**, 19–24 (2018).

466. Millan, J. Molecular investigation of vector-borne parasites in wild micromammals, barcelona (spain). *Parasitol Res* **117**, 3015–3018 (2018).

467. Miranda, R., J. C. & Barral, A. Frequency of infection of lutzomyia phlebotomines with leishmania braziliensis in a brazilian endemic area as assessed by pinpoint capture and polymerase chain reaction. *Mem Inst Oswaldo Cruz* **97**, 185–8 (2002).

468. Miro, H., G. & Pedraza-Diaz, S. First description of naturally acquired tritrichomonas foetus infection in a persian cattery in spain. *Parasitol Res* **109**, 1151–4 (2011).

469. Mirzaei, R., A. & Parvizi, P. Isolation and detection of leishmania species among naturally infected rhombomis opimus, a reservoir host of zoonotic cutaneous leishmaniasis in turkemen sahara, north east of iran. *Exp Parasitol* **129**, 375–80 (2011).

470. Mohammad, B., K. N. & Zainal-Abidin, A. H. Protozoan parasites of four species of wild anurans from a local zoo in malaysia. *Trop Biomed* **30**, 615–20 (2013).

471. Montenegro, J., V. M. & Zeledon, R. Chagas disease in dogs from endemic areas of costa rica. *Mem Inst Oswaldo Cruz* **97**, 491–4 (2002).

472. Montoya, G., A. & Miro, G. Implications of zoonotic and vector-borne parasites to free-roaming cats in central spain. *Vet Parasitol* **251**, 125–130 (2018).

473. Morales, M., E. A. & Lescano, A. G. Prevalence of trypanosoma cruzi and other trypanosomatids in frequently-hunted wild mammals from the peruvian amazon. *Am J Trop Med Hyg* **97**, 1482–1485 (2017).

474. Morelli, C., S. & Traversa, D. Exposure of client-owned cats to zoonotic vector-borne pathogens: Clinic-pathological alterations and infection risk analysis. *Comp Immunol Microbiol Infect Dis* **66**, 101344 (2019).

475. Morganti, V., G. & Gramiccia, M. Emerging feline vector-borne pathogens in italy. *Parasit Vectors* **12**, 193 (2019).

476. Moudy, M., R. M. & Wesson, D. M. Factors associated with peridomestic triatoma sanguisuga (hemiptera: Reduviidae) presence in southeastern louisiana. *J Med Entomol* **51**, 1043–50 (2014).

477. Moya, G., S. L. & Liotta, D. J. Leishmania infantum DNA detected in phlebotomine species from puerto iguazu city, misiones province, argentina. *Acta Trop* **172**, 122–124 (2017).

478. Murphy, M., N. & Miles, M. A. Lineage-specific rapid diagnostic tests can resolve trypanosoma cruzi TcII/v/VI ecological and epidemiological associations in the argentine chaco. *Parasit Vectors* **12**, 424 (2019).

479. Nadeem, A. C., A.Aslam. Indirect fluorescent antibody technique based prevalence of surra in equines. *Pakistan Veterinary Journal* **31**, 169–170 (2011).

480. Nair, R., A. S. & Ghosh, S. Haemoprotozoa of cattle in northern kerala, india. *Trop Biomed* **28**, 68–75 (2011).

481. Nardoni, A., S. & Mancianti, F. Serological and molecular findings of leishmania infection in healthy donkeys (equus asinus) from a canine leishmaniosis endemic focus in tuscany, italy: A preliminary report. *Pathogens* **8**, (2019).

482. Navea-Perez, D.-S., H. M. & Martin-Sanchez, J. Leishmania infantum in wild rodents: Reservoirs or just irrelevant incidental hosts? *Parasitol Res* **114**, 2363–70 (2015).

483. Ngomtcho, W., S. C. H. & Achukwi, M. D. Molecular screening of tsetse flies and cattle reveal different trypanosoma species including t. Grayi and t. Theileri in northern cameroon. *Parasit Vectors* **10**, 631 (2017).

484. Nguyen, M., T. T. & Inoue, N. Application of crude and recombinant ELISAs and immunochromatographic test for serodiagnosis of animal trypanosomosis in the umkhanyakude district of KwaZulu-natal province, south africa. *J Vet Med Sci* **77**, 217–20 (2015).

485. Nunes, L., J. B. & Marques, M. J. Leishmania infantum INFECTION IN DOGS FROM THE SOUTHERN REGION OF MINAS GERAIS STATE, BRAZIL. *Rev Inst Med Trop Sao Paulo* **58**, 75 (2016).

486. Nurcahyo, Y., W. & Prastowo, J. The prevalence of horse trypanosomiasis in sumba island, indonesia and its detection using card agglutination tests. *Vet World* **12**, 646–652 (2019).

487. Odeniran, M., P. O. & Welburn, S. C. Molecular identification of bloodmeal sources and trypanosomes in glossina spp., Tabanus spp. And stomoxys spp. Trapped on cattle farm settlements in southwest nigeria. *Med Vet Entomol* **33**, 269–281 (2019).

488. Odongo, D., S. & Magez, S. Comparative evaluation of the nested ITS PCR against the 18S PCR-RFLP in a survey of bovine trypanosomiasis in kwale county, kenya. *J Vet Diagn Invest* **28**, 589–94 (2016).

489. Oleaga, Z., A. & Ferroglio, E. Leishmania in wolves in northern spain: A spreading zoonosis evidenced by wildlife sanitary surveillance. *Vet Parasitol* **255**, 26–31 (2018).

490. Oliveira, P., F. S. & Pacheco, R. S. PCR-based diagnosis for detection of leishmania in skin and blood of rodents from an endemic area of cutaneous and visceral leishmaniasis in brazil. *Vet Parasitol* **129**, 219–27 (2005).

491. Oliveira, G., T. N. & Silva, F. L. Diagnosis and epidemiology of canine leishmaniasis in southeastern bahia, brazil. *Genet Mol Res* **15**, (2016).

492. Orozco, E., M. M. & Gurtler, R. E. A comparative study of trypanosoma cruzi infection in sylvatic mammals from a protected and a disturbed area in the argentine chaco. *Acta Trop* **155**, 34–42 (2016).

493. Padilla, M., A. M. & Basombrio, M. A. Canine infection and the possible role of dogs in the transmission of american tegumentary leishmaniosis in salta, argentina. *Vet Parasitol* **110**, 1–10 (2002).

494. Palangar, H. K., M.Jamali. Molecular study of cutaneous leishmaniasis human reservoirs and infections in bastak. *Iioab Journal* **8**, 33–38 (2017).

495. Panti-May, R., J. A. & Costa, F. A survey of zoonotic pathogens carried by house mouse and black rat populations in yucatan, mexico. *Epidemiol Infect* **145**, 2287–2295 (2017).

496. Paoletta, L. A., M. S. & Wilkowsky, S. E. Epidemiology of babesia, anaplasma and trypanosoma species using a new expanded reverse line blot hybridization assay. *Ticks Tick Borne Dis* **9**, 155–163 (2018).

497. Parrado, R., R. & Garcia, A. L. Prevalence of leishmania spp. Infection in domestic dogs in chapare, bolivia. *Vet Parasitol* **177**, 171–4 (2011).

498. Parvizi, A., P. & Baghban, N. Occurrence of low density of leishmania infantum in sandflies from a new focus of visceral leishmaniasis in northwest of iran. *J Vector Borne Dis* **50**, 127–32 (2013).

499. Pasa, T. V., S. & Ozbel, Y. Detection of leishmania major and leishmania tropica in domestic cats in the ege region of turkey. *Vet Parasitol* **212**, 389–92 (2015).

500. Pasos-Pinto, L. S.-M., S.Sanchez-Garcia. Genetic diversity and prevalence of leishmania mexicana in bichromomyia olmeca olmeca(1) in an endemic area of mexico. *Southwestern Entomologist* **42**, 983–994 (2017).

501. Perez, F., T. D. & Coura, J. R. Prevalence of american trypanosomiasis and leishmaniases in domestic dogs in a rural area of the municipality of sao joao do piaui, piaui state, brazil. *Rev Inst Med Trop Sao Paulo* **58**, 79 (2016).

502. Piantedosi, V., D. & Gramiccia, M. Epidemiological survey on leishmania infection in red foxes (vulpes vulpes) and hunting dogs sharing the same rural area in southern italy. *Acta Parasitol* **61**, 769–775 (2016).

503. Pineda, S., V. & Calzada, J. E. Prevalence of trypanosome infections in dogs from chagas disease endemic regions in panama, central america. *Vet Parasitol* **178**, 360–3 (2011).

504. Pinto, O.-M., C. M. & Grijalva, M. J. Infection by trypanosomes in marsupials and rodents associated with human dwellings in ecuador. *J Parasitol* **92**, 1251–5 (2006).

505. Pinto, O.-M., C. M. & Perkins, S. L. Bats, trypanosomes, and triatomines in ecuador: New insights into the diversity, transmission, and origins of trypanosoma cruzi and chagas disease. *PLoS One* **10**, e0139999 (2015).

506. Qiu, K., Y. & Sugimoto, C. Molecular characterization and phylogenetic analysis of trypanosoma spp. Detected from striped leaf-nosed bats (hipposideros vittatus) in zambia. *Int J Parasitol Parasites Wildl* **9**, 234–238 (2019).

507. Quintal, R. E., A. P. & Nunes, C. M. Leishmania spp. In didelphis albiventris and micoureus paraguayanus (didelphimorphia: Didelphidae) of brazil. *Vet Parasitol* **176**, 112–9 (2011).

508. Ramsey, G.-C., J. M. & Ibarra-Cerdena, C. N. Ecological connectivity of trypanosoma cruzi reservoirs and triatoma pallidipennis hosts in an anthropogenic landscape with endemic chagas disease. *PLoS One* **7**, e46013 (2012).

509. Rassi, K. M., Y.Azizi. The seminested PCR based detection of leishmania infantum infection in asymptomatic dogs in a new endemic focus of visceral leishmaniasis in iran. *Iranian Journal of Arthropod-Borne Diseases* **1**, 38–42 (2007).

510. Razzaghi Maensh, S. G., M.Mahabadi. Prevalence of canine visceral leishmaniasis in dogs at adrestan district detected by PCR. *Veterinary Research* **5**, 22–25 (2012).

511. Rodrigues, P., F. T. & Lopes, A. P. Seroprevalence of toxoplasma gondii and leishmania spp. In domestic donkeys from portugal. *Rev Bras Parasitol Vet* **28**, 172–176 (2019).

512. Rodriguez, T.-J., N. F. & Gutierrez, C. The role of wild rodents in the transmission of trypanosoma evansi infection in an endemic area of the canary islands (spain). *Vet Parasitol* **174**, 323–7 (2010).

513. Rodriguez, O. A. & Matta, N. E. Blood parasites in some birds from eastern plains of colombia. *Mem Inst Oswaldo Cruz* **96**, 1173–6 (2001).

514. Rossi, B., E. & Maroli, M. Seasonal phenology, host-blood feeding preferences and natural leishmania infection of phlebotomus perniciosus (diptera, psychodidae) in a high-endemic focus of canine leishmaniasis in rome province, italy. *Acta Trop* **105**, 158–65 (2008).

515. Rosypal, C.-V., A. C. & Lindsay, D. S. Serological survey of leishmania infantum and trypanosoma cruzi in dogs from urban areas of brazil and colombia. *Vet Parasitol* **149**, 172–7 (2007).

516. Ruiz, N., J. P. & Malele, I. The role of domestic animals in the epidemiology of human african trypanosomiasis in ngorongoro conservation area, tanzania. *Parasit Vectors* **8**, 510 (2015).

517. Ruiz-Pina, H. A. & Cruz-Reyes, A. The opossum didelphis virginiana as a synanthropic reservoir of trypanosoma cruzi in dzidzilche, yucatan, mexico. *Mem Inst Oswaldo Cruz* **97**, 613–20 (2002).

518. Saldana, C., A. & Chaves, L. F. Risk factors associated with trypanosoma cruzi exposure in domestic dogs from a rural community in panama. *Mem Inst Oswaldo Cruz* **110**, 936–44 (2015).

519. Saldana, S., A. & Calzada, J. E. A darker chromatic variation of rhodnius pallescens infected by specific genetic groups of trypanosoma rangeli and trypanosoma cruzi from panama. *Parasit Vectors* **11**, 423 (2018).

520. Sallemi, R., S. & Gharbi, M. Molecular prevalence and phylogenetic analysis of theileria annulata and trypanosoma evansi in cattle in northern tunisia. *Vet Med Sci* **4**, 17–25 (2018).

521. Salvatore, A., D. & Galuppi, R. Molecular evidence of leishmania infantum in ixodes ricinus ticks from dogs and cats, in italy. *Vet Ital* **50**, 307–12 (2014).

522. Salzer, P., J. S. & Gillespie, T. R. Impact of anthropogenic disturbance on native and invasive trypanosomes of rodents in forested uganda. *Ecohealth* **13**, 698–707 (2016).

523. Santaella, O., J. & Quinnell, R. J. Leishmania (viannia) infection in the domestic dog in chaparral, colombia. *Am J Trop Med Hyg* **84**, 674–80 (2011).

524. Sarquis, C.-C., O. & Lima, M. M. Eco-epidemiology of chagas disease in northeastern brazil: Triatoma brasiliensis, t. Pseudomaculata and rhodnius nasutus in the sylvatic, peridomestic and domestic environments. *Parasitol Res* **110**, 1481–5 (2012).

525. Sastre, F., N. & Altet, L. Detection of leishmania infantum in captive wolves from southwestern europe. *Vet Parasitol* **158**, 117–20 (2008).

526. Sazmand, E., A. & Joachim, A. Molecular identification of hemoprotozoan parasites in camels (camelus dromedarius) of iran. *Iran J Parasitol* **11**, 568–573 (2016).

527. Selmi, M. B. S., R.Dhibi. Evidence of natural infections with trypanosoma, anaplasma and babesia spp. In military livestock from tunisia. *Tropical Biomedicine* **36**, 742–757 (2019).

528. Shadomy, W., S. V. & Chappell, C. L. Combined use of enzyme-linked immunosorbent assay and flow cytometry to detect antibodies to trypanosoma cruzi in domestic canines in texas. *Clin Diagn Lab Immunol* **11**, 313–9 (2004).

529. Sharbatkhori, S., M. & Parvizi, P. Molecular variation in leishmania parasites from sandflies species of a zoonotic cutaneous leishmaniasis in northeast of iran. *J Vector Borne Dis* **51**, 16–21 (2014).

530. Sharma, B. K., A.Sharma. Incidence of haemoprotozoan infection in canines in and around mathura. *Veterinary Practitioner* **12**, 149–150 (2011).

531. Silbermayr, L., K. & Solkner, J. A novel qPCR assay for the detection of african animal trypanosomosis in trypanotolerant and trypanosusceptible cattle breeds. *PLoS Negl Trop Dis* **7**, e2345 (2013).

532. Silva Rde, R., C. & Alves, L. C. Detection of antibodies against leishmania infantum in cats (felis catus) from the state of pernambuco, brazil. *Rev Soc Bras Med Trop* **47**, 108–9 (2014).

533. Simo, S., G. & Asonganyi, T. Identification of different trypanosome species in the mid-guts of tsetse flies of the malanga (kimpese) sleeping sickness focus of the democratic republic of congo. *Parasit Vectors* **5**, 201 (2012).

534. Singh, P., N. & Kumar, R. A comparative evaluation of parasitological, serological and DNA amplification methods for diagnosis of natural trypanosoma evansi infection in camels. *Vet Parasitol* **126**, 365–73 (2004).

535. Singla, D., L. D. & Ba, M. S. Conventional and molecular diagnosis of haemo-protozoan infectionsin cattle and equids from republic of guinea and india. *Indian Journal of Animal Research* **52**, (2017).

536. Sobrino, F., R. & Gortazar, C. Characterization of widespread canine leishmaniasis among wild carnivores from spain. *Vet Parasitol* **155**, 198–203 (2008).

537. Solano-Gallego, L., L. & Breitschwerdt, E. A serological study of exposure to arthropod-borne pathogens in dogs from northeastern spain. *Vet Res* **37**, 231–44 (2006).

538. Solano-Gallego, M., L. & Ferrer, L. Prevalence of leishmania infantum infection in dogs living in an area of canine leishmaniasis endemicity using PCR on several tissues and serology. *J Clin Microbiol* **39**, 560–3 (2001).

539. Solano-Gallego, R., L. & Trotta, M. Detection of leishmania infantum DNA mainly in rhipicephalus sanguineus male ticks removed from dogs living in endemic areas of canine leishmaniosis. *Parasit Vectors* **5**, 98 (2012).

540. Spada, C., E. & Proverbio, D. Prevalence of leishmania infantum and co-infections in stray cats in northern italy. *Comp Immunol Microbiol Infect Dis* **45**, 53–8 (2016).

541. Sumbria, S., D. & Bal, M. S. Equine trypanosomosis in central and western punjab: Prevalence, haemato-biochemical response and associated risk factors. *Acta Trop* **138**, 44–50 (2014).

542. Sumbria, S., D. & Kaur, P. Comparative seroprevalence and risk factor analysis of trypanosoma evansi infection in equines from different agro-climatic zones of punjab (india). *Rev Sci Tech* **36**, 971–979 (2017).

543. Sun, G., K. & Chen, J. P. Prevalence of canine leishmaniasis in beichuan county, sichuan, china and phylogenetic evidence for an undescribed leishmania sp. In china based on 7SL RNA. *Parasit Vectors* **5**, 75 (2012).

544. Szpeiter, F., B. B. & Marcili, A. Bat trypanosomes from tapajos-arapiuns extractive reserve in brazilian amazon. *Rev Bras Parasitol Vet* **26**, 152–158 (2017).

545. Tadesse, A. & Tsegaye, B. Bovine trypanosomosis and its vectors in two districts of bench maji zone, south western ethiopia. *Trop Anim Health Prod* **42**, 1757–62 (2010).

546. Tafese, M., W. & Fentahun, T. Prevalence of bovine trypanosomosis and its vectors in two districts of east wollega zone, ethiopia. *Onderstepoort J Vet Res* **79**, E1–4 (2012).

547. Kante Tagueu, F., S. & Simo, G. Prevalence of sodalis glossinidius and different trypanosome species in glossina palpalis palpalis caught in the fontem sleeping sickness focus of the southern cameroon. *Parasite* **25**, 44 (2018).

548. Tamay-Segovia, B.-D., Paulino & Retana-Guiascón, O. G. Presence of virginia opossum (didelphis virginiana) and pic (triatoma dimidiata) infected with trypanosoma cruzi in urban areas: Preliminary evaluation in the city of campeche, mexico. *Austral journal of veterinary sciences* **49**, 35–38 (2017).

549. Tanczos, B., B. & Farkas, R. First record of autochthonous canine leishmaniasis in hungary. *Vector Borne Zoonotic Dis* **12**, 588–94 (2012).

550. Tehseen, J., Sonia & Qamar, M. F. Field investigation of trypanosoma evansi and comparative analysisof diagnostic tests in horses from bahawalpur, pakistan. *Turkish Journal of Veterinary and Animal Sciences* **41**, 288–293 (2017).

551. Tomas-Perez, K., M. & Fisa, R. First report of natural infection in hedgehogs with leishmania major, a possible reservoir of zoonotic cutaneous leishmaniasis in algeria. *Acta Trop* **135**, 44–9 (2014).

552. Tonelli, T., G. B. & Andrade Filho, J. D. Leishmania (viannia) braziliensis infection in wild small mammals in ecotourism area of brazil. *PLoS One* **12**, e0190315 (2017).

553. Tono, F., Rafi Rabecca & Ibitoye, E. B. Presence of trypanosome species and anemic status of dogs in zuru, nigeria. *Macedonian Veterinary Review* **38**, 217–222 (2015).

554. Toz, E., S. O. & Ozbel, Y. [An epidemiological study on canine leishmaniasis (CanL) and sand flies in northern cyprus]. *Turkiye Parazitol Derg* **37**, 107–12 (2013).

555. Toz, N. E., S. O.Sakru. Serological and entomological survey of zoonotic visceral leishmaniasis in denizli province, aegean region, turkey. *New Microbiologica* **32**, 93–100 (2009).

556. Trindade, N. A. de A., C. N. D.da Cunha. Cunha. Immunofluorescent antibody test (IFAT) for trypanosoma cruzi in dogs from urban and rural areas of pelotas, RS. *Boletim De Industria Animal* **72**, 111–116 (2015).

557. Tsakmakidis, A., Iota & Diakou, A. Leishmania infection in rodents in greece. *Trop Med Int Health* **22**, 1523–1532 (2017).

558. Valadas, M., S. & Gennari, S. M. Occurrence of antibodies anti-neospora caninum, anti-toxoplasma gondii, and anti-leishmania chagasi in serum of dogs from para state, amazon, brazil. *Parasitol Res* **107**, 453–7 (2010).

559. Valkiūnas, I., G. & Shapoval, A. P. High prevalence of blood parasites in hawfinch coccothraustes coccothraustes. *Journal of Natural History* **37**, 2647–2652 (2003).

560. Valkiunas, I., G. & Causey, D. Additional observations on blood parasites of birds in costa rica. *J Wildl Dis* **40**, 555–61 (2004).

561. Valkiunas, S., G. & Smith, T. B. Further observations on the blood parasites of birds in uganda. *J Wildl Dis* **41**, 580–7 (2005).

562. Villagran, J. A. de D., M. E.Martinez-Ibarra. Pathological alterations and prevalence of trypanosoma cruzi in opossums from western mexico. *Boletin De Malariologia Y Salud Ambiental* **51**, 87–88 (2011).

563. Villena, G.-P., F. E. & Ampuero, J. S. First report of trypanosoma cruzi infection in salivary gland of bats from the peruvian amazon. *Am J Trop Med Hyg* **99**, 723–728 (2018).

564. Dohlen, C. von, A. R. & Sharma, R. N. Prevalence of antibodies against visceralizing leishmania spp. In brown rats from grenada, west indies. *Vet World* **11**, 1321–1325 (2018).

565. Votypka, P., J. & Petrzelkova, K. J. An unexpected diversity of trypanosomatids in fecal samples of great apes. *Int J Parasitol Parasites Wildl* **7**, 322–325 (2018).

566. Voyvoda, S. T., H.Pasa. Prevalence of leishmania infantum and dirofilaria immitis infection in dogs in aydin province and the town of selcuk, izmir, turkey. *Turkish Journal of Veterinary & Animal Sciences* **28**, 1105–1111 (2004).

567. Wang, H., J. Y. & Chen, H. T. The prevalence of canine leishmania infantum infection in western china detected by PCR and serological tests. *Parasit Vectors* **4**, 69 (2011).

568. Wong, S. M., Y. Y. & Dumonteil, E. Molecular epidemiology of trypanosoma cruzi and triatoma dimidiata in costal ecuador. *Infect Genet Evol* **41**, 207–212 (2016).

569. Yabsley, M. J. & Noblet, G. P. Seroprevalence of trypanosoma cruzi in raccoons from south carolina and georgia. *J Wildl Dis* **38**, 75–83 (2002).

570. Yefi-Quinteros, M.-S. M., E. & Cattan, P. E. Trypanosoma cruzi load in synanthropic rodents from rural areas in chile. *Parasit Vectors* **11**, 171 (2018).

571. Zanet, S., S. & Ferroglio, E. Epidemiology of leishmania infantum, toxoplasma gondii, and neospora caninum in rattus rattus in absence of domestic reservoir and definitive hosts. *Vet Parasitol* **199**, 247–9 (2014).

572. Zangooie, M. K., F.Ganjali. Molecular detection of trypanosoma evansi based on ITS1 rDNA gene in camelus dromedarius in sistan region, iran. *Tropical Biomedicine* **35**, 1140–1147 (2018).

573. Zhang, G., J. R. & Chen, J. P. Molecular detection, identification and phylogenetic inference of leishmania spp. In some desert lizards from northwest china by using internal transcribed spacer 1 (ITS1) sequences. *Acta Trop* **162**, 83–94 (2016).

574. Zhao, Y., G. H. & Wang, H. F. Epidemiological investigation of asymptomatic dogs with leishmania infection in southwestern china where visceral leishmaniasis is intractable. *Korean J Parasitol* **54**, 797–801 (2016).

575. Zivicnjak, M., T. & Baric-Rafaj, R. A seroepidemiologic survey of canine visceral leishmaniosis among apparently healthy dogs in croatia. *Vet Parasitol* **131**, 35–43 (2005).

576. Zivicnjak, F. K., T.Martinkovic. Serological and entomological studies of canine leishmaniosis in croatia. *Veterinarski Arhiv* **81**, 99–110 (2011).

577. Zoghlami, C., Z. & Zhioua, E. Interaction between canine and human visceral leishmaniases in a holoendemic focus of central tunisia. *Acta Trop* **139**, 32–8 (2014).

578. Aisien, M., Aigbirior, P. O., Ovwah, E. & edo-taiwo, O. Blood parasites of some anurans from southern nigeria. *Tropical biomedicine* **32**, 598–607 (2015).

579. Arjona-Jiménez, G. *et al.* Antibodies of trypanosoma cruzi, leishmania mexicana and leishmania braziliensis in domiciled dogs in tabasco, mexico. *Revista MVZ Córdoba* **21**, 5828 (2017).

580. Del Rio, L. *et al.* Evidence for widespread leishmania infantum infection among wild carnivores in l. Infantum periendemic northern spain. *Preventive Veterinary Medicine* **113**, 435 (2014).

581. Mossaad, E. *et al.* The incrimination of three trypanosome species in clinically affected german shepherd dogs in sudan. *Parasitology Research* **116**, (2017).

582. Corrêa, L., Oliveira, M. S., Tavares-Dias, M. & Ceccarelli, P. Infections of hypostomus spp. By trypanosoma spp. And leeches: A study of hematology and record of these hirudineans as potential vectors of these hemoflagellates. *Revista Brasileira de Parasitologia Veterinária* **25**, (2016).

583. Bekele, M. & Nasir, M. Prevalence and host related risk factors of bovine trypanosomosis in hawagelan district, west wellega zone, western ethiopia. *African Journal of Agricultural Research* **6**, 5055–5060 (2011).

584. Daflon-Teixeira, N. *et al.* Multiple approaches to address potential risk factors of chagas disease transmission in northeastern brazil. *The American Journal of Tropical Medicine and Hygiene* **100**, 296–302 (2019).

585. Gurgel-Gonçalves, R. *et al.* Enzootic transmission of trypanosoma cruzi and t. Rangeli in the federal district of brazil. *Revista do Instituto de Medicina Tropical de São Paulo* **46**, 323–30 (2004).

586. Ferreira, M. L. & Avenant-Oldewage, A. Notes on the occurrence of trypanosoma sp. (Kinetoplastida: Trypanosomatidae) in freshwater fishes from south africa. *Onderstepoort Journal of Veterinary Research* **80**, (2013).

587. Lemos, M. *et al.* Phylogenetic and morphological characterization of trypanosomes from brazilian armoured catfishes and leeches reveal high species diversity, mixed infections and a new fish trypanosome species. *Parasites and Vectors* **8**, (2015).

588. Molina, J. P. *et al.* Trypanosomatids (protozoa: Kinetoplastida) in three species of armored catfish from mogi-guaçu river, pirassununga, são paulo, brazil. *Revista Brasileira de Parasitologia Veterinaria* **25**, 131–141 (2016).

589. Valdivia, H. *et al.* Natural leishmania infection of lutzomyia auraensis in madre de dios, peru, detected by a fluorescence resonance energy transfer-based real-time polymerase chain reaction. *The American journal of tropical medicine and hygiene* **87**, 511–7 (2012).

590. Fallah, M. R., E.Khanmohammadi. Serological survey and comparison of two polymerase chain reaction (PCR) assays with enzyme-linked immunosorbent assay (ELISA) for the diagnosis of canine visceral leishmaniasis in dogs. *African Journal of Biotechnology* **10**, 648–656 (2011).
