# Supplementary material for: Double trouble: trypanosomatids with two hosts have lower infection prevalence than single host trypanosomatids
Source: Evol Med Public Health. 2023 May 16;11(1):202–18. doi: 10.1093/emph/eoad014 (PMC10317189; doi:10.1093/emph/eoad014)
Supplement: eoad014_suppl_Supplementary_Table_S1 [file eoad014_suppl_supplementary_table_s1.docx]

**Supplementary Table1**: ***Searching terms utilised in this study***

| **Searching terms for all databases in (Web of Science)** | | **Hits** |
| --- | --- | --- |
| #1 | TS=(diversity OR incidence rate OR prevalence OR epidemiology)  Indexes=SCI-EXPANDED, SSCI, A&HCI, CPCI-S, CPCI-SSH, BKCI-S, BKCI-SSH, ESCI, CCR-EXPANDED, IC Timespan=All years | 1,811,220 |
| #2 | TS=(Trypanosomatids OR Trypanosoma)  Indexes=SCI-EXPANDED, SSCI, A&HCI, CPCI-S, CPCI-SSH, BKCI-S, BKCI-SSH, ESCI, CCR-EXPANDED, IC Timespan=All years | 36,226 |
| #3 | #2 AND #1  Indexes=SCI-EXPANDED, SSCI, A&HCI, CPCI-S, CPCI-SSH, BKCI-S, BKCI-SSH, ESCI, CCR-EXPANDED, IC Timespan=All years | 3,143 |
| #4 | TS= (Para-leishmania OR Leishmania OR Leptomonas OR Lotmaria OR Zelonia OR Borovskyia OR Crithidia OR Blastocrithidia OR Herpetomonas OR Phytomonas OR Wallaceina OR Jaenimonas OR Sergeia OR Paratrypansoma OR Lafontella OR Kentomonas OR Strigomonas OR Angomonas OR Blechomonas)  Indexes=SCI-EXPANDED, SSCI, A&HCI, CPCI-S, CPCI-SSH, BKCI-S, BKCI-SSH, ESCI, CCR-EXPANDED, IC Timespan=All years | 31,431 |
| #5 | #4 AND #1  Indexes=SCI-EXPANDED, SSCI, A&HCI, CPCI-S, CPCI-SSH, BKCI-S, BKCI-SSH, ESCI, CCR-EXPANDED, IC Timespan=All years | 2,656 |
| #6 combined search | #5 OR #3  Indexes=SCI-EXPANDED, SSCI, A&HCI, CPCI-S, CPCI-SSH, BKCI-S, BKCI-SSH, ESCI, CCR-EXPANDED, IC Timespan=All years | 5,414 |
| search stopped Jan 2020 | | |
| **Searching terms for database ( Scopus)** | | **Hits** |
| #1 | (diversity OR incidence rate OR prevalence OR epidemiology) AND (Trypanosomatids OR Trypanosoma) | 620 |
| #2 | ( ( diversity OR incidence AND rate OR prevalence OR epidemiology ) AND ( para-leishmania OR leishmania OR leptomonas OR lotmaria OR zelonia OR borovskyia OR crithidia OR blastocrithidia OR herpetomonas OR phytomonas) ) | 777 |
| #3 | (Wallaceina OR Jaenimonas OR Sergeia OR Paratrypansoma OR Lafontella OR Kentomonas OR Strigomonas OR Angomonas OR Blechomonas) | 152 |
| #4 combined search | #1 OR #2 OR #3 | 1511 |
| search stopped Jan 2020 | | |
| **Searching terms for database (Scopus) to investigate potential missing publications for fish group** | | **Hits** |
| #1 | (diversity OR incidence rate OR prevalence OR epidemiology) AND (Trypanosomatids OR Trypanosoma) |  |
| #2 | ( trypanosomatids OR trypanosoma ) AND ( fish OR leech ) |  |
| #3 | ( LIMIT-TO ( PUBYEAR ,  2020 ), OR ( LIMIT-TO ( PUBYEAR ,  2019 ), OR ( LIMIT-TO ( PUBYEAR ,  2018 ), OR ( LIMIT-TO ( PUBYEAR ,  2017 ), OR ( LIMIT-TO ( PUBYEAR ,  2016 ), OR ( LIMIT-TO ( PUBYEAR ,  2015 ), OR ( LIMIT-TO ( PUBYEAR ,  2014 ), OR ( LIMIT-TO ( PUBYEAR ,  2013 ), OR ( LIMIT-TO ( PUBYEAR ,  2012 ), OR ( LIMIT-TO ( PUBYEAR ,  2011 ), OR ( LIMIT-TO ( PUBYEAR ,  2010 ), OR ( LIMIT-TO ( PUBYEAR ,  2009 ), OR ( LIMIT-TO ( PUBYEAR ,  2008 ), OR ( LIMIT-TO ( PUBYEAR ,  2007 ), OR ( LIMIT-TO ( PUBYEAR ,  2006 ), OR ( LIMIT-TO ( PUBYEAR ,  2005 ), OR ( LIMIT-TO ( PUBYEAR ,  2004 ), OR ( LIMIT-TO ( PUBYEAR ,  2003 ), OR ( LIMIT-TO ( PUBYEAR ,  2002 ), OR ( LIMIT-TO ( PUBYEAR ,  2001 ), OR ( LIMIT-TO ( PUBYEAR ,  2000 ). |  |
|  | #1 and #2 and #3 | 755 |
| Search stopped Jan 2023, no missing studies during screening and selection phase. | | |
