# Supplementary material for: Double trouble: trypanosomatids with two hosts have lower infection prevalence than single host trypanosomatids
Source: Evol Med Public Health. 2023 May 16;11(1):202–18. doi: 10.1093/emph/eoad014 (PMC10317189; doi:10.1093/emph/eoad014)
Supplement: eoad014_suppl_Supplementary_Table_S2 [file eoad014_suppl_supplementary_table_s2.docx]

**Supplementary Table 2: *Summary of the meta-regressions***

| Regression | Moderator | Outer random | Inner random | Intercept | Description | presentation | Data source |
| --- | --- | --- | --- | --- | --- | --- | --- |
| A | Parasite group. | Study ID | Host type, parasite type | No | Summary purpose | Table1 | ***Full dataset(584studies )*** |
| B | Life-history of the parasite (dixenous, monoxenous, mixed). | Study ID | Host type, parasite type | Yes | Test whether monoxenous and dixenous parasites have different infection prevalence. | Fig3A | ***Full dataset(584studies)*** |
| C | Host group (insect, non-insect). | Study ID | Host type, parasite type | yes | Test whether insect hosts are more or less commonly infected than non-insect hosts of dixenous trypanosomatids. | Fig3B | ***Full dataset(584studies )*** |
| D | Host genus (insects infected with monoxenous trypanosomatids only). | Study ID | Host type, parasite type | yes | To compare insect hosts for differences in infection prevalence. | Fig4A | ***On subgroup of the dataset (36 studies)*** |
| E | Host genus (insects infected with dixenous trypanosomatids only). | Study ID | Host type, parasite type | yes | To compare insect hosts for differences in infection prevalence. | Fig4B | ***On subgroup of the dataset(102 studies)*** |
| F1 | Diagnostic method (insects only). | Study ID | Host type, parasite type | yes | Does diagnostic method affect the recorded prevalence. Because insects and non-insects use different methods, we separated these analyses. | Table2 | ***On subgroup of the dataset(133 studies)*** |
| F2 | Diagnostic method (non-insects only). | Study ID | Host type, parasite type | yes | Does diagnostic method affect the recorded prevalence in non-insect hosts. | Table2 | ***On subgroup of the dataset(466 studies)*** |
| F3 | Life-history of the parasite (dixenous, monoxenous) in insects only. | Study ID | Host type, parasite type | yes | Do monoxenous and dixenous trypanosomatids have different infection prevalence in insects. | Table2 | ***On subgroup of the dataset(99 studies)*** |
| F4 | Life-history of the parasite (dixenous, monoxenous) in flies only. | Study ID | Host type, parasite type | yes | Do monoxenous and dixenous trypanosomatids have different infection prevalence in flies. | Table2 | ***On subgroup of the dataset(71 studies)*** |
| F5 | Life-history of the parasite (dixenous, monoxenous) in true bugs. | Study ID | Host type, parasite type | yes | Do monoxenous and dixenous trypanosomatids have different infection prevalence in true bugs. | Table2 | ***On subgroup of the dataset(32 studies)*** |
| F6 | Host-group (insects and non-insects) in dixenous trypanosomatids only | Study ID | parasite type | yes | Do dixenous trypanosomatids have different infection prevalence in insects. | Table2 | ***On subgroup of the dataset(520 studies)*** |
| F7 | Host-group (insects and non-insects) in Leishmania only. | Study ID | parasite type | yes | Does Leishmania have different infection prevalence in insects. | Table2 | ***On subgroup of the dataset(252 studies)*** |
| F8 | Host-group (insects and non-insects) in Trypanosoma (excluding *T. cruzi*) only. | Study ID | parasite type | yes | Does Trypanosoma have different infection prevalence in insects | Table2 | ***On subgroup of the dataset(200studies)*** |
| F9 | Host-group (insects and non-insects) in *T. cruzi* only. | Study ID | parasite type | yes | Does *T. cruzi* have different infection prevalence in insects. | Table2 | ***On subgroup of the dataset(82 studies)*** |
| F10 | Wild vs managed bees | Study ID | Bee taxa, parasite taxa | yes | Do wild and managed bees have different infection prevalence. | Table2 | ***On subgroup of the dataset (30 studies)*** |
| F11 | Wild vs managed bumblebees | Study ID | Bee taxa, parasite taxa | yes | Do wild and managed bumblebees have different infection prevalence. | Table2 | ***On subgroup of the dataset(11 studies)*** |
| F12 | Wild vs managed honeybees | Study ID | Bee taxa, parasite taxa | yes | Do wild and managed honeybees have different infection prevalence. | Table2 | ***On subgroup of the dataset(20 studies)*** |
