# Supplementary material for: Double trouble: trypanosomatids with two hosts have lower infection prevalence than single host trypanosomatids
Source: Evol Med Public Health. 2023 May 16;11(1):202–18. doi: 10.1093/emph/eoad014 (PMC10317189; doi:10.1093/emph/eoad014)
Supplement: eoad014_suppl_Supplementary_Table_S3 [file eoad014_suppl_supplementary_table_s3.docx]

**Supplementary Table 3: Corrected cut-off of significance utilising ‘individuals’ as a unite for infections across all hosts**

| Description of null hypothesis | Meta-regression ID | p-value(k) | k | Adjusted cut-off after correction for multiple testing | conclusion |
| --- | --- | --- | --- | --- | --- |
| No significant difference between infections diagnosed via molecular versus (microscopic & culture based) tools among non-insects. | F2.1 | <.0001 | 1 | 0.002 | sig |
| No significant difference in infections diagnosed via serological versus (microscopic & culture based) tools among non-insects. | F2.2 | <.0001 | 2 | 0.004 | sig |
| No significant difference in *Leishmania* infections among insects and non-insects. | F7 | 0.0001 | 3 | 0.006 | sig |
| No significant difference in infections diagnosed via molecular versus (microscopic & culture based) tools among insects. | F1 | 0.0013 | 4 | 0.008 | sig |
| No significant difference in *dixenous* infections among insects and non-insects. | F6 | 0.0067 | 5 | 0.01 | sig |
| No significant difference between ‘monoxenous’ infections and ‘dixenous’ infections among all hosts. | B.1 | 0.0089 | 6 | 0.012 | sig |
| No significant difference between Dixenous infections and Monoxenous infections among insects. | F3 | 0.0097 | 7 | 0.014 | sig |
| No significant difference between ‘monoxenous’ infections and ‘dixenous’ infections among all hosts. | B.1 | 0.0089 | 8 | 0.016 | sig |
| No significant difference in infections between Trypanosoma and Endotrypanum genera | E.2 | 0.0727 | 9 | 0.018 | Not sig |
| No significant difference between ‘mixed trypanosomatids’ infections and ‘dixenous’ infections among all hosts. | B.2 | 0.0889 | 10 | 0.02 | Not sig |
| No significant difference in infections caused by Blastocrithidia_spp.vs ‘jaculum’_spp. | D.2 | 0.1865 | 11 | 0.022 | Not sig |
| No significant difference in prevalence of infections reported from insects and non-insects caused by *Trypanosoma spp. (*excluding *T.cruzi)* | F8 | 0.241 | 12 | 0.024 | Not sig |
| No significant difference in infections caused by Agomonas_spp. Vs ‘jaculum’_spp. | D.1 | 0.2714 | 13 | 0.026 | Not sig |
| No significant difference in infections caused by Leptomonas_spp. Vs ‘jaculum’_spp. | D.5 | 0.2829 | 14 | 0.028 | Not sig |
| No significant difference in infections caused by Endotrypanum or leishmania genera. | E.1 | 0.2937 | 15 | 0.030 | Not sig |
| No significant difference in *T.cruzi’s* infections among insects and non-insects | F9 | 0.3396 | 16 | 0.032 | Not sig |
| No significant difference in infections caused by Herpetomonas_spp. Vs ‘jaculum’_spp. | D.4 | 0.3654 | 17 | 0.034 | Not sig |
| No significant difference in infection prevalence caused by dixenous *spp.* compared to *m*onoxenous *spp among flies.* | F4 | 0.4566 | 18 | 0.036 | Not sig |
| No significant difference in infections between Phytomonas and Endotrypanum genera | E.3 | 0.4869 | 19 | 0.038 | Not sig |
| No significant difference in infections among managed and wild bees. | F10 | 0.7268 | 20 | 0.040 | Not sig |
| No significant difference in infection between dixenous *spp.*and monoxenous *spp among true bugs.* | F5 | 0.7883 | 21 | 0.042 | Not sig |
| No significant difference in infections among managed and wild honeybee. | F11 | 0.8357 | 22 | 0.044 | Not sig |
| No significant difference in infections caused by Crithidia_spp. And ‘jaculum’_spp. | D.3 | 0.8438 | 23 | 0.046 | Not sig |
| No significant difference in infections among managed and wild bumblebees. | F12 | 0.9096 | 24 | 0.048 | Not sig |
| No significant difference in infections caused by monoxenous vs ‘jaculum’_spp. | D.6 | 0.9372 | 25 | 0.05 | Not sig |

Number of comparisons from all meta-regressions(m=25), adjusted cut-off based on Benjamini-Hochberg method, the formula is (k/m)*0.05.

Where K is the index of p-value(K), arranged from smallest to largest, and m is the number of comparisons. We concluded that there is a significant p-value when p-value(k)< Adjusted cut-off. Whenever this is not met (p-value(k)> Adjusted cut-off) we conclude non- significance for all bigger p-values(k).

D.1-D.7 represents statistical comparisons of each level with the intercept level within a moderator within meta-regression D. Same goes for F1.1-F1.2 (meta-regression F1), E.1 - E.2 (meta-regression E) and B.1 and B.2 (meta-regression B).
